# Design, Synthesis and Biological Activity of Novel Methoxy- and Hydroxy-Substituted *N*-Benzimidazole-Derived Carboxamides

**DOI:** 10.3390/molecules29092138

**Published:** 2024-05-04

**Authors:** Anja Beč, Katarina Zlatić, Mihailo Banjanac, Vedrana Radovanović, Kristina Starčević, Marijeta Kralj, Marijana Hranjec

**Affiliations:** 1 Department of Organic Chemistry, Faculty of Chemical Engineering and Technology, University of Zagreb, Marulićev Trg 19, 10000 Zagreb, Croatia; abec@fkit.unizg.hr; 2 Division of Molecular Medicine, Ruđer Bošković Institute, Bijenička Cesta 54, 10000 Zagreb, Croatia; katarina.zlatic@irb.hr (K.Z.); marijeta.kralj@irb.hr (M.K.); 3 Pharmacology In Vitro, Selvita Ltd., Prilaz baruna Filipovića 29, 10000 Zagreb, Croatia; mihailo.banjanac@selvita.com (M.B.); vedrana.radovanovic@selvita.com (V.R.); 4 Department of Chemistry and Biochemistry, Faculty of Veterinary Medicine, University of Zagreb, Heinzelova 55, 10000 Zagreb, Croatia; kristina.starcevic@vef.hr

**Keywords:** antibacterial activity, antioxidative activity, antiproliferative activity, benzimidazoles, carboxamides, ROS

## Abstract

This work presents the design, synthesis and biological activity of novel *N*-substituted benzimidazole carboxamides bearing either a variable number of methoxy and/or hydroxy groups. The targeted carboxamides were designed to investigate the influence of the number of methoxy and/or hydroxy groups, the type of substituent placed on the N atom of the benzimidazole core and the type of substituent placed on the benzimidazole core on biological activity. The most promising derivatives with pronounced antiproliferative activity proved to be *N*-methyl-substituted derivatives with hydroxyl and methoxy groups at the phenyl ring and cyano groups on the benzimidazole nuclei with selective activity against the MCF-7 cell line (IC_50_ = 3.1 μM). In addition, the cyano-substituted derivatives **10** and **11** showed strong antiproliferative activity against the tested cells (IC_50_ = 1.2–5.3 μM). Several tested compounds showed significantly improved antioxidative activity in all three methods compared to standard BHT. In addition, the antioxidative activity of **9**, **10**, **32** and **36** in the cells generally confirmed their antioxidant ability demonstrated in vitro. However, their antiproliferative activity was not related to their ability to inhibit oxidative stress nor to their ability to induce it. Compound **8** with two hydroxy and one methoxy group on the phenyl ring showed the strongest antibacterial activity against the Gram-positive strain *E. faecalis* (MIC = 8 μM).

## 1. Introduction

In the case of organic molecules with several different functional groups of similar reactivity or with several identical functional groups, a protective group is introduced into a molecule by chemical modification to obtain chemoselectivity in a subsequent chemical reaction [1,2,3,4,5]. The hydroxyl functional group is susceptible to oxidation, acetylation and halogenation and therefore must be protected. The reactivity difference due to steric or electronic factors and the control of reaction conditions can often be exploited to attain a regioselective protection step [6,7]. A large number of different protecting groups have been developed, but the most commonly used are ethers, acetals (or ketals) and esters [8,9,10,11]. Methyl and benzyl ethers are widely utilized as protective groups due to their stability, such as the low reactivity of the methoxy and benzyloxy groups in leaving groups under nucleophilic conditions [12,13,14,15,16,17].

Many studies have shown that phenolic compounds play a very important role in the prevention of various diseases such as cancer, heart disease, diabetes and others. The number of hydroxyl groups and their position in the aromatic ring play an important role in enhancing their antioxidative activity. Moreover, the methoxy and carboxylic acid groups also have important effects on the antioxidative ability of phenolic compounds [18,19,20,21,22].

It has been widely demonstrated that oxidative stress plays an essential role in cancer development since high levels of reactive oxygen species (ROS) can trigger damage to biomolecules, promoting carcinogenesis [23,24,25,26].

In the last few decades, the synthesis and application of new antioxidants have been gaining importance due to the development of more promising and effective compounds relative to standard antioxidants such as β-carotene, vitamin A, vitamin C, etc.

**Figure 1:**
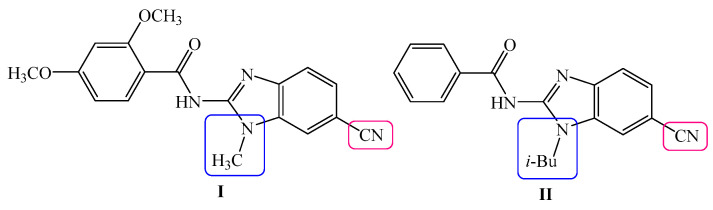
Previously synthesized *N*-substituted benzimidazole derived benzamides **I** and **II**.

Recently, we synthesized various benzimidazole/benzothiazole-2-carboxamides substituted with a variable number of methoxy and/or hydroxy moieties and bearing cyano-, nitro-, amidino- or amino-protonated groups. The presence of methoxy and hydroxy groups enhances the antioxidant activity of benzazole derivatives as they donate hydrogen atoms or electrons to stabilize free radicals. Some of the hydroxy-substituted carboxamide derivatives with potent antioxidant activity target specific pathways or molecules involved in oxidative stress without adversely affecting normal cellular processes, resulting in lower cytotoxicity [27,28,29,30]. Additionally, our intention was to study the influence of a substituent placed at the N atom of the benzimidazole core, and we synthesized *N*-substituted benzimidazole-2-carboxamides bearing different numbers of methoxy groups on the phenyl ring [31]. Isobutyl and methyl groups placed at the N atom had the most significant influence on the antioxidative and antiproliferative activity (Figure 1).

The above-mentioned considerations prompted us to extend our previous research by introducing electron-donating groups to the nitrogen atom and hydroxy groups to the phenyl ring. Within this manuscript, we present the design, synthesis and biological activity of novel hydroxy-substituted N-benzimidazole benzamide derivatives. By preventing oxidative damage, novel benzamide derivatives may indirectly inhibit aberrant cell growth without causing significant toxicity. Targeted compounds were prepared through the removal of methoxy and benzyloxy protective groups and were screened for their antioxidative capacity using several spectroscopic methods, as well as their antiproliferative and antibacterial activity.

## 2. Results and Discussion

### 2.1. Chemistry

**Scheme 1:**
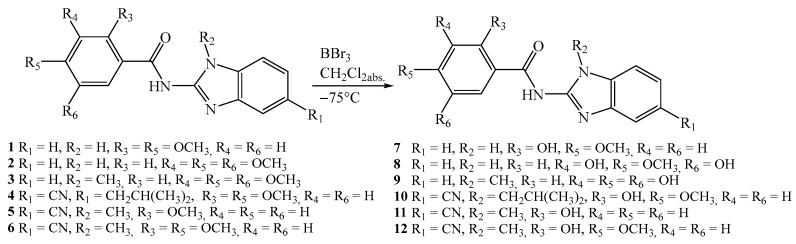
Synthesis of benzimidazole-derived benzamides **7**–**12**.

**Scheme 2:**
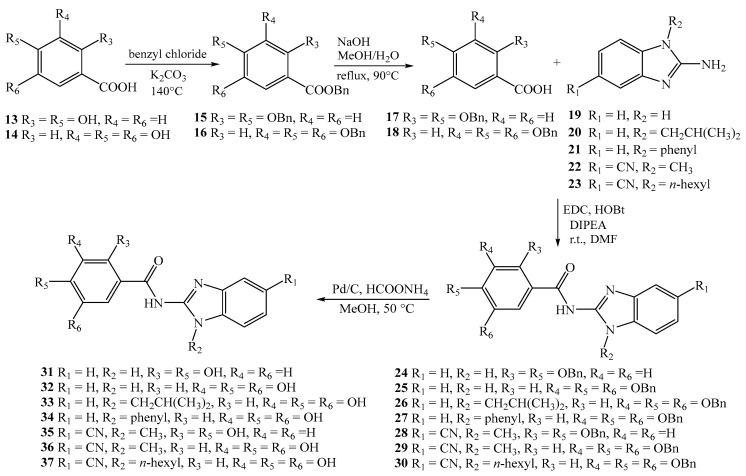
Synthesis of benzimidazole-derived benzamides **24**–**37**.

All the investigated benzamide derivatives were synthesized following the two synthetic procedures illustrated in reaction Scheme 1 and Scheme 2, using well-described and conventional organic synthetic methods [31].

The main precursors for the synthesis of targeted benzamides, the methoxy-substituted derivatives **1**–**6**, were prepared by previously published synthetic procedures [31,32].

To obtain the corresponding hydroxy-substituted benzamides, the removal of methoxy protecting groups was accomplished by using boron tribromide in absolute dichloromethane at −75 °C. The target derivatives **7**–**12** were prepared in relatively low yields (9–33%), and the reason for this may be an insufficiently aggressive reagent and the formation of unwanted byproducts. During the synthesis of derivatives **7**, **8**, **10** and **12**, one methoxy protecting group was successfully removed, while in derivatives **9** and **11**, all the methoxy protecting groups were successfully removed. Two-dimensional NOESY NMR spectra confirmed that the methoxy group at the position C-2 (R_3_) was successfully deprotected. According to Scheme 2, benzyl-protected benzoic acids **17** and **18** were prepared in an 88% overall yield using commercially available 2,4-dihydroxybenzoic acid, **26**, and 3,4,5-trihydroxybenzoic acid, **27**, as the starting materials for benzylation with benzyl chloride and basic hydrolysis. Newly prepared benzyl-protected *N*-substituted benzimidazole amides were synthesized in low-to-moderate yields by a simple coupling reaction with *N*-substituted 2-aminobenzimidazole and benzyl-protected benzoic acid, **17** and **18**, involving EDC.HCl and HOBt, used as carboxyl-activating agents. EDC.HCl is a carbodiimide used to activate carboxylic acid for amide or ester formation.

Hydroxybenzotrizole (HOBt) is mainly used to suppress racemization and to improve the efficiency of peptide synthesis. DIPEA was used as a base. The deprotection of the benzyl protective groups **24**–**30** was carried out by catalytic hydrogenation in the presence of 10% Pd/C in methanol to produce the corresponding deprotected derivatives (13–99%). The structures of all the newly prepared *N*-substituted benzimidazole benzamides were characterized by means of ^1^H and ^13^C NMR spectroscopy, mass spectrometry (Supplementary Materials) and elemental analysis.

### 2.2. Biological Activity

#### 2.2.1. Antiproliferative Activity against Various Cancer Cell Lines

The experiments were carried out on four human cell lines which are derived from three cancer types: H460 (lung carcinoma), HCT 116 (colorectal carcinoma), MCF-7 (human breast cancer) and the non-cancerous cell line HEK 293 (human embryonic kidney).

The results are expressed as IC_50_, the concentration required for 50% inhibition, and presented in Table 1. The results are compared with the known antiproliferative agents doxorubicin and etoposide. Compounds **7** and **12** showed low solubility in water, so the precipitation of these compounds during the incubation period was observed. Therefore, it is not clear whether the observed antiproliferative activity is due to the biological activity of the compounds or to precipitation. The 2-hydroxy-4-methoxy-substituted derivative **10** with an isobutyl chain on the N atom of the benzimidazole nucleus showed the most pronounced antiproliferative activity in vitro against all the cell lines in the low micromolar concentration range (IC_50_ = 2.2–4.4 µM). The 2-hydroxy-substituted derivative **11** with a methyl group on the N atom of the benzimidazole nucleus showed inhibitory activity against the cell lines HCT 116 (IC_50_ = 3.7 µM), MCF-7 (IC_50_ = 1.2 µM) and HEK 293 (IC_50_ = 5.3 µM).

The 2-hydroxy-4-methoxy-substituted derivative **12** with a methyl group on the N atom of the benzimidazole core showed selective activity against the MCF-7 cell line (IC_50_ = 3.1 µM). Also, selectivity towards the MCF-7 cell line was shown by the 3,4,5-trihydroxy-substituted derivative **36** with a methyl group on the N atom of the benzimidazole core (IC_50_ = 4.8 µM). Its analogue, the 2,4-dihydroxy-substituted derivative **35**, showed similar selective activity against the MCF-7 cell line (IC_50_ = 8.7 µM). The other derivatives showed weak-to-moderate antiproliferative activity, which may be a consequence of their low solubility in water due to pronounced lipophilicity or steric hindrance [33].

#### 2.2.2. Antioxidative Activity In Vitro

The synthesized benzazole acrylonitrile derivatives were screened for their antioxidative potency by using in vitro assays, namely DPPH and ABTS as free radical scavenging assays and FRAP as a ferric reducing/antioxidant power assay.

The well-known antioxidant butylated hydroxytoluene (BHT) was used as a standard for comparing activity during testing. The results are expressed as IC_50_ values and are presented in Table 2. The DPPH assay is a standard commonly used test for the in vitro assessment of the antioxidant capacity of novel compounds by using their ability to scavenge the stable radical 1,1-diphenyl-picrylhydrazyl. The results of the DPPH assay showed that most of the newly prepared compounds exhibited significantly improved (**8**, **9**, **32**, **33**, **34**, **36**, **37**) free radical scavenging activity compared to the standard. Another test used to evaluate the antioxidative capacity of novel compounds was the ABTS test, by which their activity was evaluated by scavenging the stable radical 3-ethylbenzthiazoline-6-sulphonic acid. In the ABTS test, most of the derivatives showed a weak interaction with the ABTS radical, with the exception of the 3,4,5-trihydroxy-substituted derivative **37** with an *n*-hexyl substituent on the *N* atom of the benzimidazole core, which showed pronounced antioxidative activity in comparison to the standard.

The derivatives **8**, **9**, **32**, **33**, **36** and **37** showed excellent in vitro antioxidant activity in the ferric reducing/antioxidant power measured by the FRAP assay, being significantly more active when compared to the standard BHT. The antioxidant activity of a substance is directly related to its reducing power. The FRAP assay directly correlates with antioxidant activity, so it is not surprising that the above compounds showed an exceptional ability to stabilize DPPH radicals. Although compound **34** showed exceptional activity in the DPPH assay, its reducing power in the FRAP assay was very low. This discrepancy suggests that while compound **34** is effective at scavenging stable free radicals, it may have limited capacity to reduce ferric ions.

The results of the antioxidant activity assays showed that hydroxy groups play an important role in improving the activity of these compounds, which is in line with previous studies by our research group [28].

#### 2.2.3. Antioxidant Ability in Cells

**Figure 2:**
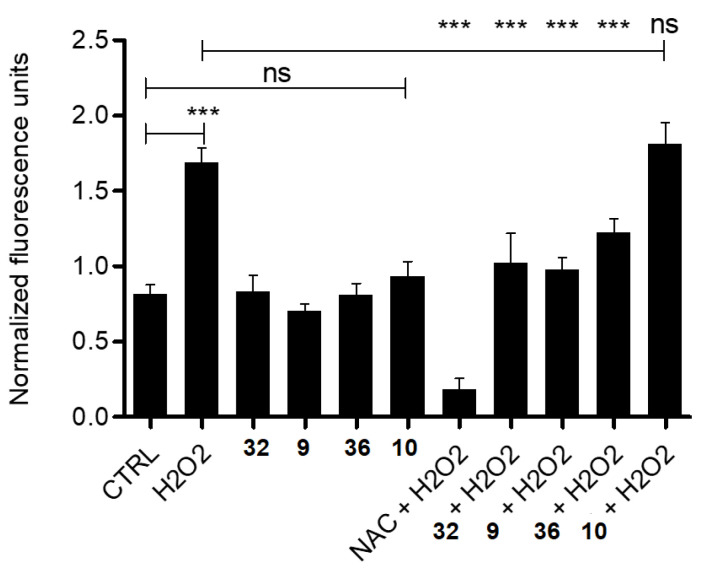
Antioxidative activity of selected systems. HCT 116 cells were treated with a combination of H_2_O_2_ (4 mM) and *N*-acetyl-*L*-cysteine (NAC, 10 mM) or the tested compounds (10 mM). Treatment with H_2_O_2_ (4 mM) alone was used as a control for ROS induction. The level of reactive oxygen species (ROS) was measured with the fluorescent dye DCFH-DA using a fluorimeter. The data presented here are the results of three independent measurements carried out in triplicate. A one-way ANOVA with Tukey’s post hoc test was used for statistical analysis, *** *p* < 0.001; ns—not significant.

We further selected compounds **9**, **10**, **32** and **36**, showing quite pronounced antioxidant capacity in the DPPH and FRAP assays, in order to test their pro- and antioxidant activity in tumor cells. In order to test the antioxidant activity of the selected compounds in tumor cells (Figure 2), we treated HCT 116 cells with *tert*-butyl hydroperoxide (TBHP), a substance commonly used for inducing oxidative stress in cells and tissues, alone or in combination with a known antioxidative agent, *N*-acetyl-*L*-cysteine (NAC), or the tested compounds. We measured the formation of oxidative stress byproducts using 2′,7′-dichlorodihydrofluorescein diacetate (DCFH-DA).

The results showed that none of the compounds affected the basal level of ROS in the cells (Figure 2). The tested compounds did not induce significant changes in the steady-state ROS levels under the experimental conditions. Derivatives **32**, **9** and **36** showed antioxidant activity by reducing the level of ROS in the cells, however, to a much lesser extent compared to the known antioxidant *N*-acetyl-*L*-cysteine (NAC). Despite exhibiting antioxidative activity, derivative **10** did not effectively scavenge ROS within the complex intracellular environment of the HCT 116 cells. Consequently, the antiproliferative activity of the tested compounds is neither related to their ability to induce oxidative stress nor to their ability to inhibit it.

#### 2.2.4. Antibacterial Activity In Vitro

The in vitro antibacterial activity of the synthesized benzamides **7**–**12** and **31**–**37** was evaluated against a panel of eight different bacterial strains [34]. The Gram-positive bacterial strains comprised *S. aureus*, *S. pneumoniae* and *E. faecalis,* and the panel of Gram-negative bacteria consisted of *E. coli*, *K. pneumoniae*, *A. baumannii* and *P. aeruginosa*. As reference drugs, the antibiotics ampicillin, azithromycin, ceftazidime, ciprofloxacin, gentamicin, meropenem and tetracycline were included. The results are expressed as MIC values (the lowest concentration at which bacterial growth is completely inhibited) and presented in Table 3. As presented in Table 3, the majority of derivatives lacked antibacterial activity, while some of the compounds showed moderate activity against certain bacterial strains. Compounds **7**, **8**, **11**, **12**, **31**, **34**, **35** and **37** showed selective antibacterial activity against the Gram-positive strain *E. faecalis*.

The most potent derivative was proven to be compound **8**, bearing two hydroxy and one methoxy group at the phenyl ring, with selective activity against the Gram-positive strain *E. faecalis* (MIC = 8 μM). The 3,4,5-trihydroxy-substituted derivative **37** with an *n*-hexyl chain on the N atom of the benzimidazole core showed the most pronounced antibacterial activity against the Gram-positive *S. aureus* and *E. faecalis* and the Gram-negative *E. coli* efflux del. strains (MIC = 16 μM). The 3,4,5-trihydroxy-substituted derivative **33** with an isobutyl chain and the derivative **36** with a methyl group on the N atom of the benzimidazole core showed moderate activity against the *S. aureus* strain (MIC = 32 μM).

## 3. Conclusions

This manuscript describes the design, synthesis and biological activity of novel *N*-substituted 2-benzimidazole-derived carboxamides with a variable number of methoxy and hydroxy groups placed at the phenyl ring. The main focus was on evaluating the type of substituent placed at the N atom as well as at the 5(6) position on the benzimidazole core and the type and number of substituents on the phenyl ring on the biological activity. This study’s emphasis on the substituents positioned at the benzimidazole core’s 5(6) position and N atom is essential for elucidating SAR, influencing the compound’s interaction with biological targets involved in oxidative stress and cell proliferation pathways.

All the compounds were tested for their antiproliferative, antioxidative and antibacterial activity in vitro. The obtained results confirmed that the type of substituents on the benzimidazole core and the type and number of substituents on the phenyl ring strongly impact biological activity.

Several of the tested compounds showed significant antiproliferative activity. The most promising antiproliferative activity was displayed by the *N*-methyl-substituted derivative bearing hydroxy and methoxy groups at the phenyl ring and the cyano group at the benzimidazole nuclei, with selective activity against the MCF-7 cell line (IC_50_ = 3.1 μM). The cyano-substituted derivatives **10** and **11** also showed strong antiproliferative activity but without selectivity among the tested cancer cells (IC_50_ = 1.2–5.3 μM). Additionally, some compounds showed significant antioxidative activity that was improved even compared to the standard, BHT, used in all three methods, but particularly in the ABTS test.

Concerning the ABTS assay, most of the derivatives showed a weak interaction with the ABTS radical, except derivative **37**. In addition, the antioxidative activity in the cells generally confirmed that the antiproliferative activity of the tested compounds **9**, **10**, **32** and **36** is not related to their ability to induce oxidative stress.

The majority of the tested derivatives lacked antibacterial activity. The most potent antibacterial activity was shown by compound **8**, bearing two hydroxy and one methoxy group at the phenyl ring, with selective activity against the Gram-positive strain *E. faecalis* (MIC = 8 μM).

In conclusion, this research is a good starting point toward producing even more effective compounds through several pathways, including substituting the cyano group with amidino moieties and replacing the benzothiazole nuclei with alternative scaffolds, which represent viable strategies for enhancing the effectiveness of the compounds. These approaches leverage structural modifications and SAR exploration to develop novel compounds with improved therapeutic potential for treating oxidative stress-related diseases and cancer.

## 4. Experimental Part

### 4.1. General Methods

All chemicals were purchased from commercial suppliers and were analytically pure. Melting points were recorded on the SMP11 Bibby apparatus (Reichert, Wien, Austria). The ^1^H and ^13^C NMR spectra were recorded on a Varian Bruker Advance III HD 400 MHz/54 mm Ascend instrument. All NMR spectra were measured in DMSO-*d_6_* solutions using TMS as an internal standard. Flash chromatography was performed on an Interchim PuriFlash^®^ (Interchim, Montluço, France) device on commercially available columns (Interchim PF-15SIHC-JP/ 4 and 12 g) filled with spherical silica gel (particle size 15 µm). All compounds were routinely checked by TLC with Merck silica gel 60F-254 glass plates, and the spots were detected under UV light. Elemental analysis for carbon, hydrogen and nitrogen were performed on a PerkineElmer 2400 elemental analyzer (Cotati, CA, USA). Where analyses are indicated only as symbols of elements, the analytical results obtained are within 0.4% of the theoretical value. Compounds **1**–**6** were prepared according to previously published experimental procedures, and all physico-chemical characteristics as well as NMR data are in accordance with previously published ones [31,32].

### 4.2. General Method for Preparation of Compounds ***7**–**12***

The corresponding methoxy-substituted compound (**1**–**6**) was dissolved in dry CH_2_Cl_2_ and cooled to –78 °C, and BBr_3_ was added under an argon atmosphere. The reaction mixture was stirred at room temperature overnight. The reaction was quenched by the addition of methanol; the solvent was evaporated under reduced pressure; and the residue was filtered off and purified by column chromatography (CH_2_Cl_2_/CH_3_OH) if needed.

*N-(1H-benzo[d]imidazol-2-yl)-2-hydroxy-4-methoxybenzamide* **7**

Compound **7** was prepared from **1** (0.15 g, 0.5 mmol), and 3.0 mL of BBr_3_ (3.0 mmol) was dissolved in absolute CH_2_Cl_2_ (20 mL) under an argon atmosphere to yield 0.02 g (16%) of white powder. m.p. 254–257 °C; ^1^H NMR (600 MHz, DMSO-*d_6_*): δ/ppm = 7.97 (d, 1H, *J* = 8.79 Hz, H_arom_), 7.68–7.62 (m, 2H, H_arom_), 7.41-7.38 (m, 2H, H_arom_), 6.64 (d, 1H, *J* = 8.87 Hz, H_arom_), 6.60 (d, 1H, *J* = 2.04 Hz, H_arom_), 3.83 (s, 3H, OCH_3_); ^13^C NMR (151 MHz, DMSO-*d*_6_): δ/ppm = 164.0, 159.5, 131.7, 128.1, 123.6, 112.2, 106.5, 100.4, 54.9; MS (ESI): *m*/*z* = 383,93 ([M+1]^+^); Anal. Calcd. for C_15_H_13_N_3_O_3_: C, 63.60; H, 4.63; N, 14.83. Found: C, 63.55; H, 4.67; N, 14.89%.

*N-(1H-benzo[d]imidazol-2-yl)-3,5-dihydroxy-4-methoxybenzamide* **8**

Compound **8** was prepared from **2** (0.17 g, 0.5 mmol), and 4.6 mL of BBr_3_ (4.6 mmol) was dissolved in absolute CH_2_Cl_2_ (25 mL) under an argon atmosphere to yield 0.02 g (12%) of white powder. m.p. 233–238 °C; ^1^H NMR (600 MHz, DMSO-*d_6_*): δ/ppm = 12.60–12.50 (m, 3H, OH, NH_amide_), 7.72–7.67 (m, 2H, H_arom_), 7.36–7.30 (m, 3H, H_arom_), 6.94 (d, 1H, *J* = 1.67 Hz, H_arom_), 3.80 (s, 3H, OCH_3_); ^13^C NMR (151 MHz, DMSO-*d*_6_): δ/ppm = 171.2, 146.7, 145.8, 145.0, 140.8, 128.3, 125.5, 122.7, 122.7, 112.2, 112.1, 102.2, 98.9, 98.8, 55.2; Anal. Calcd. for C_15_H_13_N_3_O_4_: C, 60.20; H, 4.38; N, 14.04. Found: C, 60.26; H, 4.45; N, 14.09%.

*3,4,5-trihydroxy-N-(1-methyl-1H-benzo[d]imidazol-2-yl)benzamide* **9**

Compound **9** was prepared from **3** (0.14 g, 0.4 mmol), and 3.76 mL of BBr_3_ (3.8 mmol) was dissolved in absolute CH_2_Cl_2_ (20 mL) under an argon atmosphere to yield 0.08 g (33%) of pink powder. m.p. 264–270 °C; ^1^H NMR (600 MHz, DMSO-*d_6_*): δ/ppm = 9.37 (bs, 2H, OH), 7.80–7.70 (m, 2H, H_arom_), 7.52-7.42 (m, 2H, H_arom_), 7.14 (s, 2H, H_arom_), 3.91 (s, 3H, CH_3_); ^13^C NMR (151 MHz, DMSO-*d_6_*): δ/ppm = 145.0, 138.2, 129.4, 127.9, 124.2, 123.9, 112.9, 110.7, 107.9, 29.9; MS (ESI): *m*/*z* = 299,95 ([M+1]^+^); Anal. Calcd. for C_15_H_13_N_3_O_4_: C, 60.20; H, 4.38; N, 14.04. Found: C, 60.26; H, 4.44; N, 13.98%.

*N-(5-cyano-1-isobutyl-1H-benzo[d]imidazol-2-yl)-2-hydroxy-4-methoxybenzamide* **10**

Compound **10** was prepared from **4** (0.08 g, 0.2 mmol), and 1.3 mL of BBr_3_ (1.3 mmol) was dissolved in absolute CH_2_Cl_2_ (20 mL) under an argon atmosphere to yield 0.01 g (13%) of white powder. m.p. 222–225 °C; ^1^H NMR (300 MHz, DMSO-*d_6_*): δ/ppm = 13.86 (s, 1H, OH), 13.17 (s, 1H, NH_amide_), 8.02 (d, 1H, *J* = 8.68 Hz, H_arom_), 7.94 (s, 1H, H_arom_), 7.87 (d, 1H, *J* = 8.27 Hz, H_arom_), 7.80 (d, 1H, *J* = 8.18 Hz, H_arom_), 6.54 (d, 1H, *J* = 8.69 Hz, H_arom_), 6.49 (s, 1H, H_arom_), 4.09 (d, 2H, *J* = 6.99 Hz, CH_2_), 3.84 (s, 3H, CH_3_), 2.36–2.26 (m, 1H, CH), 1.01 (d, 6H, *J* = 6.46 Hz, CH_3_); ^13^C NMR (75 MHz, DMSO-*d*_6_): δ/ppm = 164.0, 162.6, 151.7, 133.2, 131.6, 128.9, 127.3, 119.2, 115.8, 112.4, 111.4, 106.2, 104.7, 100.6, 99.5, 55.3, 49.5, 27.5, 19.7 (2C); Anal. Calcd. for C_20_H_20_N_4_O_3_: C, 65.92; H, 5.53; N, 15.38. Found: C, 65.87; H, 5.46; N, 15.42%.

*N-(5-cyano-1-methyl-1H-benzo[d]imidazol-2-yl)-2-hydroxybenzamide* **11**

Compound **11** was prepared from **5** (0.24 g, 0.8 mmol), and 2.3 mL of BBr_3_ (2.3 mmol) was dissolved in absolute CH_2_Cl_2_ (30 mL) under an argon atmosphere to yield 0.05 g (22%) of white powder. m.p. 218–222 °C; ^1^H NMR (600 MHz, DMSO-*d_6_*): δ/ppm = 13.60 (s, 1H, OH), 13.12 (s, 1H, NH_amide_), 8.09 (dd, 1H, *J_1_* = 1.68 Hz, *J_2_* = 8.27 Hz, H_arom_), 7.88 (s, 1H, H_arom_), 7.77 (s, 2H, H_arom_), 7.43–7.39 (m, 1H, H_arom_), 6.91-6.87 (m, 2H, H_arom_), 3.72 (s, 3H, CH_3_); MS (ESI): *m*/*z* = 293,03 ([M+1]^+^); Anal. Calcd. for C_16_H_12_N_4_O_2_: C, 65.75; H, 4.14; N, 19.17. Found: C, 65.81; H, 4.10; N, 19.13%.

*N-(5-cyano-1-methyl-1H-benzo[d]imidazol-2-yl)-2-hydroxy-4-methoxybenzamide* **12**

Compound **12** was prepared from **6** (0.13 g, 0.4 mmol), and 2.3 mL of BBr_3_ (2.3 mmol) was dissolved in absolute CH_2_Cl_2_ (20 mL) under an argon atmosphere to yield 0.02 g (9%) of white powder. m.p. 234–236 °C; ^1^H NMR (600 MHz, DMSO-*d_6_*): δ/ppm = 13.76 (s, 1H, OH), 13.01 (s, 1H, NH_amide_), 8.00 (d, 1H, *J* = 8.79 Hz, H_arom_), 7.85 (s, 1H, H_arom_), 7.74 (d, 2H, *J* = 1.13 Hz, H_arom_), 6.47 (dd, 1H, *J*_1_ = 2.47 Hz, *J*_2_ = 8.78 Hz, H_arom_), 6.39 (d, 1H, *J* = 2.50 Hz, H_arom_), 3.79 (s, 3H, OCH_3_), 3.70 (s, 3H, CH_3_); ^13^C NMR (151 MHz, DMSO-*d_6_*): δ/ppm = 164.5, 163.3, 152.4, 133.8, 132.5, 132.2, 127.8, 119.8, 116.1, 112.9, 111.5, 111.4, 106.6, 105.1, 102.8, 101.1, 55.8, 29.4; MS (ESI): *m*/*z* = 323,07 ([M+1]^+^); Anal. Calcd. for C_17_H_14_N_4_O_3_: C, 63.35; H, 4.38; N, 17.38. Found: C, 63.46; H, 4.31; N, 17.42%.

### 4.3. General Method for Preparation of Compounds ***15**–**16***

To a solution of anhydrous K_2_CO_3_ in DMSO, 2,4-dihydroxybenzoic acid **13** or 3,4,5-trihydroxybenzoic acid **14** was added under stirring and heated to 140 °C.

Then, benzyl chloride was added dropwise under an argon atmosphere and then reacted at 140 °C for 3 h. After cooling, the reaction mixture was diluted with cold-distilled water and extracted with CH_2_Cl_2_. The organic layer was evaporated under reduced pressure to give rise to a solid product. This product was suspended in MeOH, filtered off and washed with a suitable amount of MeOH.

*Benzyl 2,4-bis(benzyloxy)benzoate* **15**

Compound **15** was prepared from **13** (1.00 g, 6.5 mmol), K_2_CO_3_ (2.75 g, 20.1 mmol) and 3.73 mL of benzyl chloride (35.7 mmol) dissolved in 20 mL of DMSO to yield 1.57 g (57%) of white powder. m.p. 111–115 °C; ^1^H NMR (300 MHz, DMSO-*d_6_*): δ/ppm = 7.78 (d, 1H, *J* = 8.67 Hz, H_arom_), 7.50–7.29 (m, 15H, H_arom_), 6.88 (d, 1H, *J* = 2.08 Hz, H_arom_), 6.71 (dd, 1H, *J*_1_ = 2.19 Hz, *J*_2_ = 8.69 Hz, H_arom_), 5.27 (s, 2H, CH_2_), 5.20 (s, 2H, CH_2_), 5.18 (s, 2H, CH_2_); ^13^C NMR (DMSO-*d*_6_, 151 MHz): δ/ppm = 164.9, 163.0, 159.6, 136.7, 136.4, 136.4, 133.3, 128.5, 128.4, 128.3, 128.0, 127.9, 127.8, 127.8, 127.6, 127.1, 112.2, 106.5, 101.1, 69.7, 69.6, 65.6; Anal. Calcd. for C_28_H_24_O_4_: C, 79.23; H, 5.70. Found: C, 79.31; H, 5.64%.

*Benzyl 3,4,5-tris(benzyloxy)benzoate* **16**

Compound **16** was prepared from **14** (1.00 g, 5.9 mmol), K_2_CO_3_ (3.33 g, 24.1 mmol) and 5 mL of benzyl chloride (43.5 mmol) dissolved in 20 mL of DMSO to yield 2.29 g (74%) of white powder. m.p. 108–112 °C; ^1^H NMR (600 MHz, DMSO-*d_6_*): δ/ppm = 7.47–7.44 (m, 4H, H_arom_), 7.44–7.31 (m, 15H, H_arom_), 7.30-7.25 (m, 3H, H_arom_), 5.33 (s, 2H, CH_2_), 5.19 (s, 4H, CH_2_), 5.06 (s, 2H, CH_2_); ^13^C NMR (DMSO-*d*_6_, 151 MHz): δ/ppm = 166.2, 151.3, 145.9, 140.3, 136.7, 136.5, 136.2, 127.8, 127.7, 127.5, 127.5, 127.3, 127.2, 126.9, 126.9, 125.4, 73.6, 69.6, 69.5; Anal. Calcd. for C_35_H_30_O_5_: C, 79.23; H, 5.70. Found: C, 79.29; H, 5.66%.

### 4.4. General Method for Preparation of Compounds ***17**–**18***

To a solution of NaOH in distilled water, benzyl 2,4-bis(benzyloxy)benzoate 15 or benzyl 3,4,5-tris(benzyloxy)benzoate 16 and MeOH was added, and the mixture was refluxed at 90 °C for 2 h. After cooling, the solution was poured into 1.2 N HCl. The precipitate was filtered, washed with distilled water, and dried in vacuo.

*2,4-Bis(benzyloxy)benzoic acid* **17**

Compound **17** was prepared from **15** (1.00 g, 2.3 mmol), 1.48 g of NaOH in 3.7 mL of H_2_O, and 20 mL of MeOH to yield 0.58 g (74%) of white powder. m.p. 113–115 °C; ^1^H NMR (300 MHz, DMSO-*d_6_*): δ/ppm = 7.71 (d, 1H, *J* = 8.56 Hz, H_arom_), 7.52–7.31 (m, 10H, H_arom_), 6.81 (d, 1H, *J* = 1.78 Hz, H_arom_), 6.67 (dd, 1H, *J*_1_ = 2.00 Hz, *J*_2_ = 8.67 Hz, H_arom_), 5.19 (s, 2H, CH_2_), 5.16 (s, 2H, CH_2_); ^13^C NMR (DMSO-*d*_6_, 151 MHz): δ/ppm = 166.5, 162.4, 159.3, 136.9, 136.5, 133.0, 128.4, 128.3, 128.0, 127.8, 127.5, 127.0, 113.9, 106.2, 101.2, 69.6, 69.5; Anal. Calcd. for C_21_H_18_O_4_: C, 75.43; H, 5.43. Found: C, 75.47; H, 5.39%.

*3,4,5-Tris(benzyloxy)benzoic acid* **18**

Compound **18** was prepared from **16** (2.29 g, 4.3 mmol) and 3.40 g of NaOH dissolved in 8.5 mL of H_2_O and 12 mL of MeOH to yield 1.69 g (88%) of white powder. m.p. 115–117 °C; ^1^H NMR (600 MHz, DMSO-*d_6_*): δ/ppm = 7.47 (d, 4H, *J* = 7.20 Hz, H_arom_), 7.42-7.36 (m, 6H, H_arom_), 7.36–7.30 (m, 4H, H_arom_), 7.31–7.24 (m, 3H, H_arom_), 5.13 (s, 4H, CH_2_), 4.99 (s, 2H, CH_2_); ^13^C NMR (DMSO-*d*_6_, 151 MHz): δ/ppm = 166.2, 151.4, 140.3, 136.8, 136.2, 127.8, 127.6, 127.6, 127.5, 127.3, 126.9, 126.9, 125.4, 107.6, 73.6, 69.6; Anal. Calcd. for C_28_H_24_O_5_: C, 76.35; H, 5.49. Found: C, 76.31; H, 5.54%.

### 4.5. General Method for Preparation of Compounds ***24**–**30***

To a stirring solution of 2,4-bis(benzyloxy)benzoic acid **17** or 3,4,5-tris(benzyloxy)- benzoic acid **18** in 5 mL of DMF, EDC.HCl, HOBt, DIPEA and the corresponding 2-aminobenzimidazole were added. The reaction was stirred at room temperature for 24 h.

Excess water was added and extracted with ethyl acetate. The organic layer was evaporated under reduced pressure to give rise to a solid product.

*N-(1H-benzo[d]imidazol-2-yl)-2,4-bis(benzyloxy)benzamide* **24**

Compound **24** was prepared from **17** (0.10 g, 0.5 mmol), **19** (0.8 g, 0.5 mmol), EDC.HCl (0.23 g, 1.1 mmol), HOBt (0.14 g, 0.5 mmol) and DIPEA (0.22 mL, 1.5 mmol) to yield 0.02 g (15%) of pink powder. m.p. 174–175 °C; ^1^H NMR (300 MHz, DMSO-*d*_6_): δ/ppm = 12.23 (s, 1H, NH_benzimidazole_) 11.06 (s, 1H, NH_amide_) 7.89 (d, 1H, *J* = 8.67 Hz, H_arom_), 7.50–7.34 (m, 14H, H_arom_), 6.98 (d, 1H *J* = 1.88 Hz, H_arom_), 6.83 (dd, 1H, *J*_1_ = 1.89 Hz, *J*_2_ = 8.69 Hz, H_arom_), 5.40 (s, 2H, CH_2_), 5.22 (s, 2H, CH_2_); ^13^C NMR (75 MHz, DMSO-*d*_6_): δ/ppm = 163.7, 162.8, 157.9, 146.2, 136.4 (2C), 136.0 (2C), 132.4, 128.6 (2C), 128.5 (2C), 128.1 (2C), 127.9 (2C), 127.6 (2C), 121.9, 121.0, 114.5, 111.3, 107.3, 101.0 (2C), 70.5, 69.7; Anal. Calcd. for C_28_H_23_N_3_O_3_: C, 74.82; H, 5.16; N, 9.35. Found: C, 74.88; H, 5.10; N, 9.28%.

*N-(1H-benzo[d]imidazol-2-yl)-3,4,5-tris(benzyloxy)benzamide* **25**

Compound **25** was prepared from **18** (0.33 g, 0.8 mmol), **19** (0.12 g, 0.9 mmol), EDC.HCl (0.28 g, 1.4 mmol), HOBt (0.01 g, 0.09 mmol) and DIPEA (0.2 mL, 1.5 mmol) to yield 0.06 g (15%) of white powder. m.p. 197–202 °C; ^1^H NMR (400 MHz, DMSO-*d_6_*): δ/ppm = 12.20 (s, 1H, NH_amide_), 7.64 (s, 1H, H_arom_), 7.51-7.49 (m, 2H, H_arom_), 7.48–7.46 (m, 3H, H_arom_), 7.45–7.33 (m, 11H, H_arom_), 7.31–7.27 (m, 1H, H_arom_), 7.15–7.11 (m, 1H, H_arom_), 5.25 (s, 2H, CH_2_), 5.19 (s, 2H, CH_2_), 5.05 (d, 2H, *J* = 4.70 Hz, H_arom_); ^13^C NMR (151 MHz, DMSO-*d*_6_): δ/ppm = 151.9, 138.0, 137.4, 135.5, 135.3, 134.8, 130.5, 129.7, 128.9, 128.9, 128.8, 128.6, 128.5, 128.3, 128.0, 127.1, 74.6, 70.3; Anal. Calcd. for C_35_H_29_N_3_O_4_: C, 75.66; H, 5.26; N, 7.56. Found: C, 75.59; H, 5.20; N, 7.62%.

*3,4,5-Tris(benzyloxy)-N-(1-isobutyl-1H-benzo[d]imidazol-2-yl)benzamide* **26**

Compound **26** was prepared from **18** (0.33 g, 0.8 mmol), **20** (0.17 g, 0.9 mmol), EDC.HCl (0.28 g, 1.4 mmol), HOBt (0.01 g, 0.09 mmol) and DIPEA (0.2 mL, 1.5 mmol) to yield 0.24 g (53%) of pink powder. m.p. 241–245 °C; ^1^H NMR (600 MHz, DMSO-*d_6_*): δ/ppm = 12.67 (s, 1H, NH_amide_), 7.63 (s, 1H, H_arom_), 7.51 (t, 2H, *J* = 6.89 Hz, H_arom_), 7.48 (d, 4H, *J* = 7.28 Hz, H_arom_), 7.43–7.39 (m, 6H, H_arom_), 7.34 (t, 2H, *J* = 7.30 Hz, H_arom_), 7.32–7.29 (m, 3H, H_arom_), 7.24 (td, 1H, *J_1_* = 1.20 Hz, *J_2_* = 7.67 Hz, H_arom_), 7.21 (td, 1H, *J*_1_ = 1.08 Hz, *J*_2_ = 7.69 Hz, H_arom_), 5.23 (s, 4H, CH_2_), 5.07 (s, 2H, CH_2_), 4.04 (d, 2H, *J* = 7.30 Hz, CH_2_), 2.36–2.28 (m, 1H, CH), 0.95 (t, 6H, *J* = 6.66 Hz, CH_3_); ^13^C NMR (151 MHz, DMSO-*d*_6_): δ/ppm = 171.9, 151.7, 150.9, 139.0, 137.0, 136.5, 133.1, 129.1, 128.1, 127.6, 127.6, 127.5, 127.2, 127.0, 126.8, 122.0, 121.8, 111.3, 109.2, 107.0, 69.4, 27.1, 19.4; Anal. Calcd. for C_39_H_37_N_3_O_4_: C, 76.57; H, 6.10; N, 6.87. Found: C, 76.48; H, 6.15; N, 6.91%.

*3,4,5-Tris(benzyloxy)-N-(1-phenyl-1H-benzo[d]imidazol-2-yl)benzamide* **27**

Compound **27** was prepared from **18** (0.33 g, 0.8 mmol), **21** (0.19 g, 0.9 mmol), EDC.HCl (0.28 g, 1.4 mmol), HOBt (0.01 g, 0.09 mmol) and DIPEA (0.2 mL, 1.5 mmol) to yield 0.29 g (61%) of pink powder. m.p. 250–255 °C; ^1^H NMR (600 MHz, DMSO-*d_6_*): δ/ppm = 12.87 (s, 1H, NH_amide_), 7.48–7.22 (m, 26H, H_arom_), 5.09 (s, 4H, CH_2_), 5.02 (s, 2H, CH_2_); ^13^C NMR (151 MHz, DMSO-*d*_6_): δ/ppm = 151.9, 138.0, 137.4, 130.6, 129.7, 128.9, 128.9, 128.7, 128.6, 128.3, 128.0, 127.1, 123.6, 121.7, 119.2, 115.6, 110.1, 108.1, 74.6, 70.3; Anal. Calcd. for C_41_H_33_N_3_O_4_: C, 77.95; H, 5.27; N, 6.65. Found: C, 77.89; H, 5.22; N, 6.59%.

*2,4-Bis(benzyloxy)-N-(5-cyano-1-methyl-1H-benzo[d]imidazol-2-yl)benzamide* **28**

Compound **28** was prepared from **17** (0.08 g, 0.3 mmol), **22** (0.05 g, 0.3 mmol), EDC.HCl (0.08 g, 0.4 mmol), HOBt (0.004 g, 0.03 mmol) and DIPEA (0.07 mL, 0.5 mmol) to yield 0.06 g (43%) of white powder. m.p. 203–205 °C; ^1^H NMR (600 MHz, DMSO-*d_6_*): δ/ppm = 10.60 (s, 1H, NH_amide_), 8.10 (s, 1H, H_arom_), 7.78 (d, 1H, *J* = 8.57 Hz, H_arom_), 7.71 (d, 1H, *J* = 8.38 Hz, H_arom_), 7.65 (dd, 1H, *J*_1_ = 1.30 Hz, *J*_2_ = 8.38 Hz, H_arom_), 7.51 (d, 2H, *J* = 7.28 Hz, H_arom_), 7.47 (d, 2H, *J* = 7.29 Hz, H_arom_), 7.43-7.31 (m, 7H, H_arom_), 6.93 (d, 1H, *J* = 2.00 Hz, H_arom_), 6.79 (dd, 1H, *J*_1_ = 2.18 Hz, *J*_2_ = 8.56 Hz, H_arom_), 5.31 (s, 2H, CH_2_), 5.21 (s, 2H, CH_2_), 3.53 (s, 3H, CH_3_); ^13^C NMR (151 MHz, DMSO-*d*_6_): δ/ppm = 156.7, 142.3, 137.8, 127.9, 127.9, 127.8, 127.8, 127.3, 127.2, 126.4, 121.8, 120.1, 117.1, 107.7, 101.4, 28.0; Anal. Calcd. for C_30_H_24_N_4_O_3_: C, 73.76; H, 4.95; N, 11.47. Found: C, 73.69; H, 4.90; N, 11.52%.

*3,4,5-Tris(benzyloxy)-N-(5-cyano-1-methyl-1H-benzo[d]imidazol-2-yl)benzamide* **29**

Compound **29** was prepared from **18** (0.33 g, 0.8 mmol), **22** (0.15 g, 0.9 mmol), EDC.HCl (0.27 g, 1.4 mmol), HOBt (0.01 g, 0.08 mmol) and DIPEA (0.2 mL, 0.7 mmol) to yield 0.17 g (38%) of pink powder. m.p. 261–265 °C; ^1^H NMR (400 MHz, DMSO-*d_6_*): δ/ppm = 12.86 (s, 1H, NH_amide_), 7.80 (s, 1H, H_arom_), 7.71 (dd, 1H, *J*_1_ = 1.30 Hz, *J*_2_ = 8.28 Hz, H_arom_), 7.66–7.62 (m, 2H, H_arom_), 7.50 (d, 4H, *J* = 7.09 Hz, H_arom_), 7.45–7.26 (m, 12H, H_arom_), 5.23 (s, 2H, CH_2_), 5.08 (s, 2H, CH_2_), 3.71 (s, 3H, CH_3_); ^13^C NMR (151 MHz, DMSO-*d*_6_): δ/ppm = 151.4, 151.1, 136.9, 136.6, 127.8, 127.8, 127.6, 127.5, 127.2, 126.9, 118.9, 107.3, 103.5, 73.6, 73.6, 69.7, 69.6; Anal. Calcd. for C_37_H_30_N_4_O_4_: C, 74.73; H, 5.09; N, 9.42. Found: C, 74.68; H, 5.12; N, 9.49%.

*3,4,5-Tris(benzyloxy)-N-(5-cyano-1-hexyl-1H-benzo[d]imidazol-2-yl)benzamide* **30**

Compound **30** was prepared from **18** (0.39 g, 0.9 mmol), **23** (0.20 g, 0.8 mmol), EDC.HCl (0.19 g, 1.4 mmol), HOBt (0.11 g, 0.8 mmol) and DIPEA (0.3 mL, 1.7 mmol) to yield 0.31 g (56%) of pink powder. m.p. 215–218 °C; ^1^H NMR (600 MHz, DMSO-*d_6_*): δ/ppm = 12.88 (s, 1H, NH_amide_), 7.48–7.29 (m, 20H, H_arom_), 5.22 (s, 4H, CH_2_), 5.07 (s, 2H, CH_2_); 4.24 (t, 2H, *J* = 6.80 Hz, CH_2_), 1.81–1.75 (m, 2H, CH_2_), 1.35–1.27 (m, 6H, CH_2_), 0.76 (t, 3H, *J* = 7.30 Hz, CH_3_); ^13^C NMR (151 MHz, DMSO-*d*_6_): δ/ppm = 151.0, 136.9, 136.5, 127.8, 127.8, 127.6, 127.6, 127.5, 127.3, 127.2, 127.0, 126.9, 126.9, 107.2, 103.5, 73.6, 69.5, 30.2, 25.2, 21.4, 13.1; Anal. Calcd. for C_42_H_40_N_4_O_4_: C, 75.88; H, 6.06; N, 8.43. Found: C, 75.81; H, 6.15; N, 8.36%.

### 4.6. General Method for Preparation of Compounds ***31**–**37***

Compounds **31**–**37** were prepared using microwave irradiation from the benzyl-substituted derivatives **24**–**30**, ammonium formate and 10% Pd/C (0.01 g) in methanol (10 mL). The reaction mixture was irradiated for 30 min at 60 °C with a power of 300 W. The solution was filtered through Celite to remove the catalyst, and the ethanol was removed under reduced pressure. The resulting solid was triturated with a small amount of methanol and collected by filtration.

*N-(1H-benzo[d]imidazol-2-yl)-2,4-dihydroxybenzamide* **31**

Compound **31** was prepared from **24** (0.20 g, 0.4 mmol), ammonium formate (0.61 g, 9.8 mmol) and 10% Pd/C (0.01 g) in methanol (20 mL) to yield 0.03 g (21%) of white powder. m.p. 212–214 °C; ^1^H NMR (300 MHz, DMSO-*d_6_*): δ/ppm = 12.70 (s, 2H, OH), 9.90 (s, 1H, H_arom_), 7.84 (d, 1H, *J* = 8.57 Hz, H_arom_), 7.47–7.42 (m, 2H, H_arom_), 7.25–7.18 (m, 2H, H_arom_), 6.31 (dd, 1H, *J*_1_ = 2.22 Hz, *J*_2_ = 8.55 Hz, H_arom_), 6.23 (d, 1H, *J* = 2.07 Hz, H_arom_); ^13^C NMR (151 MHz, DMSO-*d*_6_,): δ/ppm = 163.1, 162.7, 137.3, 132.0, 128.9, 128.2, 123.1, 112.2, 107.4, 102.9; MS (ESI): *m*/*z* = 370,01 ([M+1]^+^); Anal. Calcd. for C_14_H_11_N_3_O_3_: C, 62.45; H, 4.12; N, 15.61. Found: C, 62.55; H, 4.06; N, 15.74%.

*N-(1H-benzo[d]imidazol-2-yl)-3,4,5-trihydroxybenzamide* **32**

Compound **32** was prepared from **25** (0.06 g, 0.1 mmol), ammonium formate (0.14 g, 2.3 mmol) and 10% Pd/C (0.01 g) in methanol (12 mL) to yield 0.03 g (99%) of light brown powder. m.p. 230–234 °C; ^1^H NMR (600 MHz, DMSO-*d_6_*): δ/ppm = 8.40 (s, 2H, H_arom_), 7.46–7.43 (m, 2H, H_arom_), 7.12 (s, 1H, OH), 7.11–7.08 (m, 2H, H_arom_); ^13^C NMR (151 MHz, DMSO-*d*_6_,): δ/ppm = 166.8, 165.7, 146.2, 146.1, 139.1, 121.5, 119.7, 119.6, 109.0, 108.4; MS (ESI): *m*/*z* = 285.99 ([M+1]^+^); Anal. Calcd. for C_14_H_11_N_3_O_4_: C, 58.95; H, 3.89; N, 14.73. Found: C, 58.89; H, 3.91; N, 14.67%.

*3,4,5-Trihydroxy-N-(1-isobutyl-1H-benzo[d]imidazol-2-yl)benzamide* **33**

Compound **33** was prepared from **26** (0.20 g, 0.3 mmol), ammonium formate (0.37 g, 5.9 mmol) and 10% Pd/C (0.02 g) in methanol (15 mL) to yield 0.04 g (37%) of white powder. m.p. 291–293 °C; ^1^H NMR (600 MHz, DMSO-*d_6_*): δ/ppm = 12.60 (s, 1H, NH_amide_), 8.93 (s, 2H, OH), 8.53 (s, 1H, OH), 7.49 (d, 1H, *J* = 7.58 Hz, H_arom_), 7.46 (d, 1H, *J* = 7.70 Hz, H_arom_), 7.25 (s, 2H, H_arom_), 7.23–7.15 (m, 2H, H_arom_), 4.03 (d, 2H, *J* = 7.31 Hz, CH_2_), 2.35–2.27 (m, 1H, CH), 0.97 (d, 6H, *J* = 6.68 Hz, CH_3_); ^13^C NMR (151 MHz, DMSO-*d*_6_,): δ/ppm = 173.3, 151.9, 144.3, 135.9, 129.1, 128.2, 128.1, 121.7, 121.5, 111.0, 108.9, 107.8, 47.9, 27.0, 19.4; MS (ESI): *m*/*z* = 341.89 ([M+1]^+^); Anal. Calcd. for C_18_H_19_N_3_O_4_: C, 63.33; H, 5.61; N, 12.31. Found: C, 63.27; H, 5.69; N, 12.24%.

*3,4,5-Trihydroxy-N-(1-phenyl-1H-benzo[d]imidazol-2-yl)benzamide* **34**

Compound **34** was prepared from **27** (0.07 g, 0.1 mmol), ammonium formate (0.04 g, 5.9 mmol) and 10% Pd/C (0.007 g) in methanol (10 mL) to yield 0.02 g (50%) of white powder. m.p. 278–281 °C; ^1^H NMR (600 MHz, DMSO-*d_6_*): δ/ppm = 8.35 (s, 1H, H_arom_), 7.69–7.59 (m, 5H, H_arom_), 7.55–7.51 (m, 1H, H_arom_), 7.28–7.24 (m, 1H, H_arom_), 7.23-7.13 (m, 2H, H_arom_), 7.00 (s, 1H, H_arom_), 3.77 (bs, 3H, OH); ^13^C NMR (151 MHz, DMSO-*d*_6_,): δ/ppm = 130.6, 129.9, 128.7, 127.1, 123.3, 121.6, 119.2, 115.6, 108.9; MS (ESI): *m*/*z* = 362.12 ([M+1]^+^); Anal. Calcd. for C_20_H_15_N_3_O_4_: C, 66.48; H, 4.18; N, 11.63. Found: C, 66.54; H, 4.26; N, 11.67%.

*N-(5-cyano-1-methyl-1H-benzo[d]imidazol-2-yl)-2,4-dihydroxybenzamide* **35**

Compound **35** was prepared from **28** (0.05 g, 0.1 mmol), ammonium formate (0.15 g, 146.4 mmol) and 10% Pd/C (0.01 g) in methanol (10 mL) to yield 0.01 g (13%) of light brown powder. m.p. 273–275 °C; ^1^H NMR (600 MHz, DMSO-*d_6_*): δ/ppm = 13.66 (s, 1H, OH), 10.01 (s, 1H, NH_amide_), 7.93 (d, 1H, *J* = 8.56 Hz, H_arom_), 7.84 (s, 1H, H_arom_), 7.73 (s, 2H, H_arom_), 6.37–6.29 (m, 1H, H_arom_), 6.23 (s, 1H, H_arom_), 3.69 (s, 3H, CH_3_); ^13^C NMR (151 MHz, DMSO-*d*_6_,): δ/ppm = 162.2, 151.3, 132.8, 131.4, 128.5, 126.6, 118.7, 114.9, 110.7, 110.4, 106.6, 103.9, 101.7, 28.3; MS (ESI): *m*/*z* = 309.07 ([M+1]^+^); Anal. Calcd. for C_16_H_12_N_4_O_3_: C, 62.33; H, 3.92; N, 18.17. Found: C, 62.26; H, 4.01; N, 18.09%

*N-(5-cyano-1-methyl-1H-benzo[d]imidazol-2-yl)-3,4,5-trihydroxybenzamide* **36**

Compound **36** was prepared from **29** (0.10 g, 0.1 mmol), ammonium formate (0.10 g, 106.1 mmol) and 10% Pd/C (0.01 g) in methanol (10 mL) to yield 0.01 g (24%) of light brown powder. m.p. 245–251 °C; ^1^H NMR (600 MHz, DMSO-*d_6_*): δ/ppm = 12.78 (s, 1H, OH), 8.98 (s, 1H, H_arom_), 7.79–7.59 (m, 3H, H_arom_), 7.29 (s, 2H, H_arom_), 3.69 (s, 3H, CH_3_); ^13^C NMR (151 MHz, DMSO-*d*_6_,): δ/ppm = 173.8, 152.7, 144.4, 136.3, 133.1, 128.7, 127.7, 127.4, 126.3, 114.0, 109.6, 108.0, 103.1, 27.8; MS (ESI): *m*/*z* = 325.06 ([M+1]^+^); Anal. Calcd. for C_16_H_12_N_4_O_4_: C, 59.26; H, 3.73; N, 17.28. Found: C, 59.20; H, 3.77; N, 17.36%.

*N-(5-cyano-1-hexyl-1H-benzo[d]imidazol-2-yl)-3,4,5-trihydroxybenzamide* **37**

Compound **37** was prepared from **30** (0.20 g, 0.3 mmol), ammonium formate (0.13 g, 2.1 mmol) and 10% Pd/C (0.02 g) in methanol (10 mL) to yield 0.02 g (15%) of light brown powder. m.p. 290–294 °C; ^1^H NMR (600 MHz, DMSO-*d_6_*): δ/ppm = 12.80 (s, 1H, NH_amide_), 8.96 (s, 2H, OH), 8.63 (s, 1H, OH), 7.78 (s, 1H, H_arom_), 7.68–7.63 (m, 2H, H_arom_), 7.26 (s, 2H, H_arom_), 4.24 (t, 2H, *J* = 7.02 Hz, CH_2_), 1.82–1.75 (m, 2H, CH_2_), 1.30–1.18 (m, 6H, CH_2_), 0.82 (t, 3H, *J* = 7.27 Hz, CH_3_); ^13^C NMR (151 MHz, DMSO-*d*_6_,): δ/ppm = 173.8, 152.5, 144.8, 144.4, 136.3, 132.4, 128.7, 127.5, 126.3, 118.9, 116.9, 114.2, 109.7, 108.0, 106.9, 103.1, 30.2, 27.0, 25.1, 21.4, 13.2; MS (ESI): *m*/*z* = 395.20 ([M+1]^+^); Anal. Calcd. for C_21_H_22_N_4_O_4_: C, 63.95; H, 5.62; N, 14.20. Found: C, 63.90; H, 5.66; N, 14.12%.

### 4.7. Antiproliferative Activity

The experiments were performed on four human cell lines, including HCT 116 (colon carcinoma), H 460 (lung carcinoma), MCF-7 (breast carcinoma) and HEK 293 (human embryonic kidney cells), in line with previously published experimental procedures [10,25]. Briefly, the cells were grown in DMEM medium with the addition of 10% fetal bovine serum (FBS), 2 mM of L-glutamine, 100 U/mL of penicillin and 100 µg/mL of streptomycin and cultured as monolayers at 37 °C in a humidified atmosphere with 5% CO_2_. Cells were seeded at 2 × 103 cells/well in standard 96-well microtiter plates and left to attach for 24 h. The next day, a test compound was added in five serial 10-fold dilutions. The rate of cell growth was evaluated after 72 h of incubation with MTT assays. The obtained results are expressed as IC_50_ values, calculated from the concentration–response curves using linear regression analysis by fitting the test concentrations that give PG values above and below the reference value (i.e., 50%). Each test was performed in quadruplicate in at least two individual experiments.

### 4.8. Antioxidative Activity Assay in Cells

For the antioxidative activity assay, 2.5 × 10^4^ cells were seeded into 96-well microtiter plates and left to attach for 24 h. The next day, cells were washed with PBS and incubated in FBS-free DMEM medium with 25 µM of DCFH-DA fluorescence dye [35]. After 45 min of incubation, the medium was discarded, and the cells were washed with PBS.

After the washing step, the cells were incubated with 100 µM of *tert*-butyl hydroperoxide (TBHP) alone or in combination with antioxidative agents (50 mM of N-Acetyl-L-cysteine-NAC or 10 µM of the tested compounds) in PBS for 1 h at 37 °C. DCFH-DA fluorescence was recorded on a microplate fluorimeter reader (Tecan) with an excitation beam of 485 nm, while the emitted fluorescence was collected at 535 nm. All tests were presented as the means of two independent measurements carried out in triplicate. One-way ANOVA with Tukey’s post hoc test was used for statistical analysis: *—*p* < 0.05; **—*p* < 0.01; ***—*p* < 0.001.

### 4.9. Antioxidative Activity

Determination of the reducing activity of the stable radical 1,1-diphenyl-picrylhydrazyl (DPPH):

The reducing activity of the investigated systems was measured by the DPPH method according to previously described procedures with modifications to ensure their use on a 96-well microplate. Briefly, equal volumes of various concentrations of the tested molecules (dissolved in DMSO) were added to a solution of DPPH (final concentration of 50 µM in absolute ethanol). Ethanol and DMSO were used as control solutions in line with earlier reports [36]. The radical scavenging activity was calculated using the following formula:

[(A_517_ control − A_517_ compound)/A_517_ control] × 100.

The IC_50_ values were calculated from dose–response curves using nonlinear regression analysis with the GraphPad Prism 8 Ink. program. All measurements were carried out in triplicate.

#### 4.9.1. Determination of Ferric Reducing/Antioxidant Power (FRAP Assay)

The FRAP method was carried out according to previously described procedures with some modifications to make it compatible with an assay on a 96-well microplate [37]. All results were expressed as Fe^2+^ equivalents (Fe^2+^ µmol). Ferrous sulphate (FeSO_4_ × 7H_2_O) was used to develop a standard curve of 20–2000 µmol/l for the FRAP test. All tests were carried out in triplicate, and the results were averaged and are presented in Table 1.

#### 4.9.2. ABTS Radical Scavenging Assay

The total antioxidant activity (TEAC) method was adapted for use with a microplate reader. Initially, ABTS•+ (2,2′-azino-bis(3-ethylbenzothiazoline-6-sulfonic acid) radical cation) was generated by combining an ABTS stock solution (7 mM in water) with 2.45 mM of potassium persulfate, and it was allowed to stand for 12–16 h at room temperature in darkness until it achieved a stable oxidative state. On the day of analysis, the ABTS•+ solution was diluted with PBS (pH7.4) to attain an absorbance of 0.700 ± 0.01 at 734 nm. This radical state remained stable for over two days when stored in the dark at room temperature. The standards and solutions of the tested compounds (10 µL) were mixed with the working ABTS•+ solution (200 µL) in microplate wells and incubated at room temperature for 5 min. The reduction in absorbance at 734 nm was measured using a µQuant (Biotec Inc., Auburn, CA, USA) microplate reader. The percentage scavenging of the test samples at each concentration was calculated using the following formula:

[(Abs_control_ − Abs_compound_)/Abs_control_] × 100

The IC_50_ values for each compound were calculated from dose–response curves. All measurements where carried out in triplicate with a linear regression analysis [38].

### 4.10. Antibacterial Activity In Vitro

#### 4.10.1. Materials

In addition to the synthesized compounds, the standard antibiotics ampicillin, ceftazidime, ciprofloxacin and meropenem from USP were tested. The selected bacterial strains were Gram-negative *Escherichia coli* (ATCC 25922) and Gram-positive *Staphylococcus aureus* (ATCC 29213) and *S. Pneumoniae* (ATCC 49619). The *Saccharomyces cerevisiae* ATCC 7752 strain was tested as a eukaryotic model organism. Synthesized compounds were prepared as 10 mM DMSO solutions and tested in a final concentration range of 100–0.2 µM [39]. Standard antibiotics were prepared as 5 mg/mL DMSO solutions and tested in a final concentration range of 64–0.125 µg/mL.

#### 4.10.2. Methods

Broth microdilution testing was performed according to CLSI (Clinical Laboratory Standards Institute) guidelines. The MIC (minimal inhibitory concentration) value was defined as the last tested concentration of a compound at which there is no visible growth of bacteria. Inoculums for each microorganism were prepared using the direct colony suspension method, where broth solutions that achieved turbidity equivalent to 0.5 McFarland standards were additionally diluted 100× with cation-adjusted MH media (Becton Dickinson). All test plates were incubated for 16–24 h at 37 °C. MIC values for reference antibiotics against quality control strains were used to confirm the validity of the screen according to the Clinical and Laboratory Standards Institute (CLSI) guidelines: methods for dilution antimicrobial susceptibility tests for bacteria that grow aerobically, M07, 11th edition, 2018, and performance standards for antimicrobial susceptibility testing, M100, 28th edition, 2018.

## Figures and Tables

**Table 1:** Antiproliferative activity in vitro of compounds **7**–**12** and **31**–**37**.

	IC_50_/μM			
Cpd.	Cell Line			
	H460	HCT 116	MCF-7	HEK293
**7**	>100	26.4 ± 4.8	36.3 ± 1.8	- ^a^
**8**	91.8 ± 1.0	>100	52.9 ± 16.0	49.4 ± 3.3
**9**	>100	>100	35.5 ± 5.2	32.3 ± 6.5
**10**	4.4 ± 0.4	3.9 ± 0.4	2.2 ± 0.9	4.4 ± 0.3
**11**	>100	3.7 ± 0.04	1.2 ± 0.3	5.3 ± 0.6
**12**	>100	- ^a^	3.1 ± 2,6	- ^a^
**31**	>100	38.6 ± 15.6	35.1 ± 6.9	24.5 ± 6.4
**32**	>100	>100	39.8 ± 1.4	66.0 ± 1.7
**33**	>100	43.2 ± 18.6	90.8 ± 1.6	77.1 ± 7.9
**34**	>100	>100	49.4 ± 27.3	85.1 ± 8.8
**35**	>100	10.0 ± 1.6	8.7 ± 1.5	33.5 ± 6.5
**36**	48.3 ± 10.5	19.3 ± 6.4	4.8 ± 0.7	25.3 ± 0.7
**37**	47.0 ± 8.7	37.8 ± 6.0	38.2 ± 3.2	37.9 ± 0.4
Doxorubicin	0.04 ± 0.01	0.04 ± 0.01	0.009 ± 0.004	0.02 ± 0.01
Etoposide	2.2 ± 0.4	2.2 ± 0.4	0.6 ± 0.1	0.65 ± 0.02

^a^ Compounds did not show biological activity due to precipitation.

**Table 2:** Antioxidative activities in vitro of compounds **7**–**12** and **31**–**37**.

Cpd.	FRAP mmolFe^2+^/mg cpd.	ABTS IC_50_ mM (* µM)	DPPH IC_50_ mM (* µM)
**7**	387.28 ± 7.56	86 ± 1.20	6.44 ± 0.74
**8**	2461.93 ± 21.42	120 ± 0.42	1.5 ± 0.07
**9**	4036.77 ± 8.82	37.80 ± 2.09	0.75 ± 0.03
**10**	123.61 ± 2.52	9.32 ± 1.73	0.7832 ± 0.00
**11**	422.55 ± 8.82	84 ± 2.34	3.79 ± 0.33
**12**	592.86 ± 13.86	12.49 ± 1.40	0.596 ± 0.02
**31**	89.29 ± 21.92	0.13 ± 0.006	0.14 ± 0.05
**32**	3245.79 ± 16.38	40.09 ± 2.80	1.05 ± 0.08
**33**	4154.35 ± 1.26	66.30 ± 1.90	0.9 ± 0.02
**34**	6.83 ± 1.26	40.20 ± 0.27	1.25 ± 0.07
**35**	278.61 ± 3.28	0.19 ± 0.04	0.716 ± 0.02
**36**	2396.01 ± 35.27	64 ± 0.13	1.41 ± 0.05
**37**	2780.81 ± 15.12	2 ± 0.10	1.02 ± 0.06
BHT	2089.34 ± 55.98	23.12 ± 0.12	25 ± 0.42

**Table 3:** Antibacterial activity in vitro of compounds **7**–**12** and **31**–**37**.

Cpd.	*S. aureus* ATCC 29213	*E. faecalis* ATCC 29212	*E. coli* ATCC 25922	*E. coli* efflux del.	*P. aeruginosa* ATCC 27853	*A. baumannii* ATCC 17978
**7**	>64	16	>64	>64	>64	>64
**8**	>64	8	>64	>64	>64	>64
**9**	64	64	>64	>64	>64	>64
**10**	>64	>64	>64	>64	>64	>64
**11**	>64	32	>64	>64	>64	>64
**12**	>64	32	>64	>64	>64	>64
**31**	>64	32	>64	64	>64	>64
**32**	>64	>64	>64	>64	>64	>64
**33**	32	64	>64	64	>64	>64
**34**	>64	32	>64	>64	>64	>64
**35**	>64	16	>64	64	>64	>64
**36**	32	64	>64	>64	>64	>64
**37**	16	16	>64	16	>64	>64
Ampicillin	0.5	1	2	2	>64	32
Azithromycin	1	8	4	0.5	32	8
Ceftazidime	4	>64	<0.125	<0.125	2	4
Ciprofloxacin	0.25	4	<0.125	<0.125	0.25	<0.125
Gentamicin	0.25	16	0.5	1	2	4
Meropenem	<0.125	8	<0.125	<0.125	1	0.5
Tetracycline	0.25	16	0.5	32	32	2
Tobramycin	0.25	8	1	1	0.5	2

## Data Availability

The raw data supporting the conclusions of this article will be made available by the authors on request.

## Supplementary Materials

The following are available online at https://www.mdpi.com/article/10.3390/molecules29092138/s1: Figures S1–S62: 1H and 13C NMR data of prepared compounds; Figure S63: Reaction scheme for the preparation of compounds **1**–**6**.
