# Supplementary material for: Design, Synthesis and Biological Activity of Novel Methoxy- and Hydroxy-Substituted N-Benzimidazole-Derived Carboxamides
Source: Molecules. 2024 May 4;29(9):2138. doi: 10.3390/molecules29092138 (PMC11085308; doi:10.3390/molecules29092138)
Supplement: Supplementary file 1 [file molecules-29-02138-s001.zip › molecules-2953207-supplementary.pdf]

Design, synthesis and biological activity of novel methoxy and hydroxy substituted *N*- benzimidazole derived carboxamides

Anja Beč[1], Katarina Zlatić[2], Mihailo Banjanac[3], Vedrana Radovanović[3], Kristina Starčević[4], Marijeta Kralj[2] and Marijana Hranjec[1]*

[1] Department of Organic Chemistry, Faculty of Chemical Engineering and Technology, University of Zagreb, Marulićev trg 19, HR-10000 Zagreb, Croatia

[2] Division of Molecular Medicine, Ruđer Bošković Institute, Bijenička cesta 54, HR-10000 Zagreb, Croatia

[3] Pharmacology *in vitro*, Selvita Ltd. Prilaz baruna Filipovića 29, 10000 Zagreb, Croatia

[4] Department of Chemistry and Biochemistry, Faculty of Veterinary Medicine, University of Zagreb, Heinzelova 55, HR-10000 Zagreb, Croatia

Content of SI

1. Figures S1-S62: NMR spectra of prepared compounds
2. Figures S63: Reaction scheme for the preparation of compounds **1–6**

Figure S1. ^{1}H NMR spectrum (DMSO-d_6, 600 MHz) of ***(E)-2-(benzo[d]thiazol-2-yl)-3-phenylacrylonitrile*** ***17***

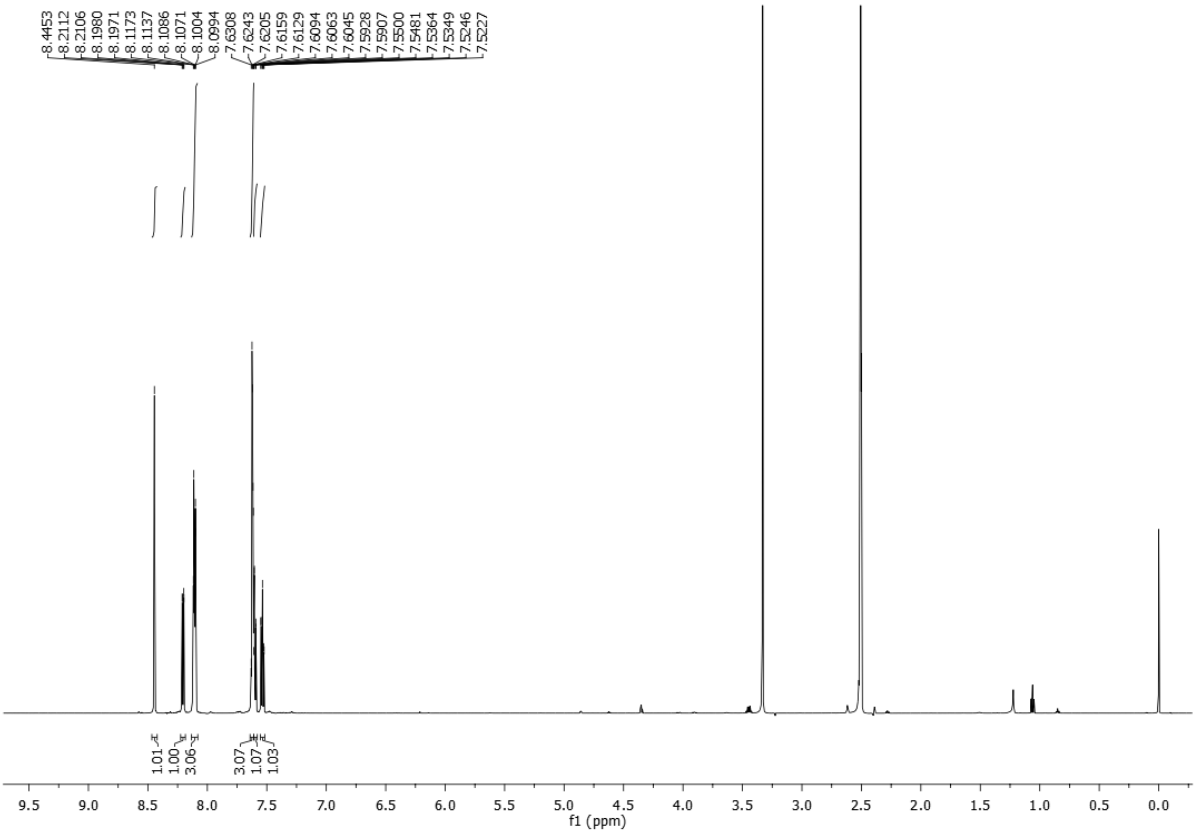

Figure S2. ^{13}C NMR spectrum (DMSO-d_6, 151 MHz) of ***(E)-2-(benzo[d]thiazol-2-yl)-3-phenylacrylonitrile*** ***17***

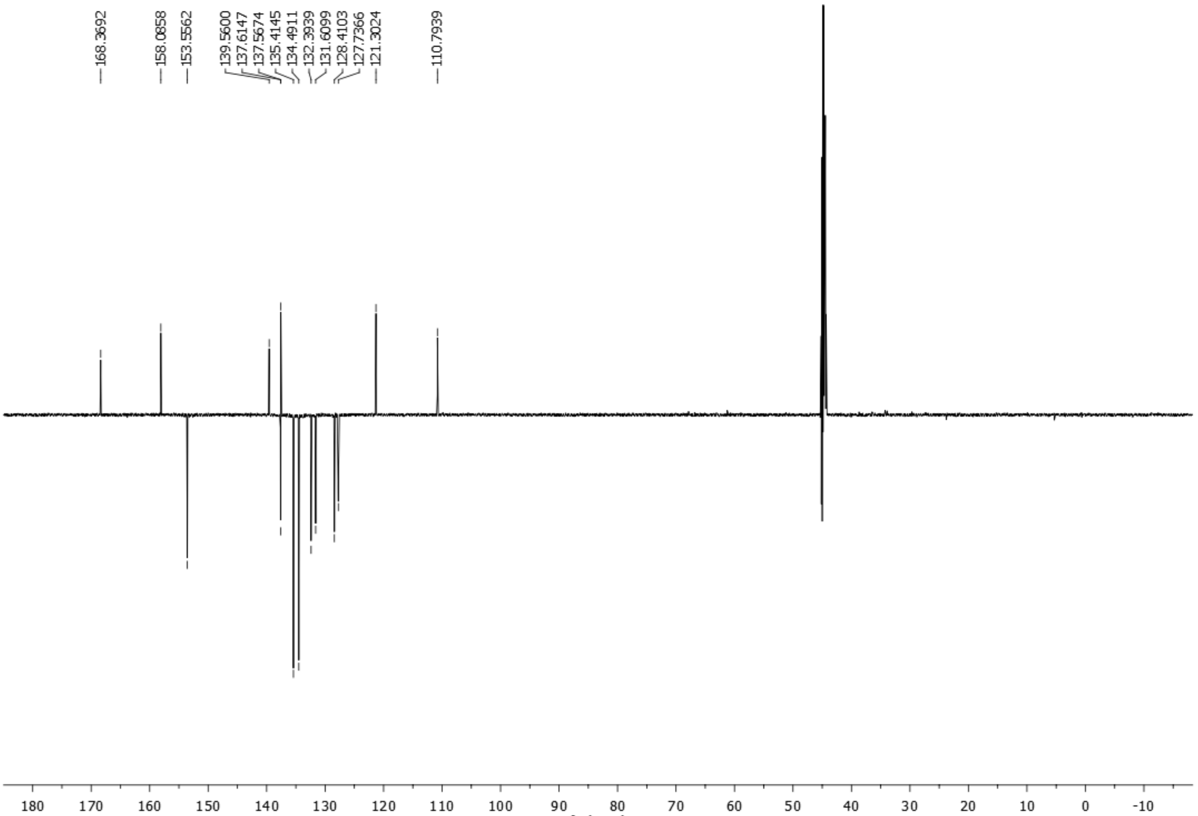

Figure S3. ^{1}H NMR spectrum (DMSO-d_6, 300 MHz) of ***(E)-2-(benzo[d]thiazol-2-yl)-3-(2-methoxyphenyl)acrylonitrile*** ***18***

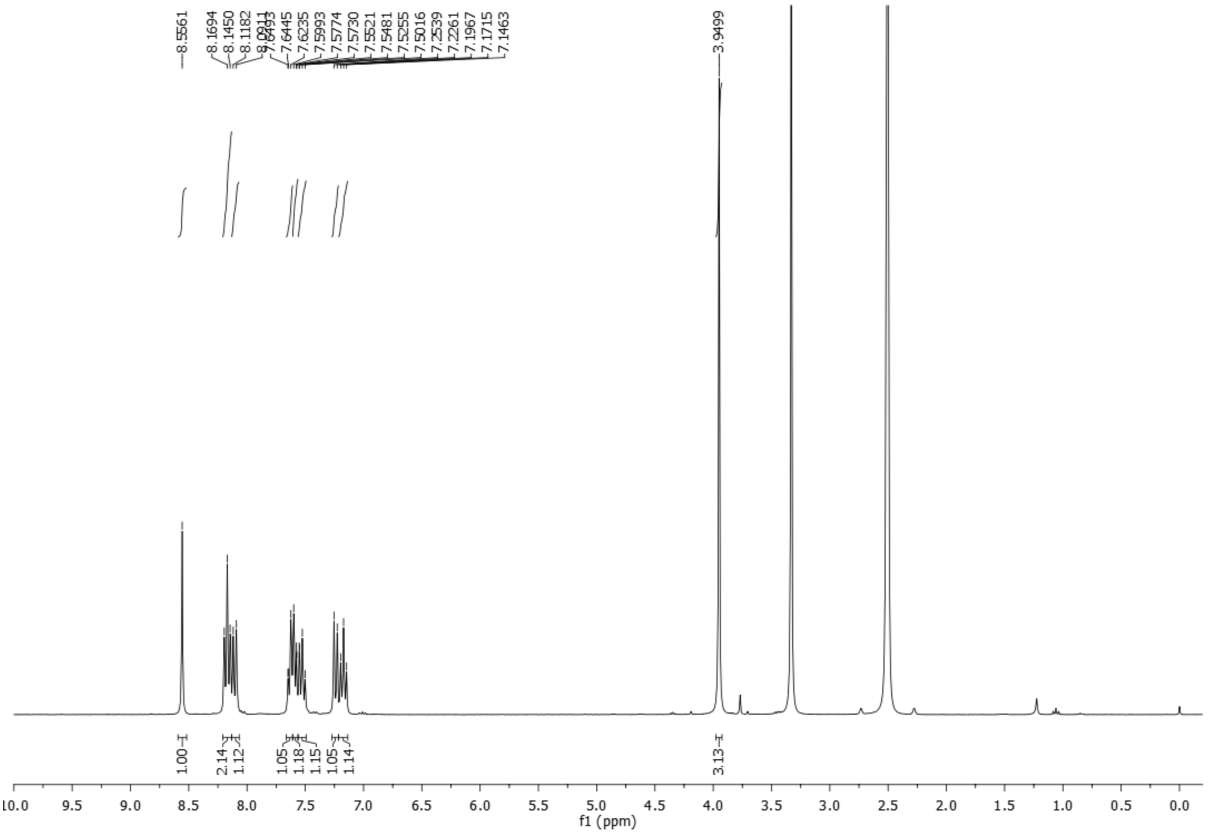

Figure S4. ^{13}C NMR spectrum (DMSO-d_6, 151 MHz) of ***(E)-2-(benzo[d]thiazol-2-yl)-3-(2-methoxyphenyl)acrylonitrile*** ***18***

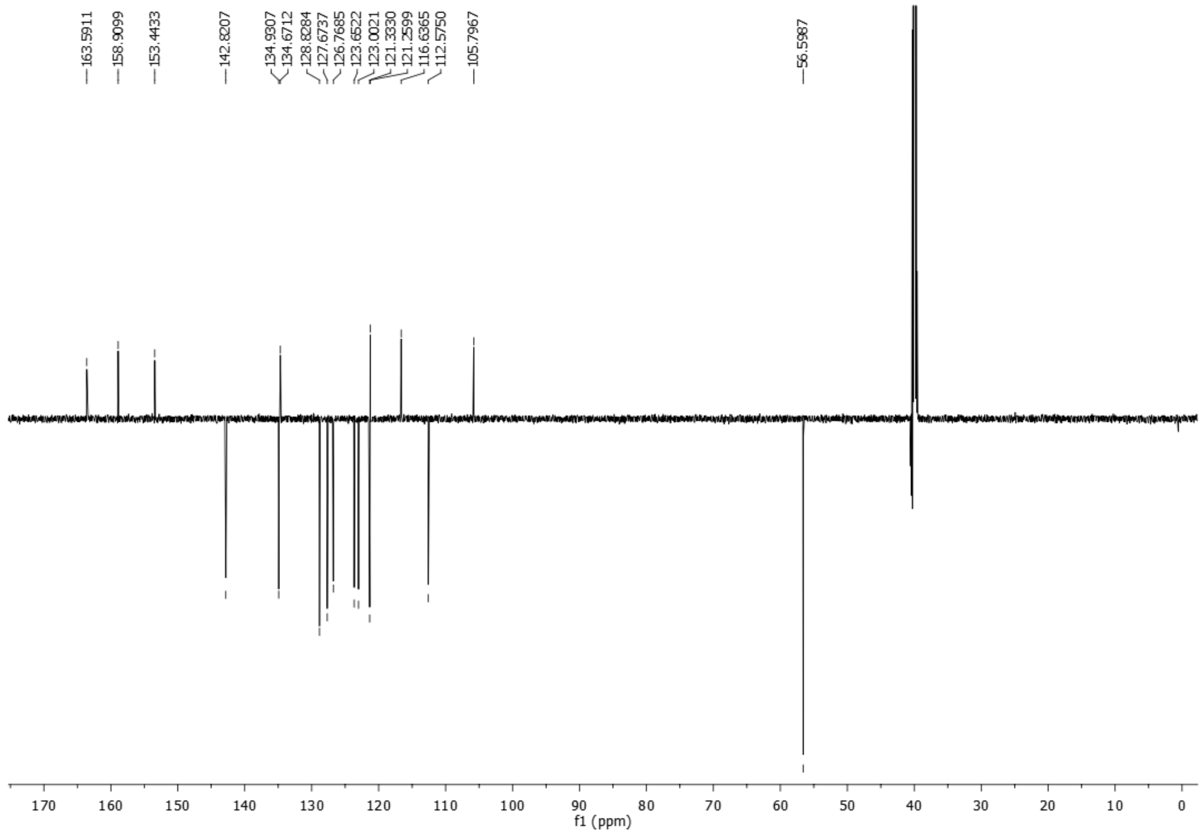

Figure S5. ^{1}H NMR spectrum (DMSO-d_6, 300 MHz) of ***(E)-2-(benzo[d]thiazol-2-yl)-3-(2,4-dimethoxyphenyl)acrylonitrile*** ***19***

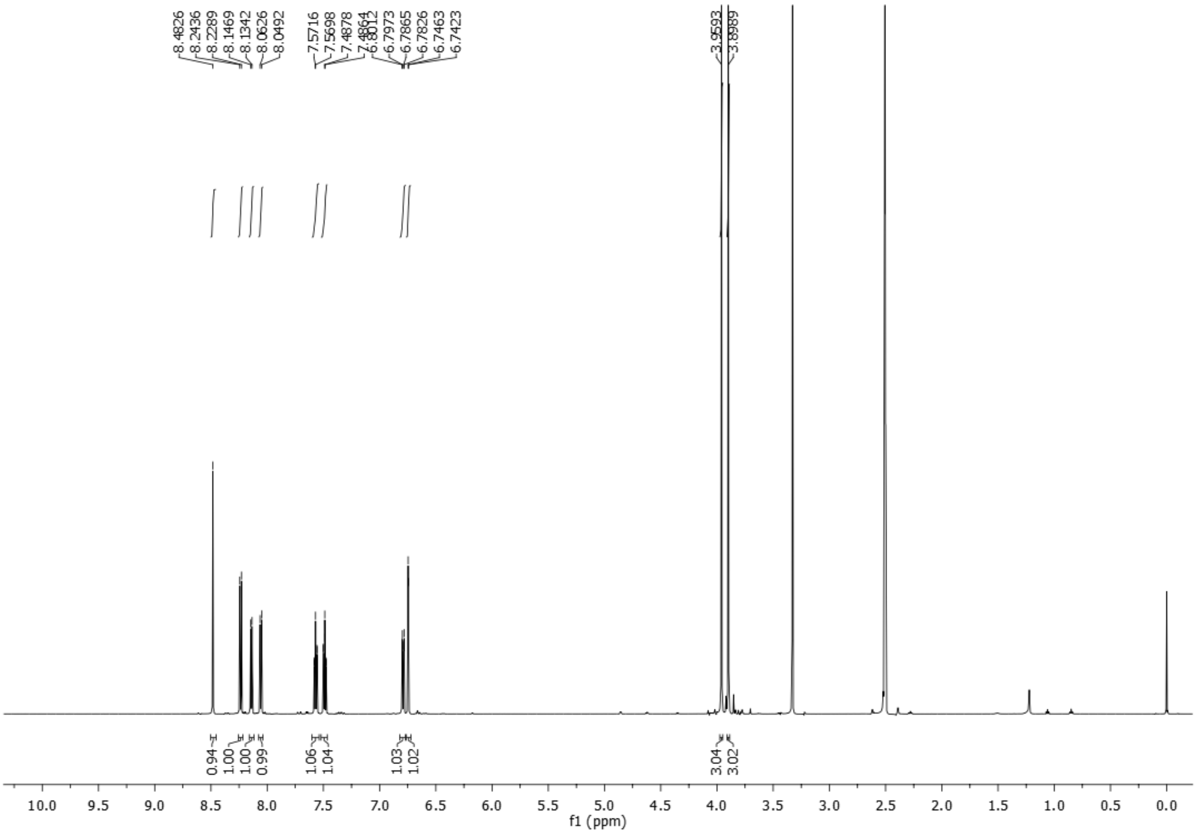

Figure S6. ^{13}C NMR spectrum (DMSO-d_6, 151 MHz) of ***(E)-2-(benzo[d]thiazol-2-yl)-3-(2,4-dimethoxyphenyl)acrylonitrile*** ***19***

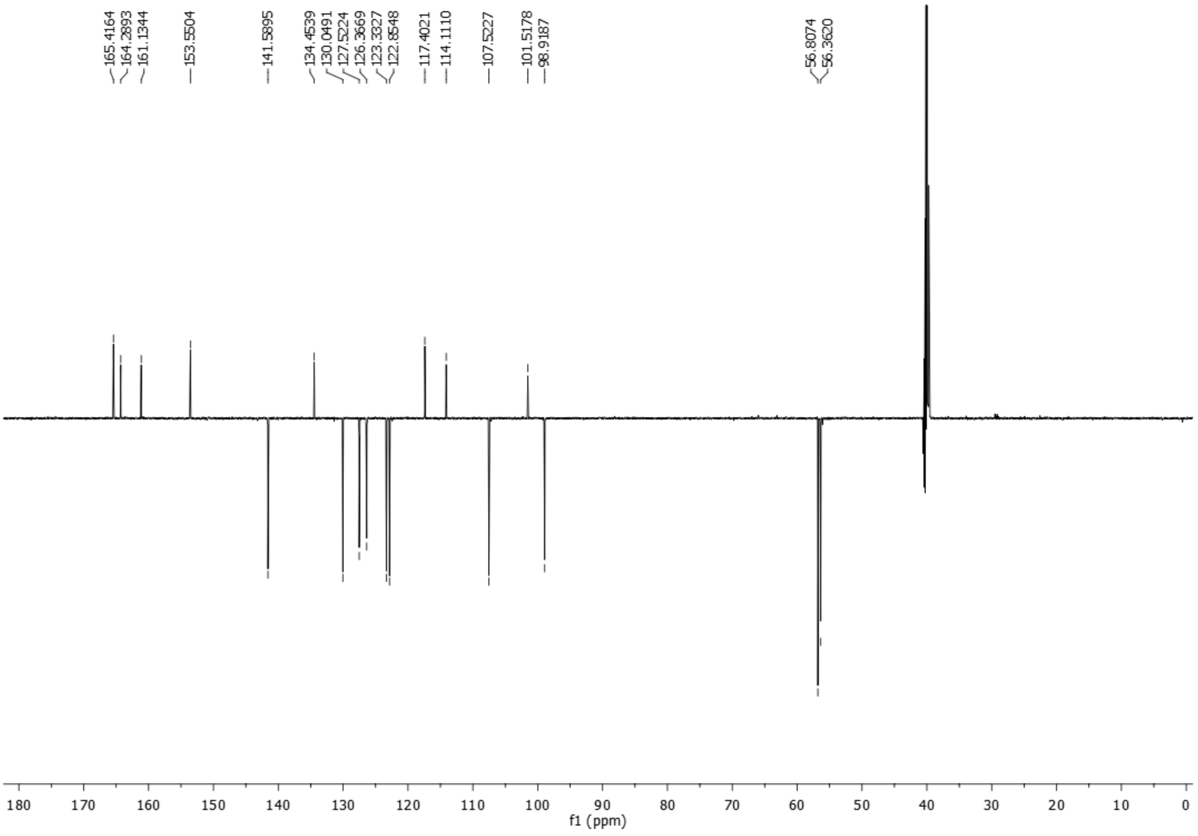

Figure S7. ^{1}H NMR spectrum (DMSO-d_6, 600 MHz) of ***(E)-2-(benzo[d]thiazol-2-yl)-3-(3,4,5-trimethoxyphenyl)acrylonitrile*** ***20***

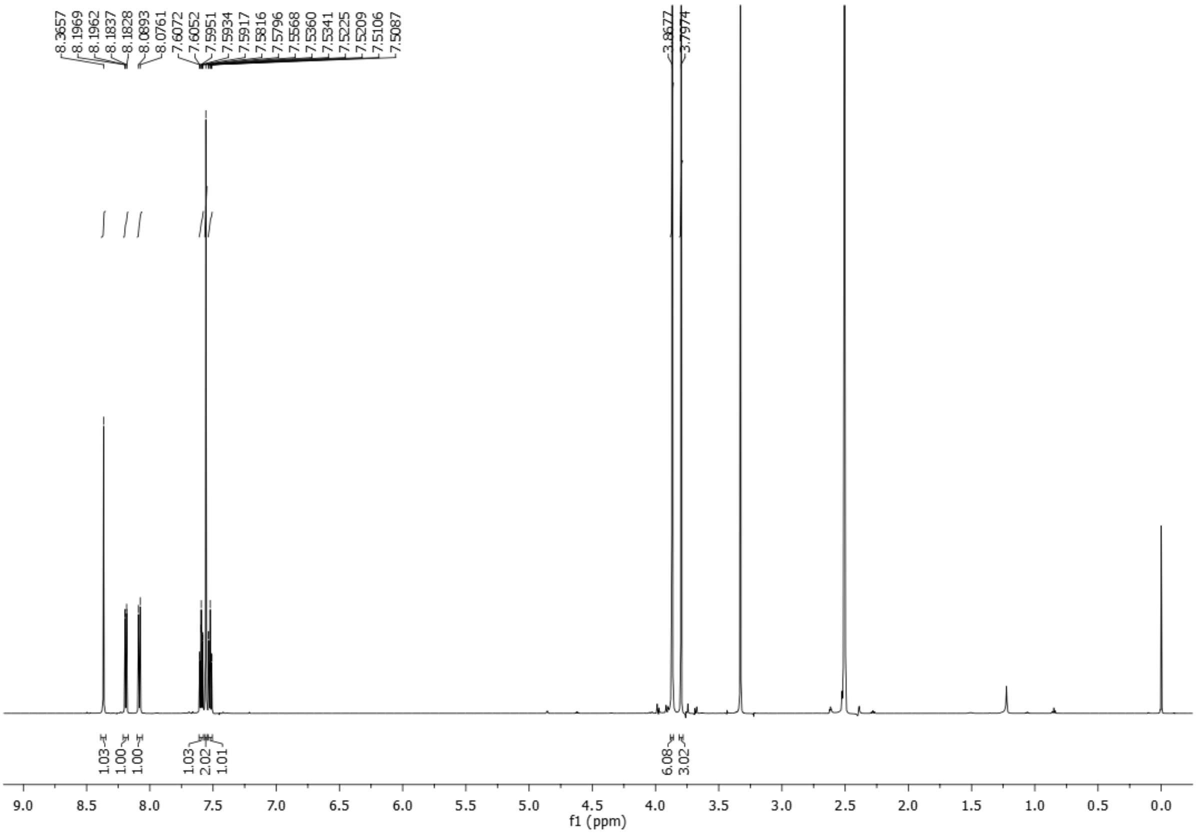

Figure S8. ^{13}C NMR spectrum (DMSO-d_6, 151 MHz) of ***(E)-2-(benzo[d]thiazol-2-yl)-3-(3,4,5-trimethoxyphenyl)acrylonitrile*** ***20***

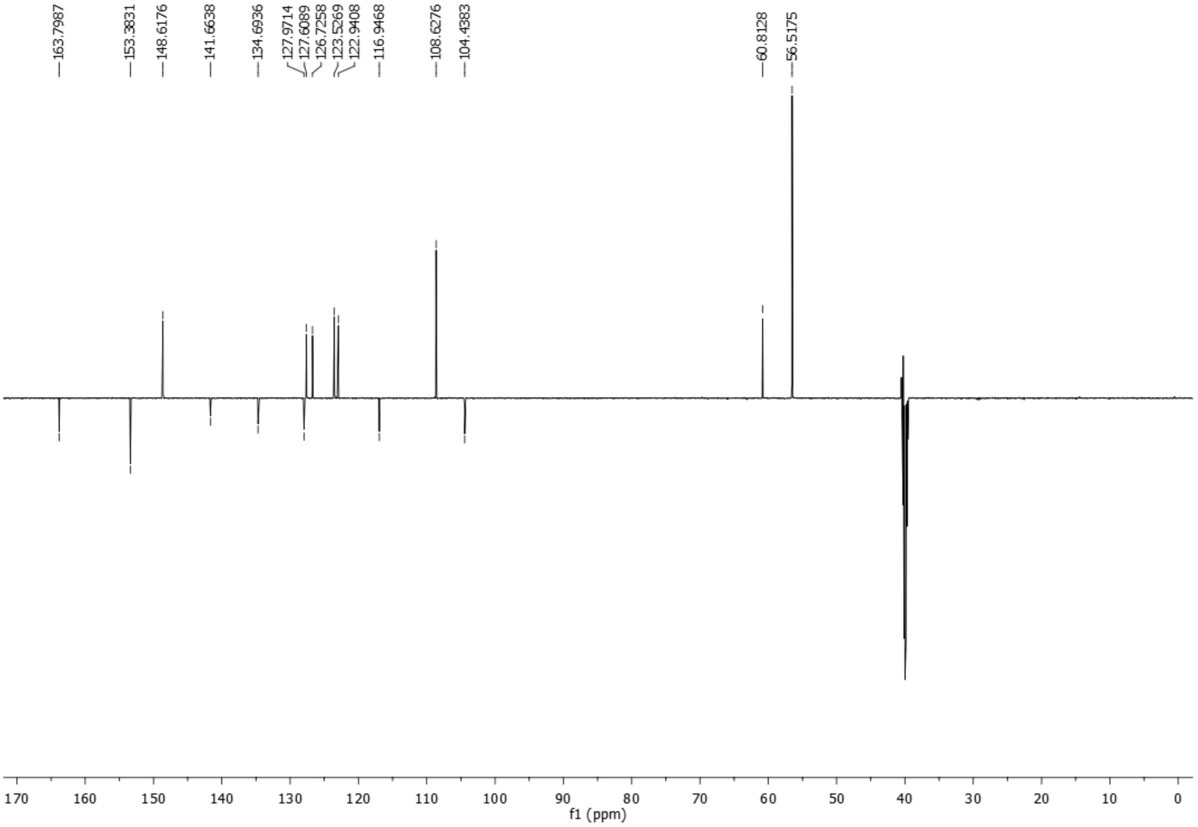

Figure S9. ^{1}H NMR spectrum (DMSO-d_6, 300 MHz) of ***(E)-2-(benzo[d]thiazol-2-yl)-3-(4-hydroxyphenyl)acrylonitrile*** ***21***

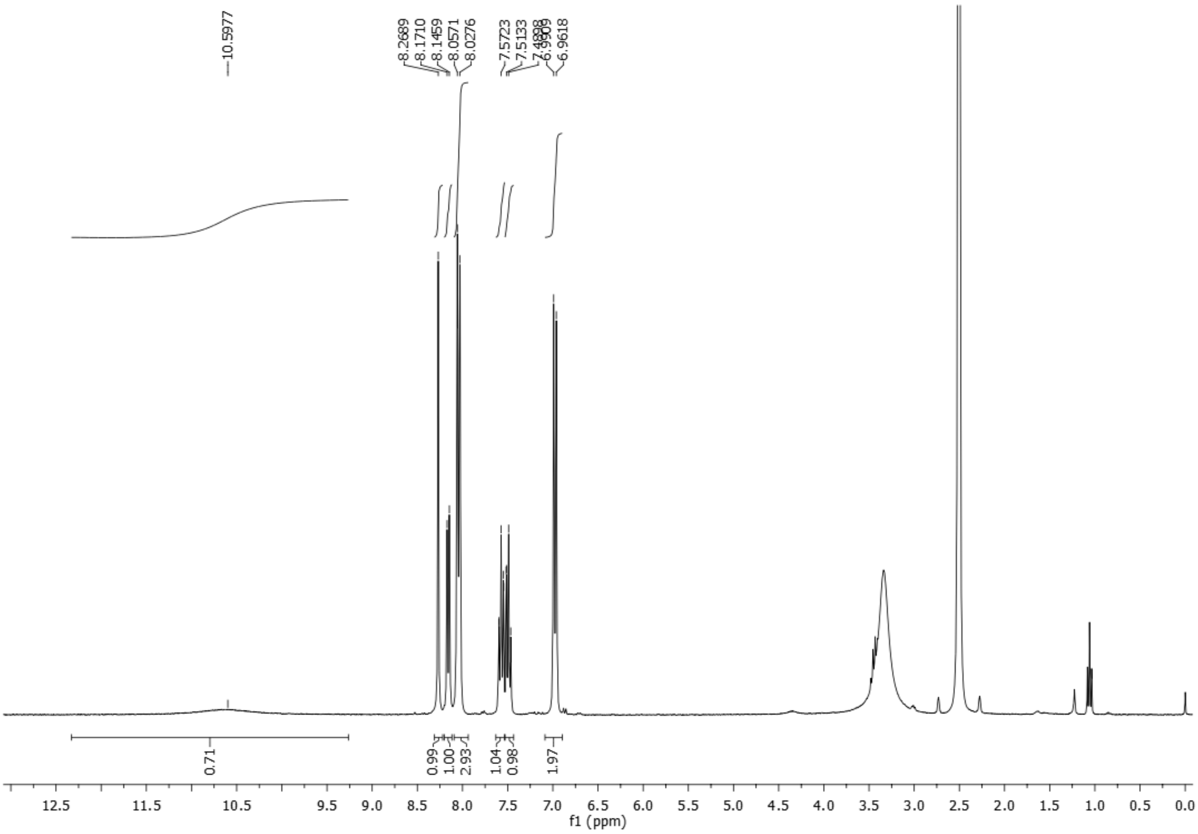

Figure S10. ^{13}C NMR spectrum (DMSO-d_6, 151 MHz) of ***(E)-2-(benzo[d]thiazol-2-yl)-3-(4-hydroxyphenyl)acrylonitrile*** ***21***

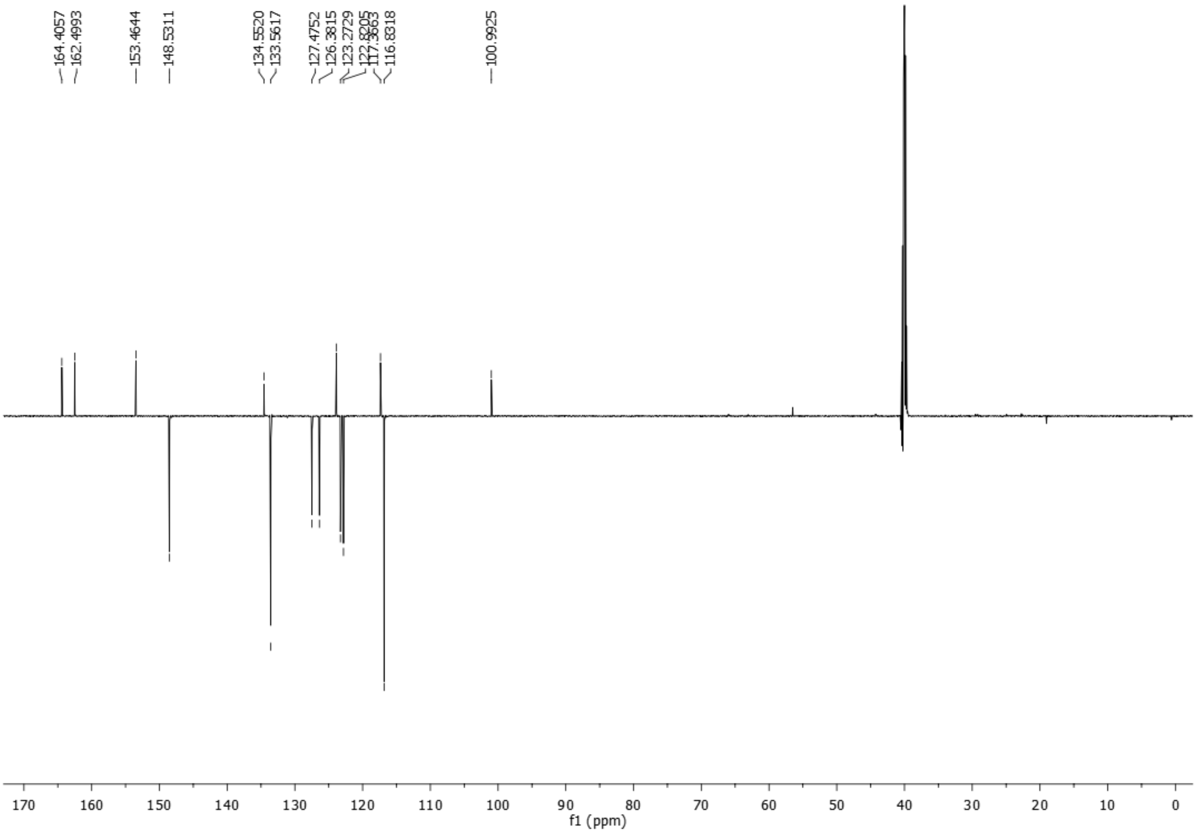

Figure S11. ^{1}H NMR spectrum (DMSO-d_6, 400 MHz) of ***(E)-2-(benzo[d]thiazol-2-yl)-3-(3,4-dihydroxyphenyl)acrylonitrile*** ***22***

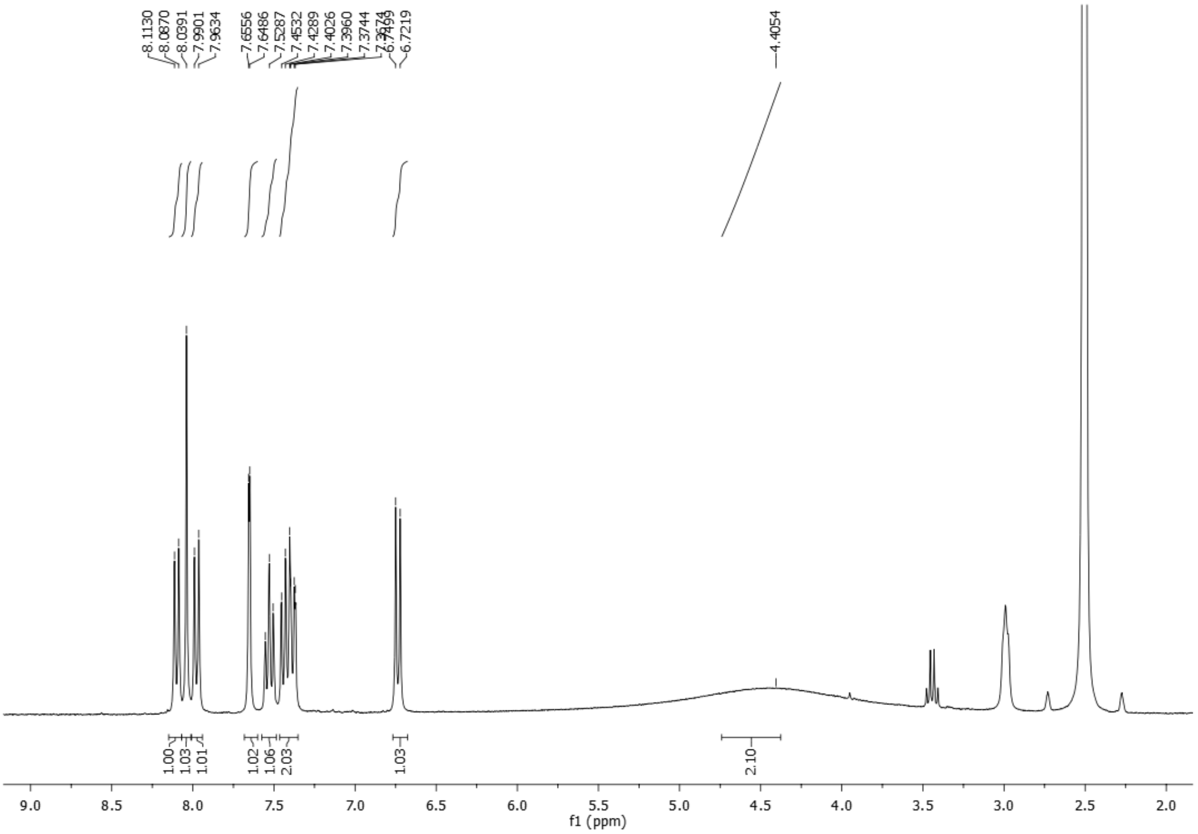

Figure S12. ^{13}C NMR spectrum (DMSO-d_6, 151 MHz) of ***(E)-2-(benzo[d]thiazol-2-yl)-3-(3,4-dihydroxyphenyl)acrylonitrile*** ***22***

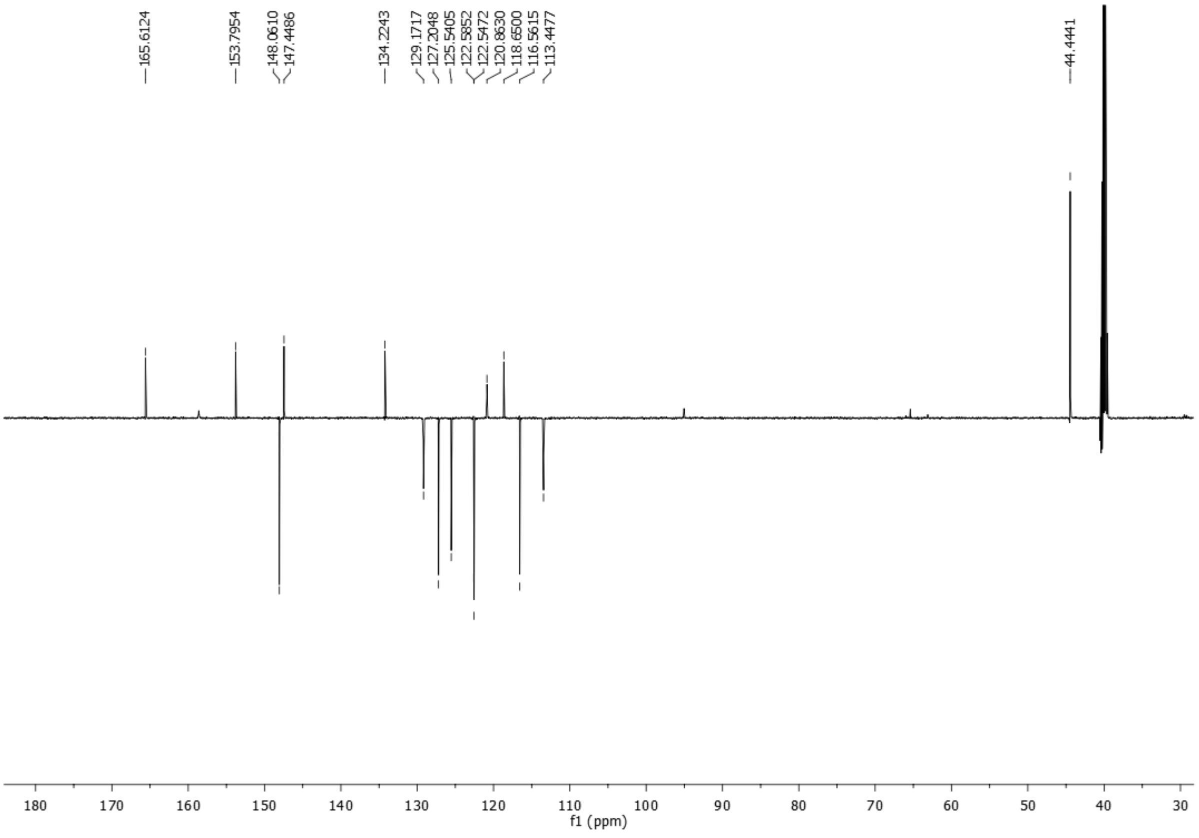

Figure S13. ^{1}H NMR spectrum (DMSO-d_6, 300 MHz) of ***(E)-2-(benzo[d]thiazol-2-yl)-3-(3,4,5-trihydroxyphenyl)acrylonitrile*** ***23***

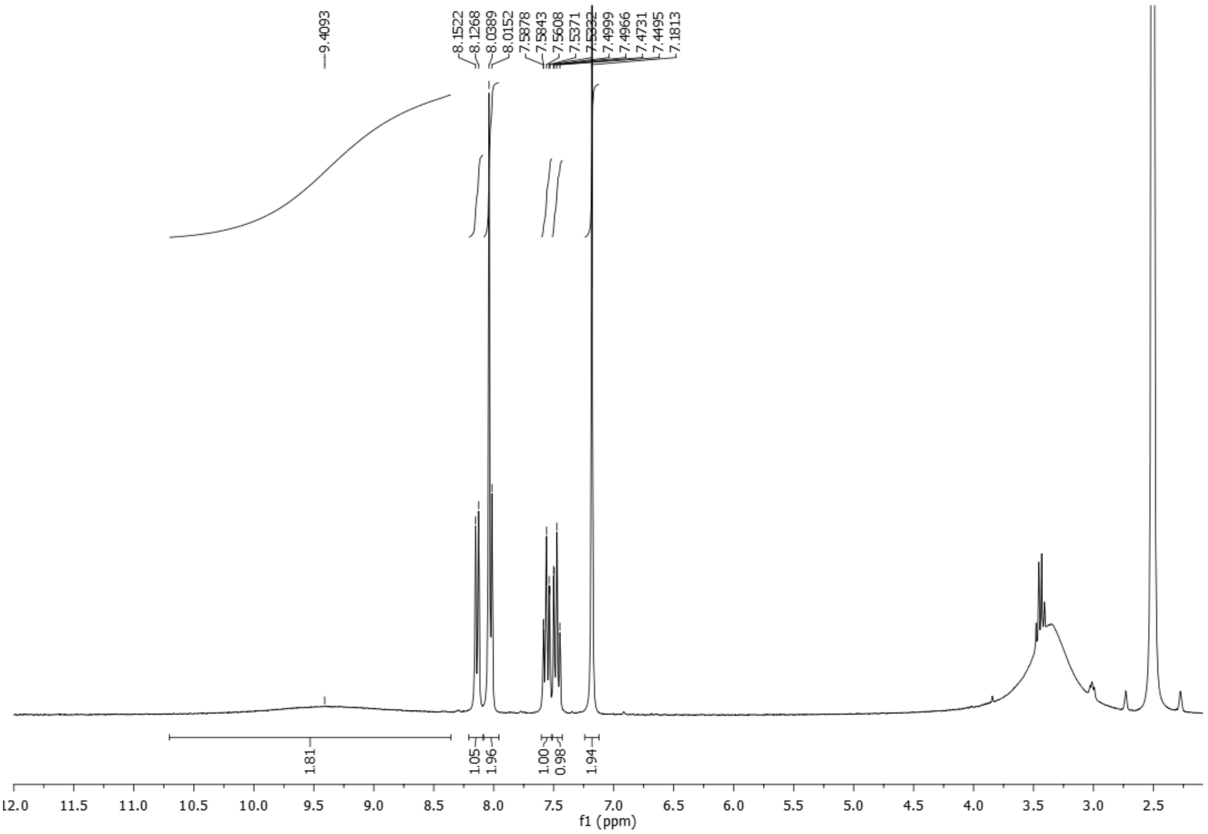

Figure S14. ^{13}C NMR spectrum (DMSO-d_6, 151 MHz) of ***(E)-2-(benzo[d]thiazol-2-yl)-3-(3,4,5-trihydroxyphenyl)acrylonitrile*** ***23***

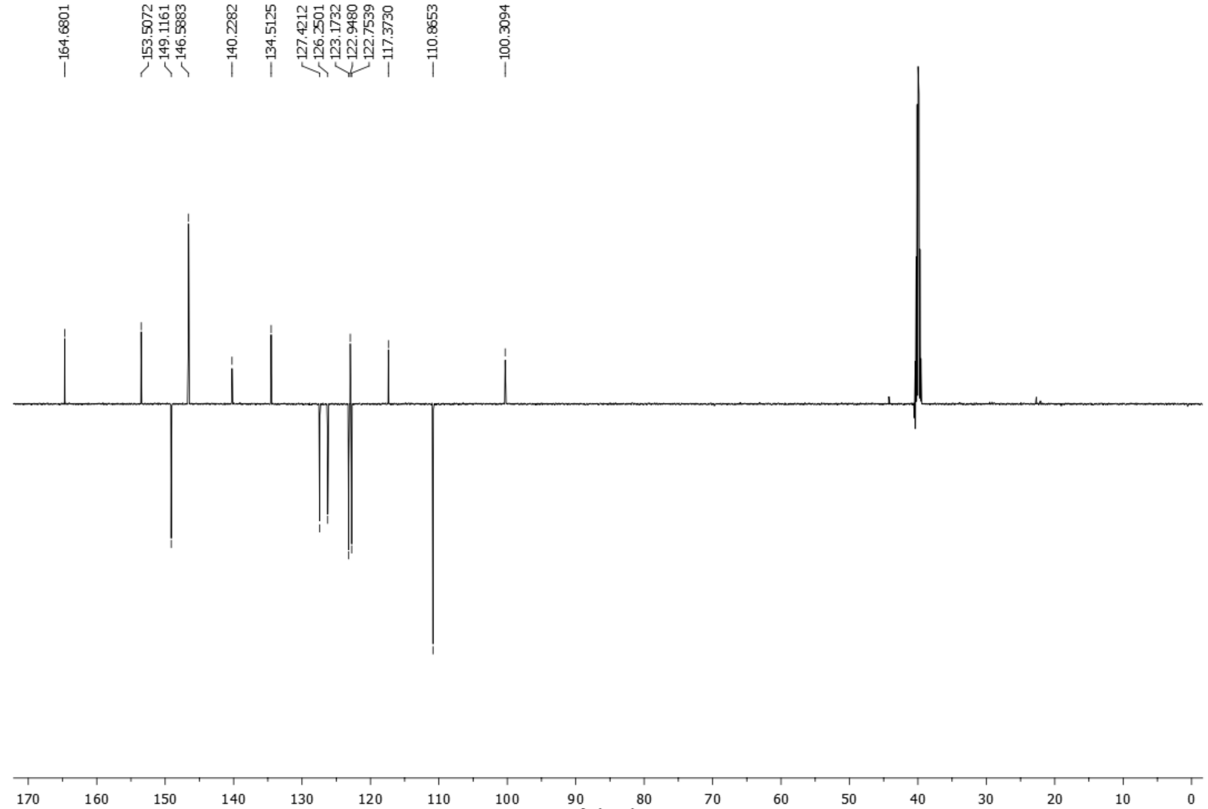

Figure S15. ^{1}H NMR spectrum (DMSO-d_6, 600 MHz) of ***(E)-2-(1H-benzo[d]imidazol-2-yl)-3-(4-hydroxyphenyl)acrylonitrile*** ***24***

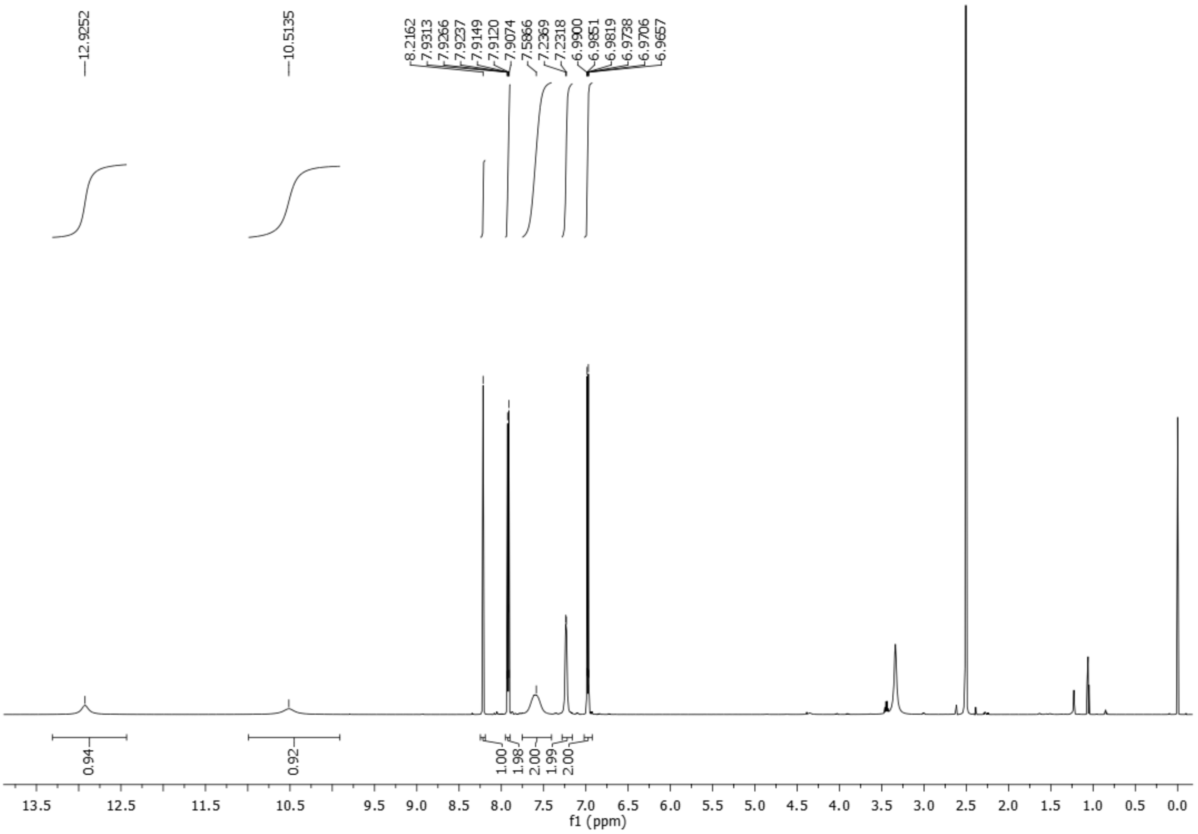

Figure S16. ^{13}C NMR spectrum (DMSO-d_6, 151 MHz) of ***(E)-2-(1H-benzo[d]imidazol-2-yl)-3-(4-hydroxyphenyl)acrylonitrile*** ***24***

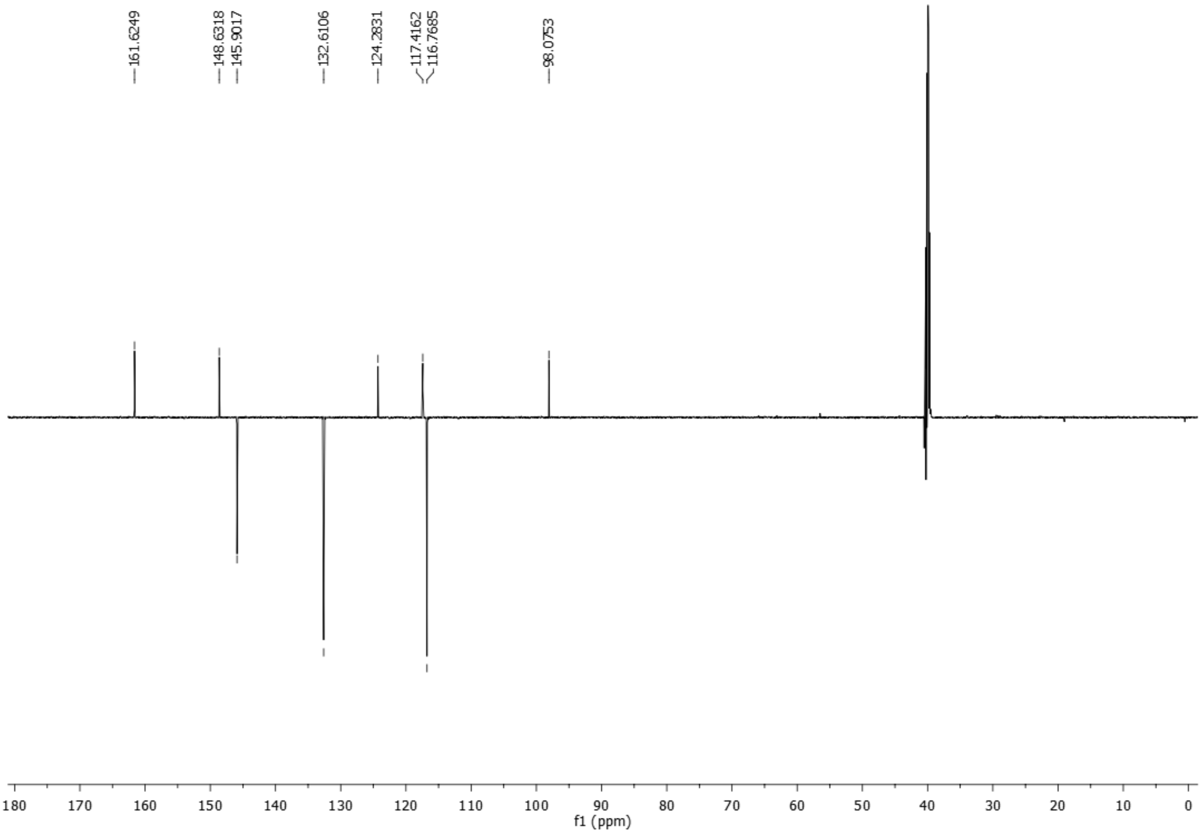

Figure S17. ^{1}H NMR spectrum (DMSO-d_6, 600 MHz) of ***(E)-2-(1H-benzo[d]imidazol-2-yl)-3-(3,4-dihydroxyphenyl)acrylonitrile*** **25**

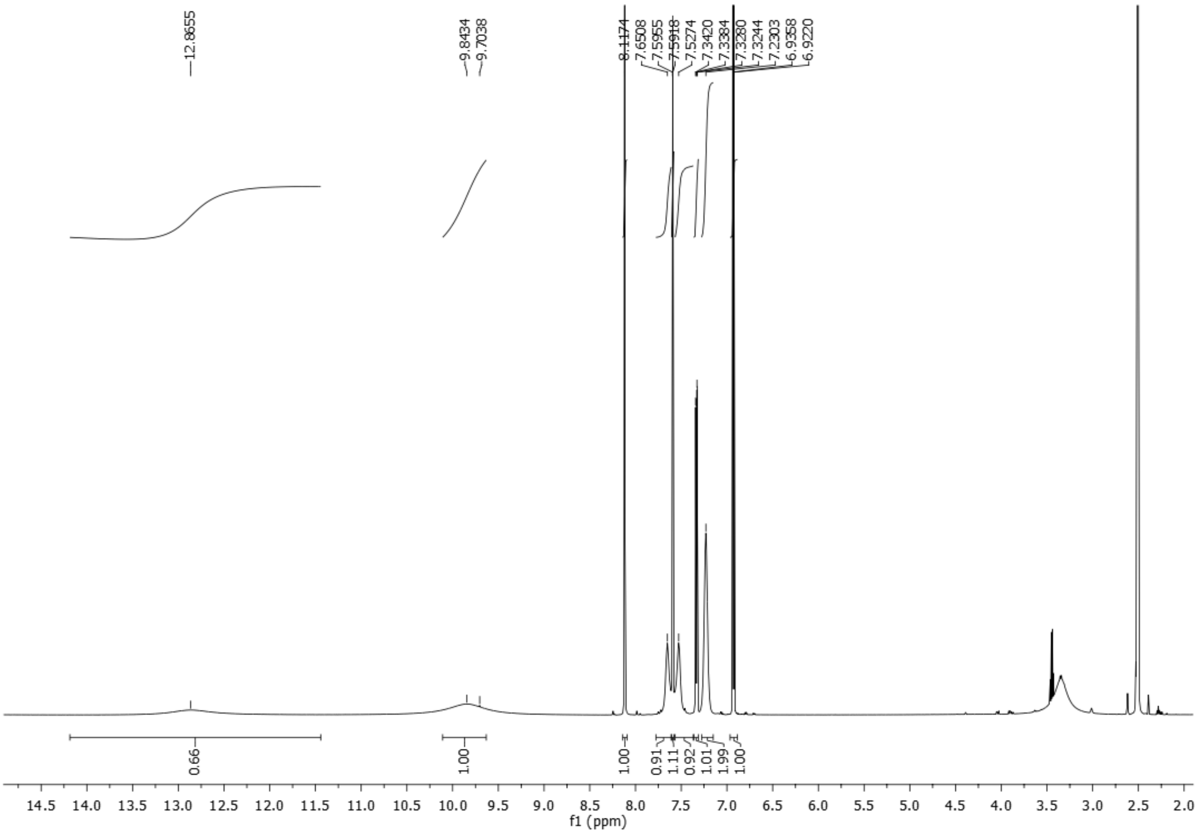

Figure S18. ^{13}C NMR spectrum (DMSO-d_6, 151 MHz) of ***(E)-2-(1H-benzo[d]imidazol-2-yl)-3-(3,4-dihydroxyphenyl)acrylonitrile*** **25**

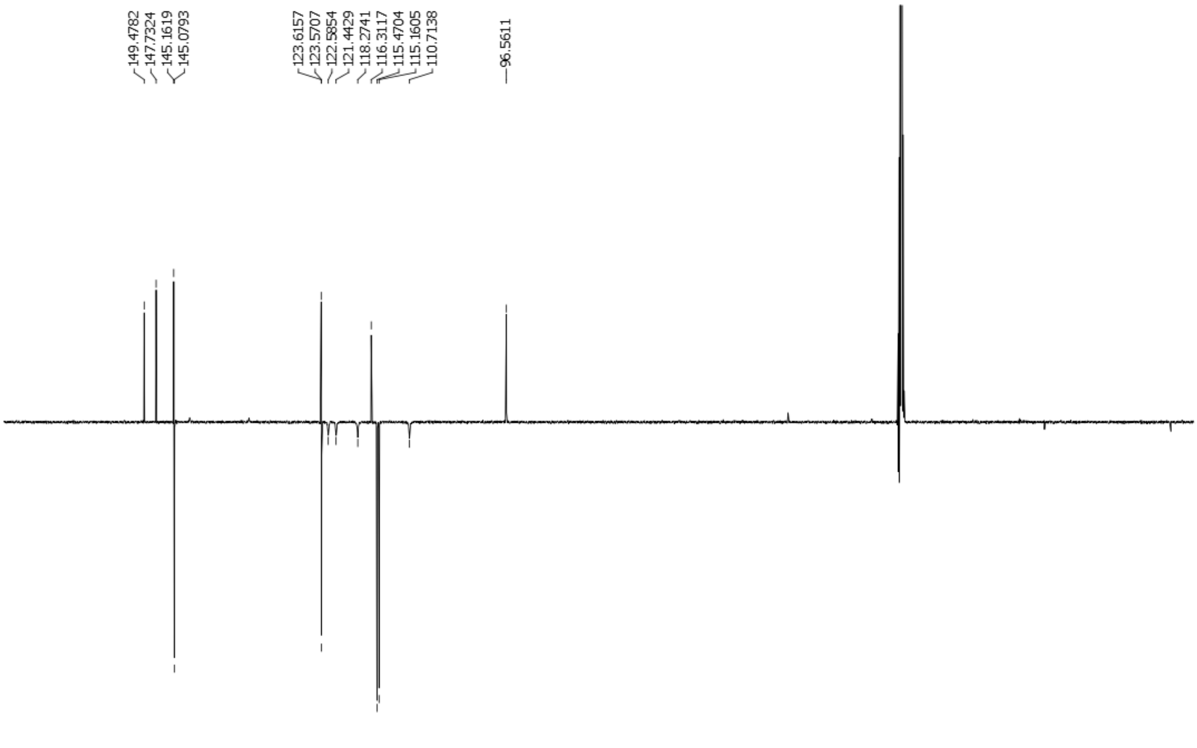

Figure S19. ^{1}H NMR spectrum (DMSO-d_6, 600 MHz) of ***(E)-2-(1H-benzo[d]imidazol-2-yl)-3-(3,4,5-trihydroxyphenyl)acrylonitrile 26***

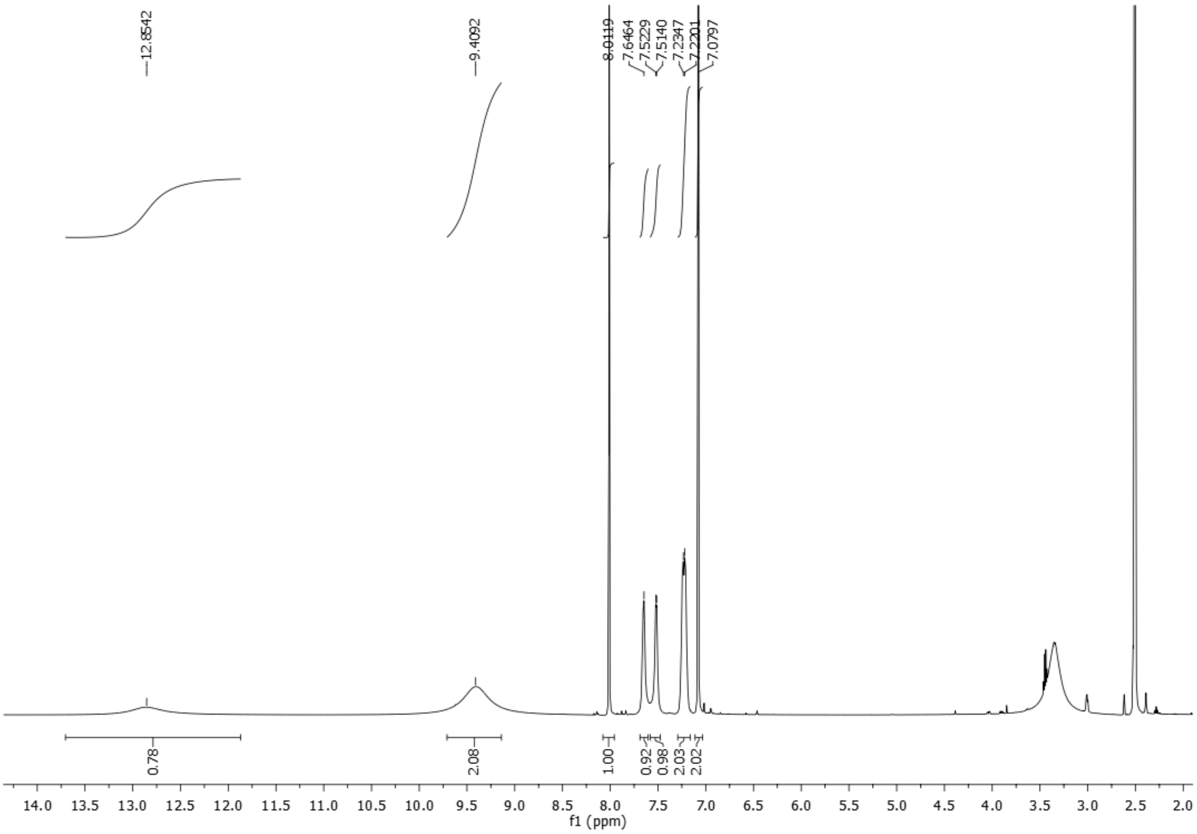

Figure S20. ^{13}C NMR spectrum (DMSO-d_6, 151 MHz) of ***(E)-2-(1H-benzo[d]imidazol-2-yl)-3-(3,4,5-trihydroxyphenyl)acrylonitrile 26***

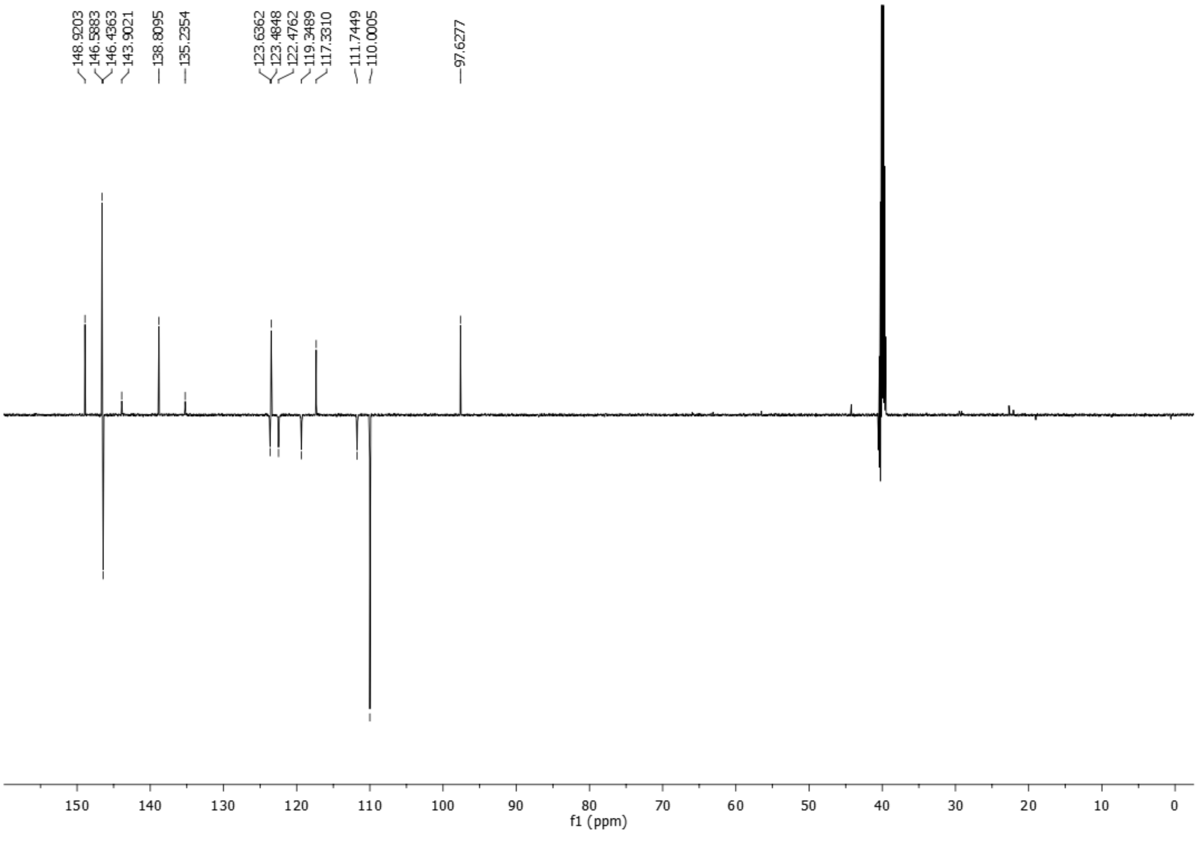

Figure S21. ^{1}H NMR spectrum (DMSO-d_6, 600 MHz) of ***(E)-2-(1-cyano-2-(4-hydroxyphenyl)vinyl)-1H-benzo[d]imidazole-5(6)-carbonitrile*** ***27***

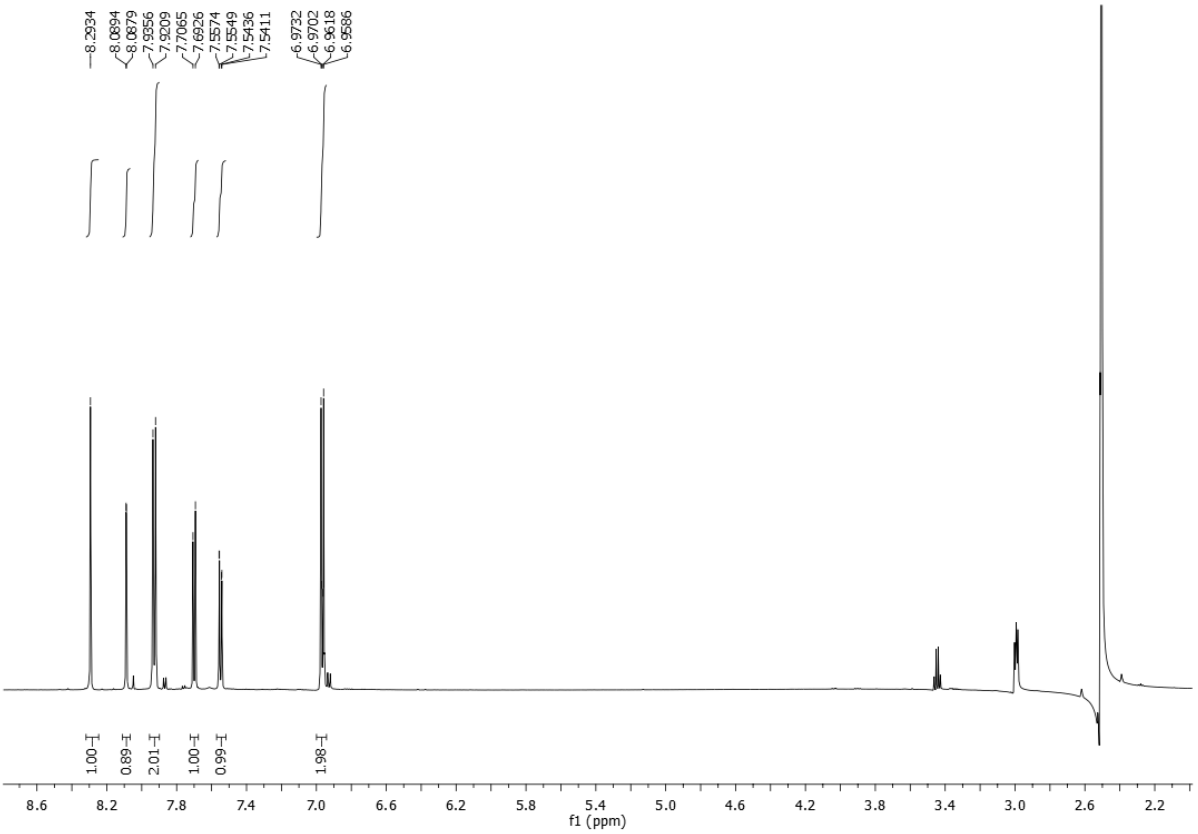

Figure S22. ^{13}C NMR spectrum (DMSO-d_6, 151 MHz) of ***(E)-2-(1-cyano-2-(4-hydroxyphenyl)vinyl)-1H-benzo[d]imidazole-5(6)-carbonitrile*** ***27***

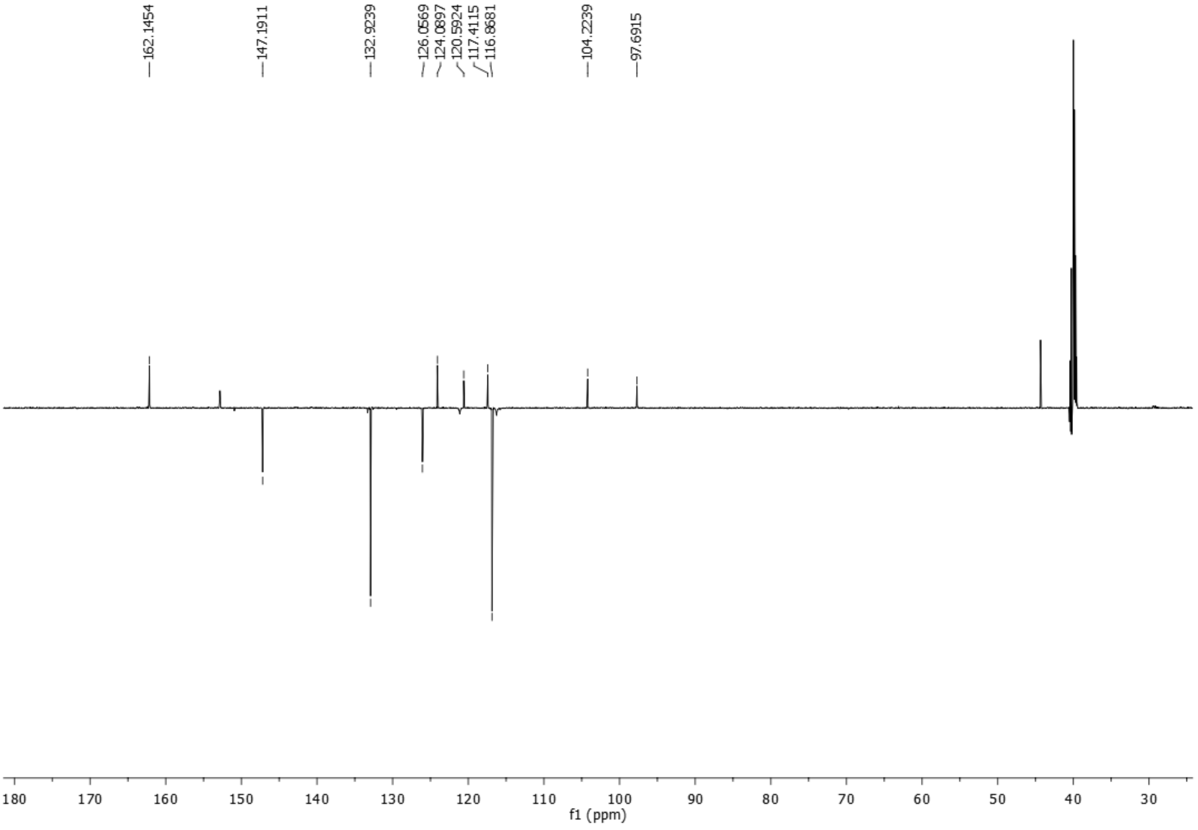

Figure S23. ^{1}H NMR spectrum (DMSO-d_6, 600 MHz) of ***(E)-2-(1-cyano-2-(3,4-dihydroxyphenyl)vinyl)-1H-benzo[d]imidazole-5(6)-carbonitrile* 28**

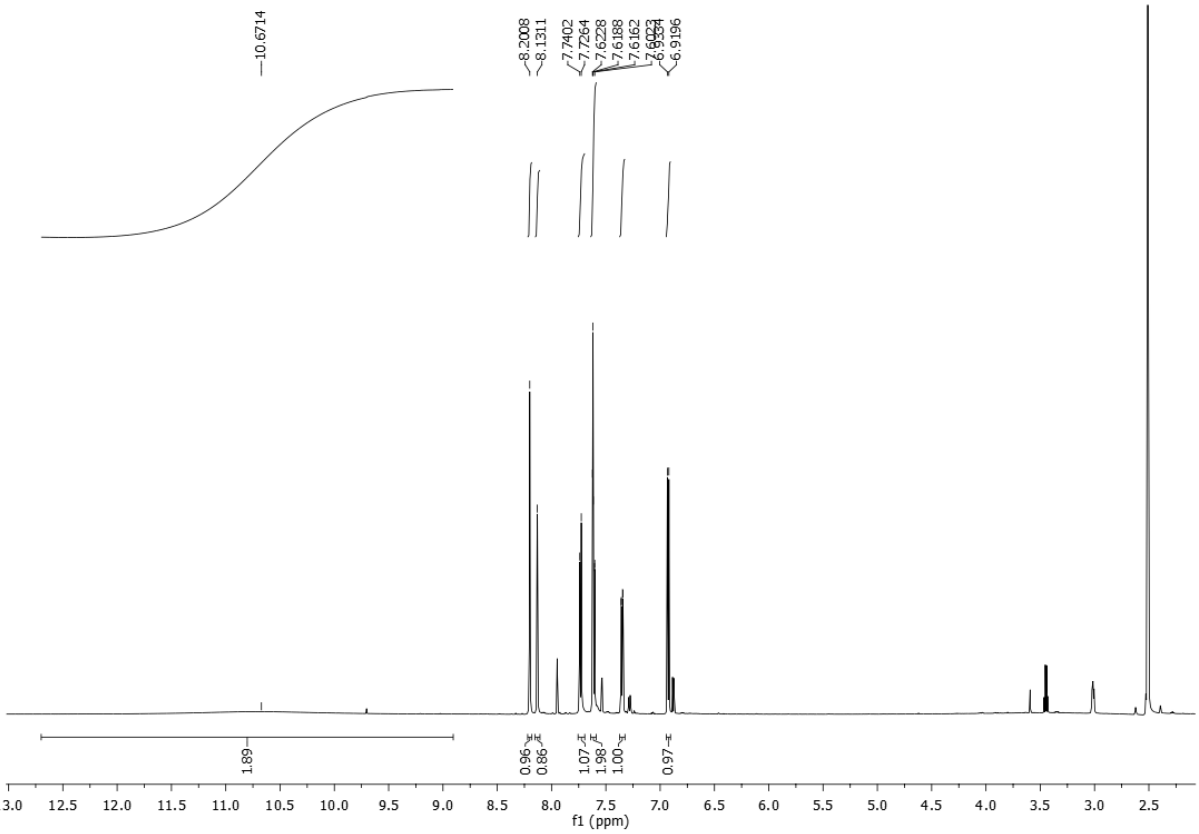

Figure S24. ^{13}C NMR spectrum (DMSO-d_6, 151 MHz) of ***(E)-2-(1-cyano-2-(3,4-dihydroxyphenyl)vinyl)-1H-benzo[d]imidazole-5(6)-carbonitrile* 28**

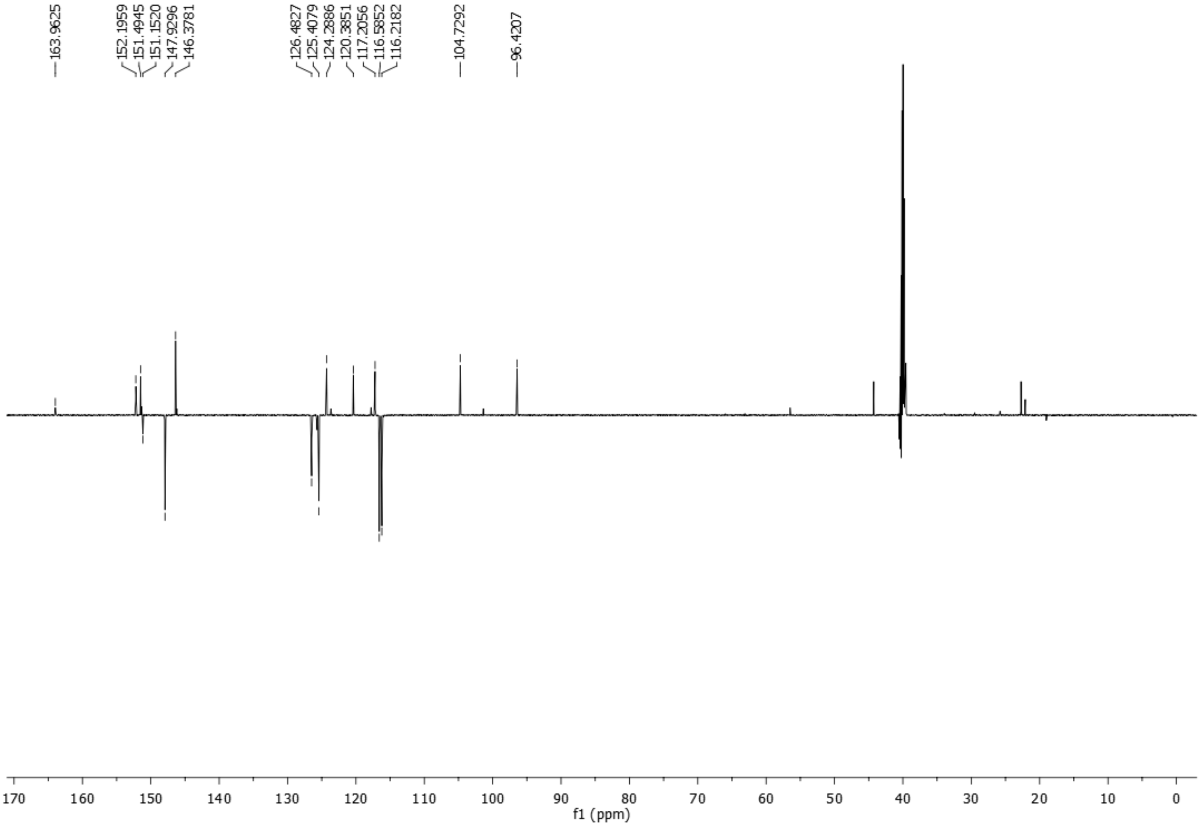

Figure S25. ^{1}H NMR spectrum (DMSO-d_6, 600 MHz) of ***(E)-2-(1-cyano-2-(3,4,5-trihydroxyphenyl)vinyl)-1H-benzo[d]imidazole-5(6)-carbonitrile 29***

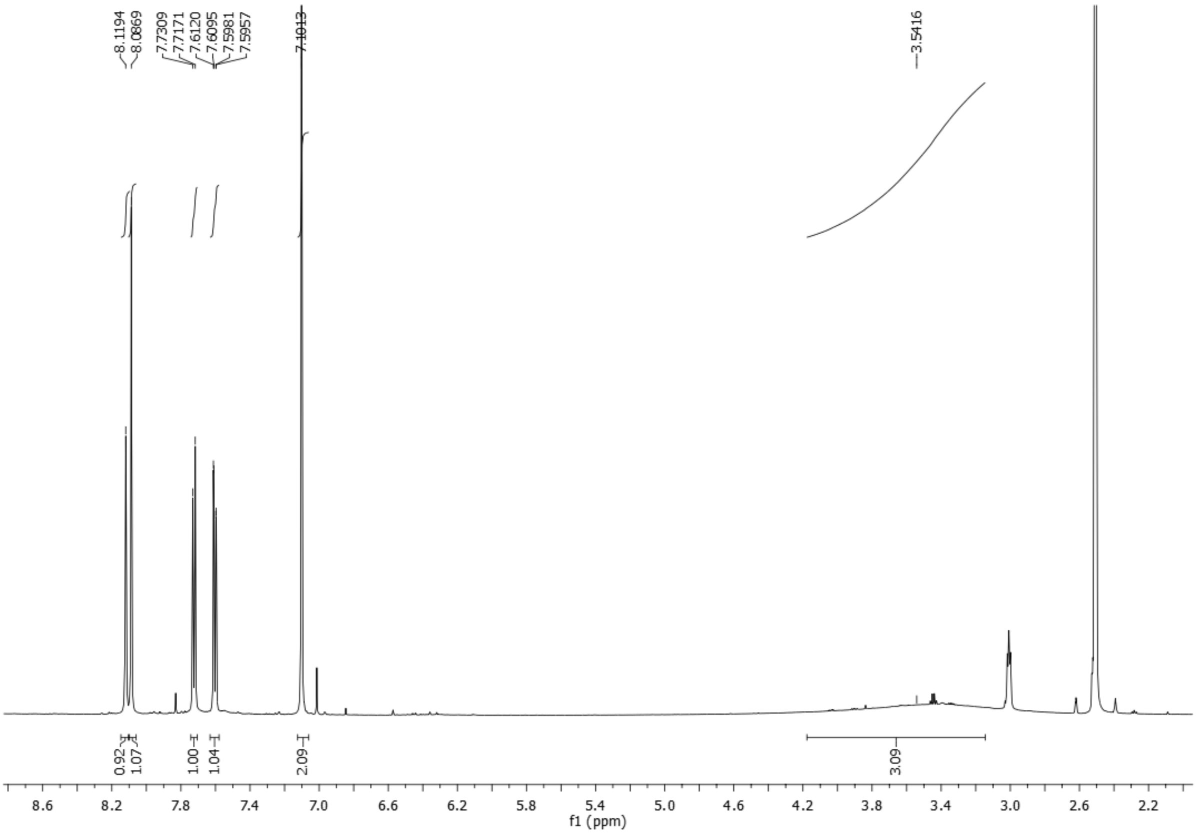

Figure S26. ^{13}C NMR spectrum (DMSO-d_6, 151 MHz) of ***(E)-2-(1-cyano-2-(3,4,5-trihydroxyphenyl)vinyl)-1H-benzo[d]imidazole-5(6)-carbonitrile 29***

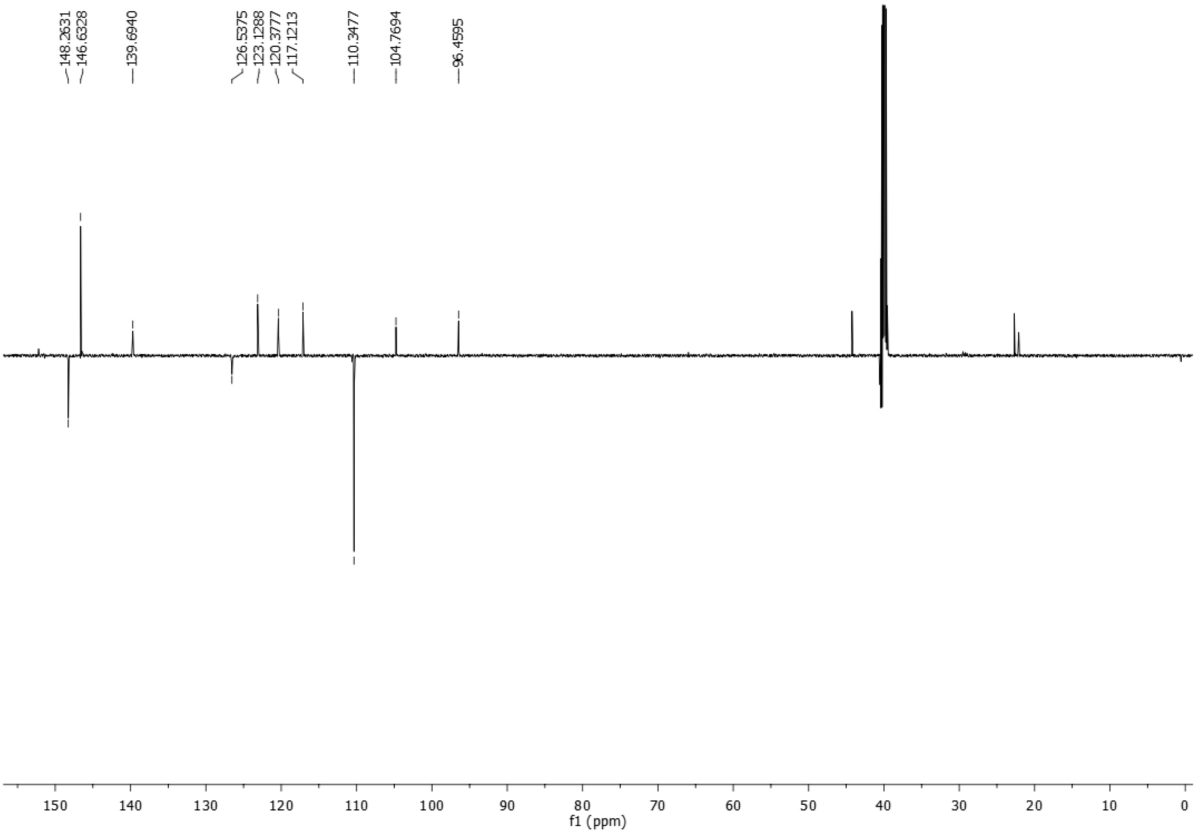

Figure S27. ^{1}H NMR spectrum (DMSO-d_6, 600 MHz) of ***(E)-3-(4-hydroxyphenyl)-2-(1-isobutyl-1H-benzo[d]imidazol-2-yl)acrylonitrile*** **30**

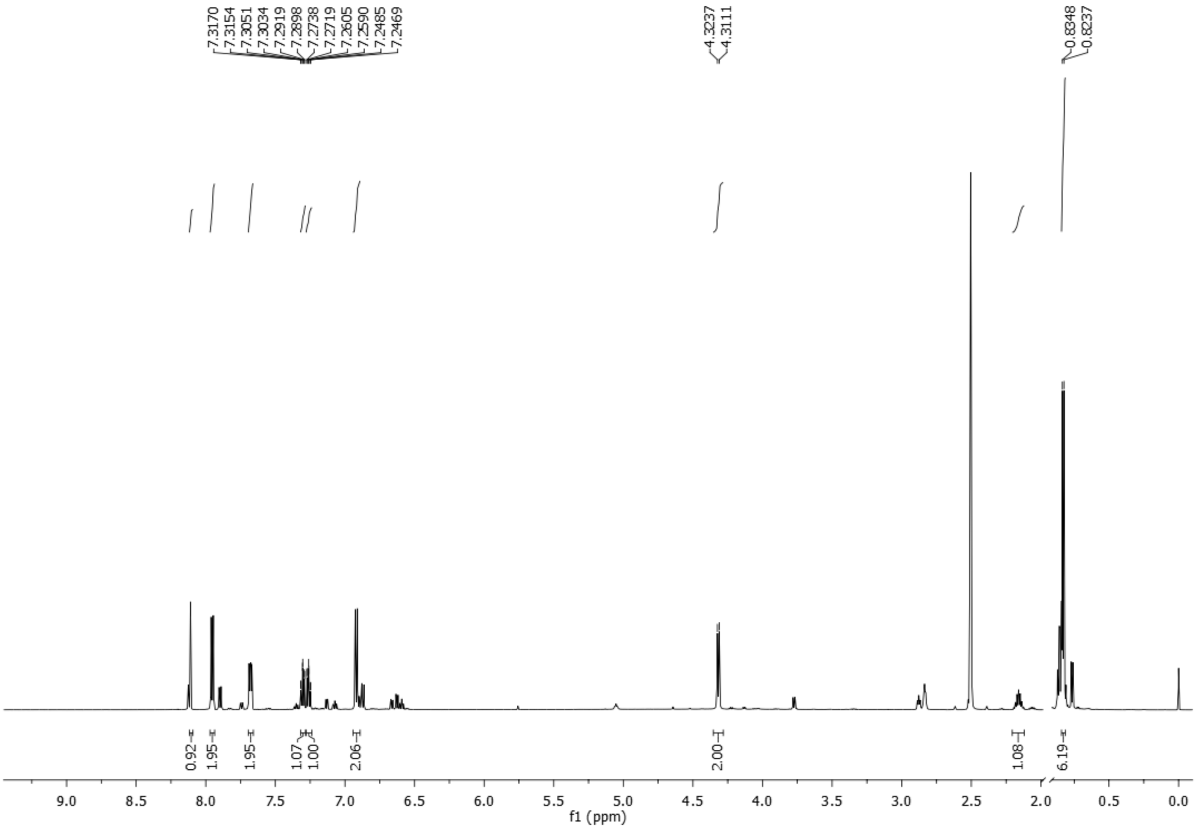

Figure S28. ^{13}C NMR spectrum (DMSO-d_6, 151 MHz) of ***(E)-3-(4-hydroxyphenyl)-2-(1-isobutyl-1H-benzo[d]imidazol-2-yl)acrylonitrile*** **30**

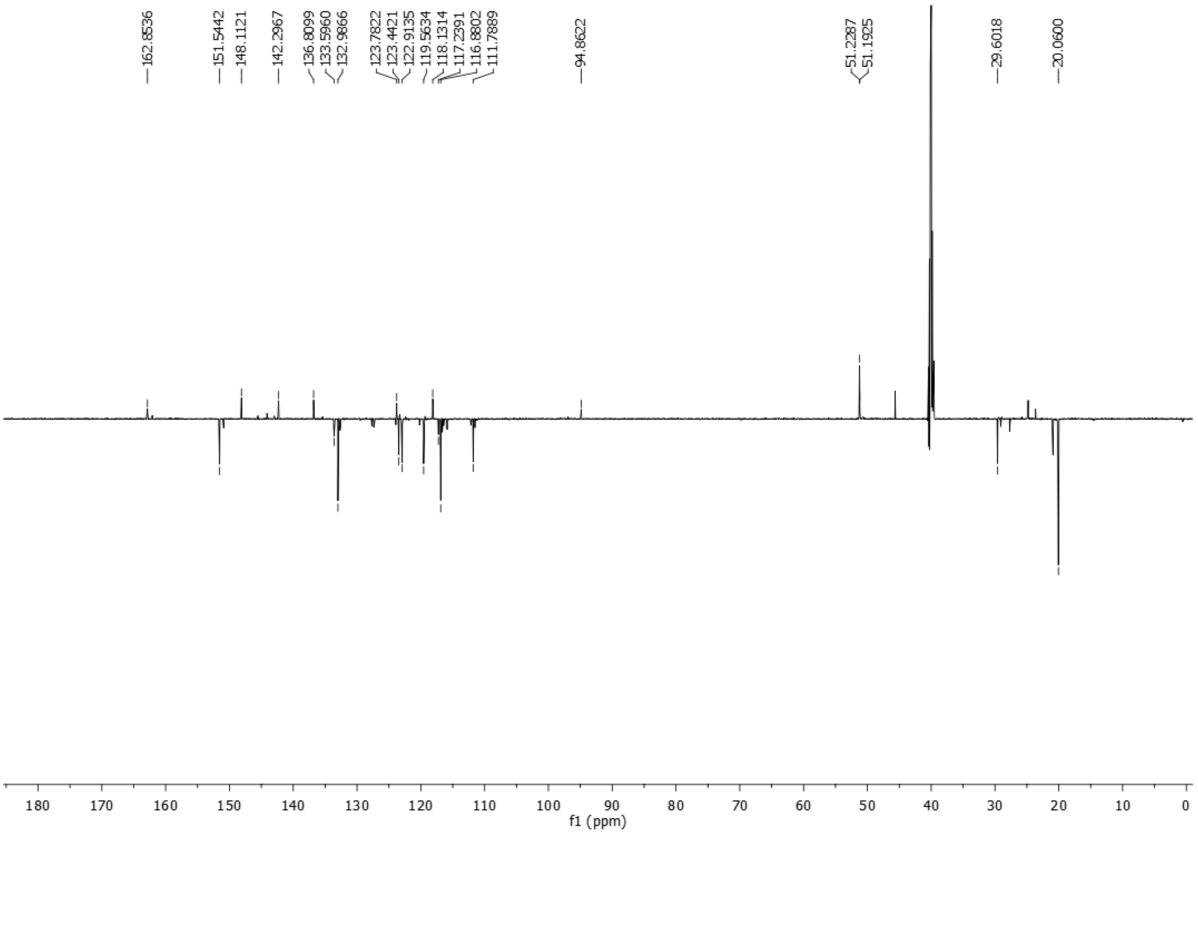

Figure S29. ^{1}H NMR spectrum (DMSO-d_6, 600 MHz) of ***(E)-3-(3,4-dihydroxyphenyl)-2-(1-isobutyl-1H-benzo[d]imidazol-2-yl)acrylonitrile*** ***31***

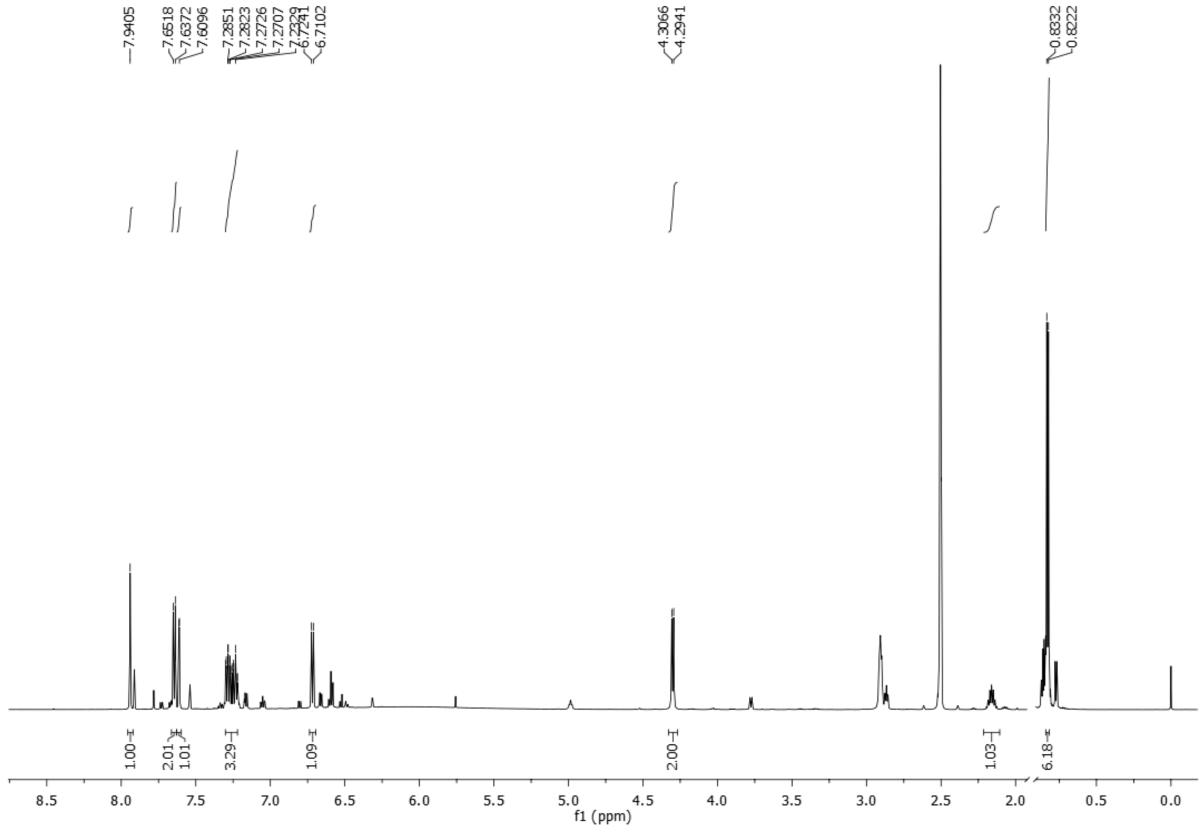

Figure S30. ^{13}C NMR spectrum (DMSO-d_6, 151 MHz) of ***(E)-3-(3,4-dihydroxyphenyl)-2-(1-isobutyl-1H-benzo[d]imidazol-2-yl)acrylonitrile*** ***31***

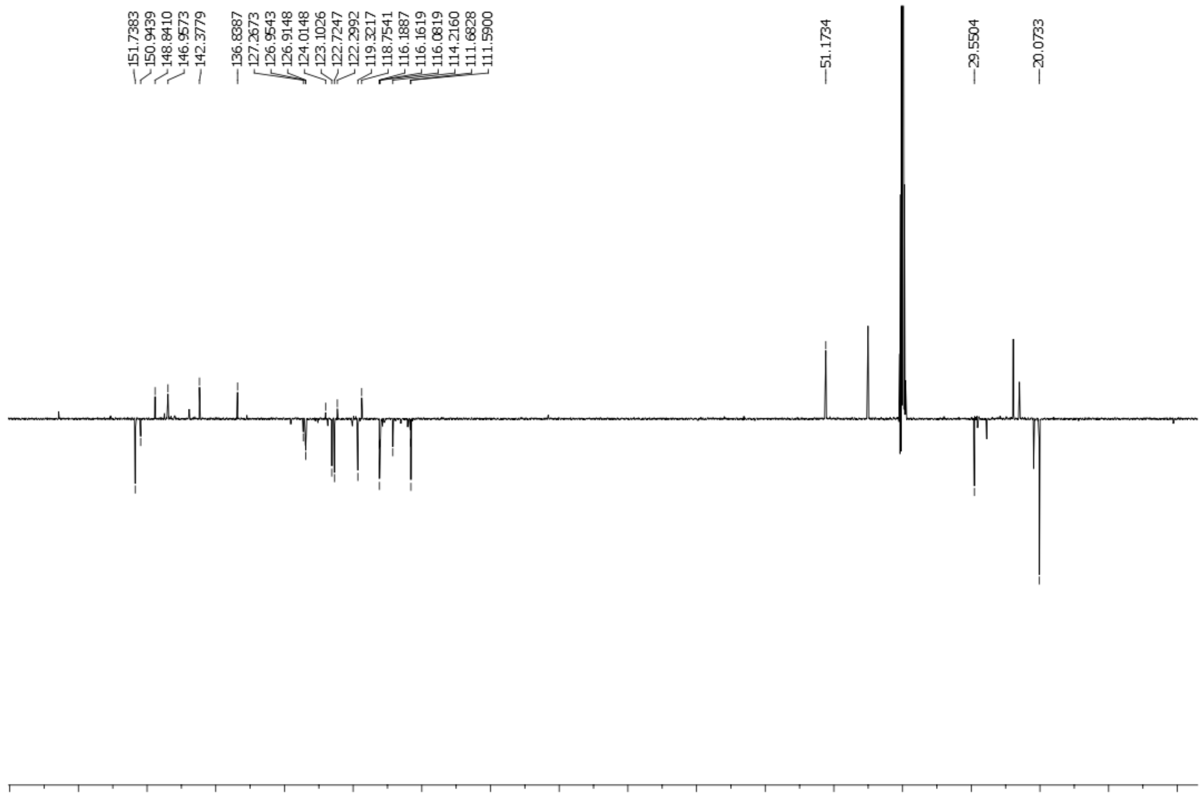

Figure S31. ^{1}H NMR spectrum (DMSO-d_6, 600 MHz) of ***(E)-2-(1-isobutyl-1H-benzo[d]imidazol-2-yl)-3-(3,4,5-trihydroxyphenyl)acrylonitrile 32***

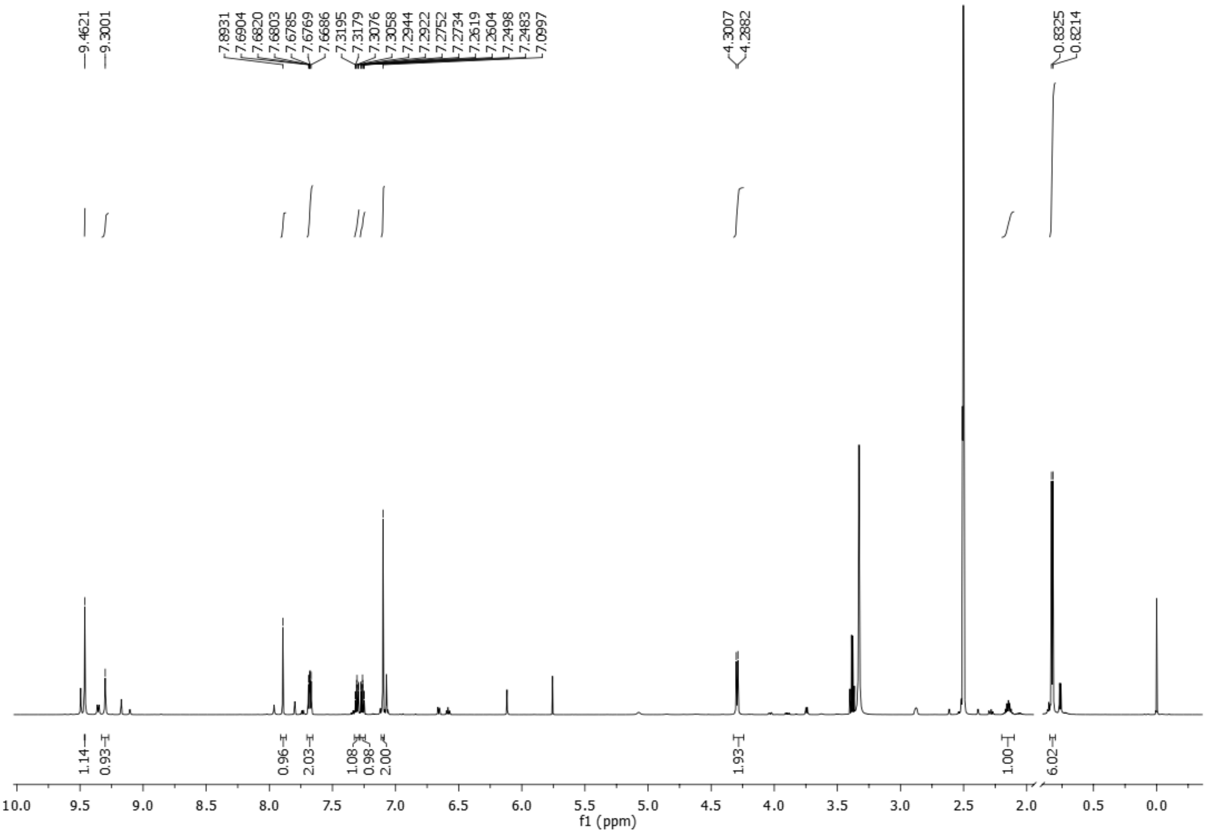

Figure S32. ^{13}C NMR spectrum (DMSO-d_6, 151 MHz) of ***(E)-2-(1-isobutyl-1H-benzo[d]imidazol-2-yl)-3-(3,4,5-trihydroxyphenyl)acrylonitrile 32***

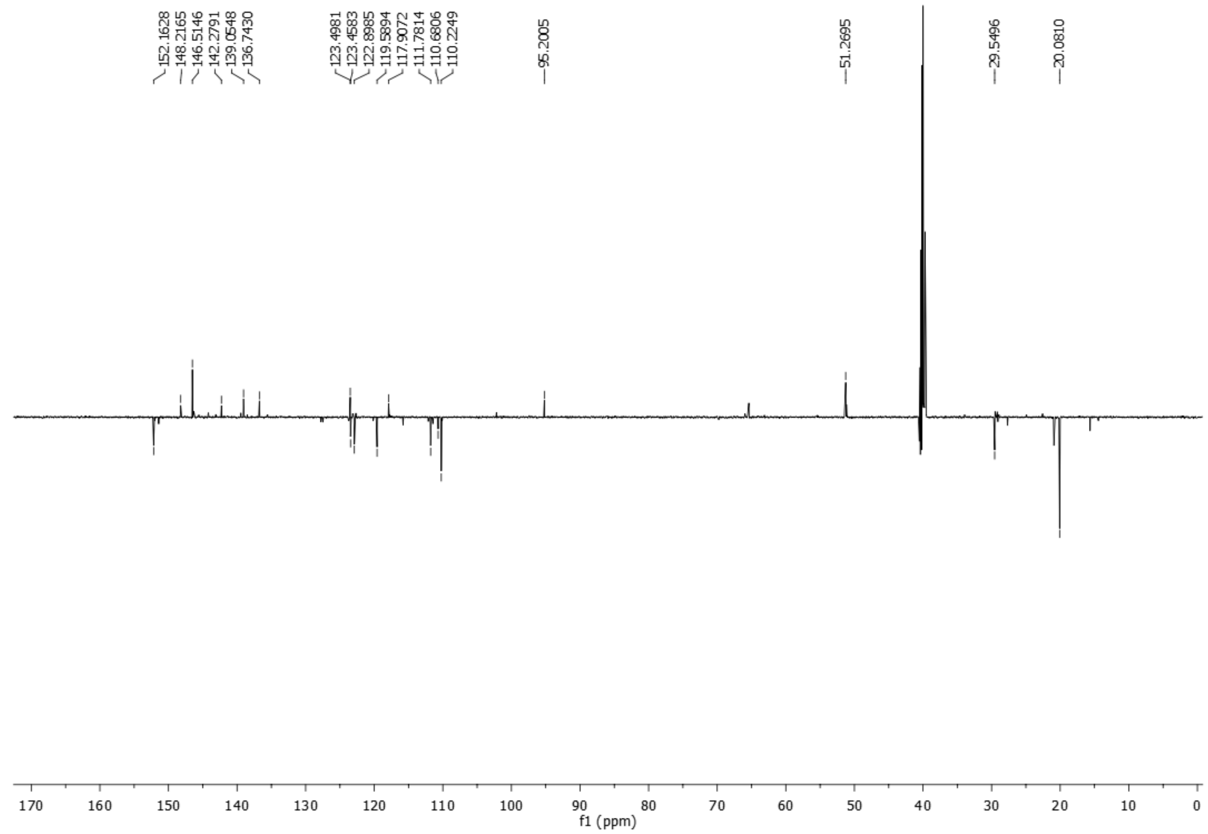

Figure S33. ^{1}H NMR spectrum (DMSO-d_6, 600 MHz) of ***(E)-2-(1-cyano-2-(4-hydroxyphenyl)vinyl)-1-isobutyl-1H-benzo[d]imidazole-6-carbonitrile 33***

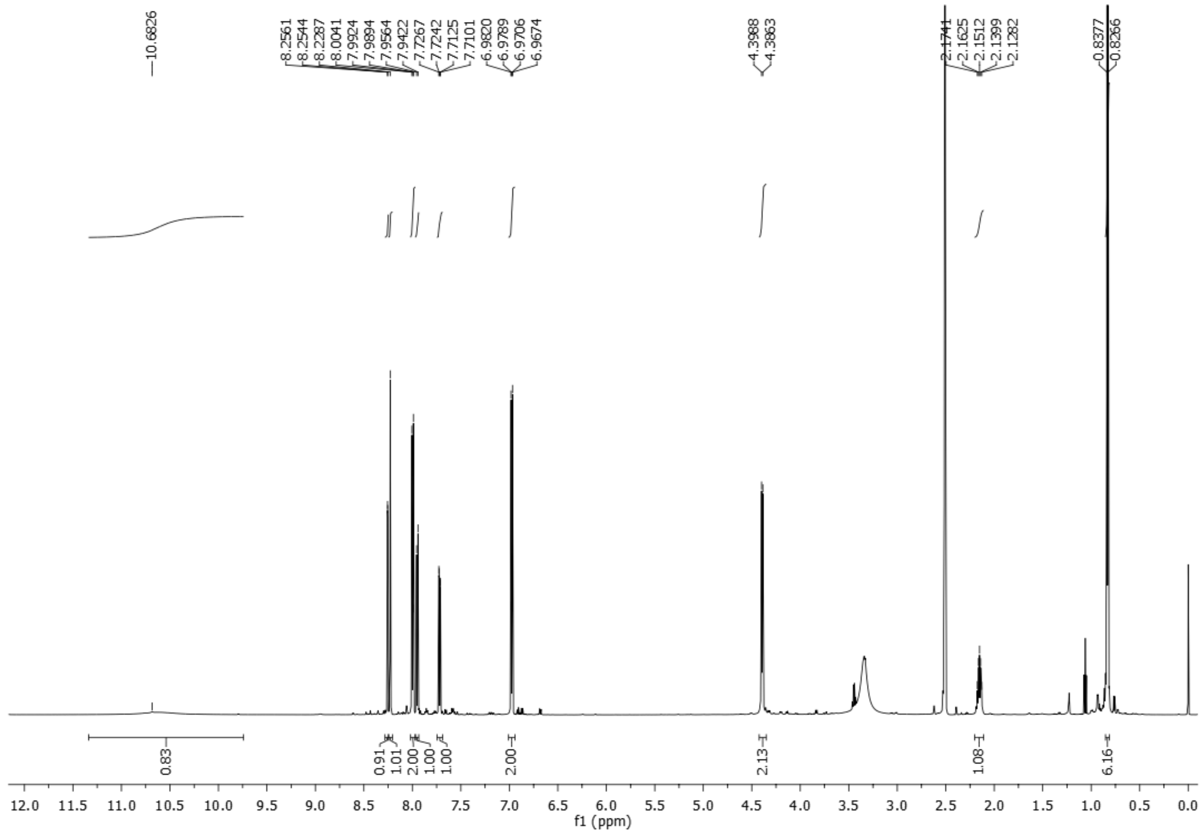

Figure S34. ^{13}C NMR spectrum (DMSO-d_6, 151 MHz) of ***(E)-2-(1-cyano-2-(4-hydroxyphenyl)vinyl)-1-isobutyl-1H-benzo[d]imidazole-6-carbonitrile 33***

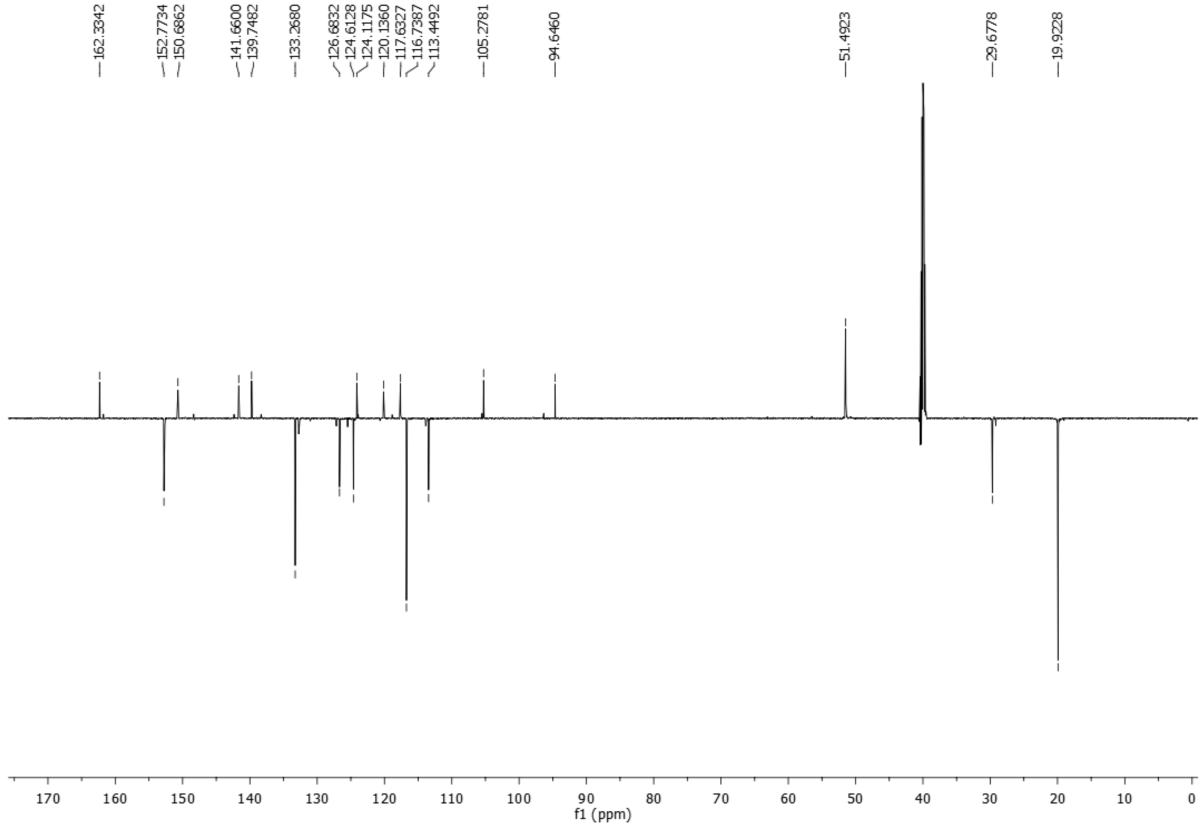

Figure S35. ^{1}H NMR spectrum (DMSO-d_6, 600 MHz) of ***(E)-2-(1-cyano-2-(3,4-dihydroxyphenyl)vinyl)-1-isobutyl-1H-benzo[d]imidazole-6-carbonitrile 34***

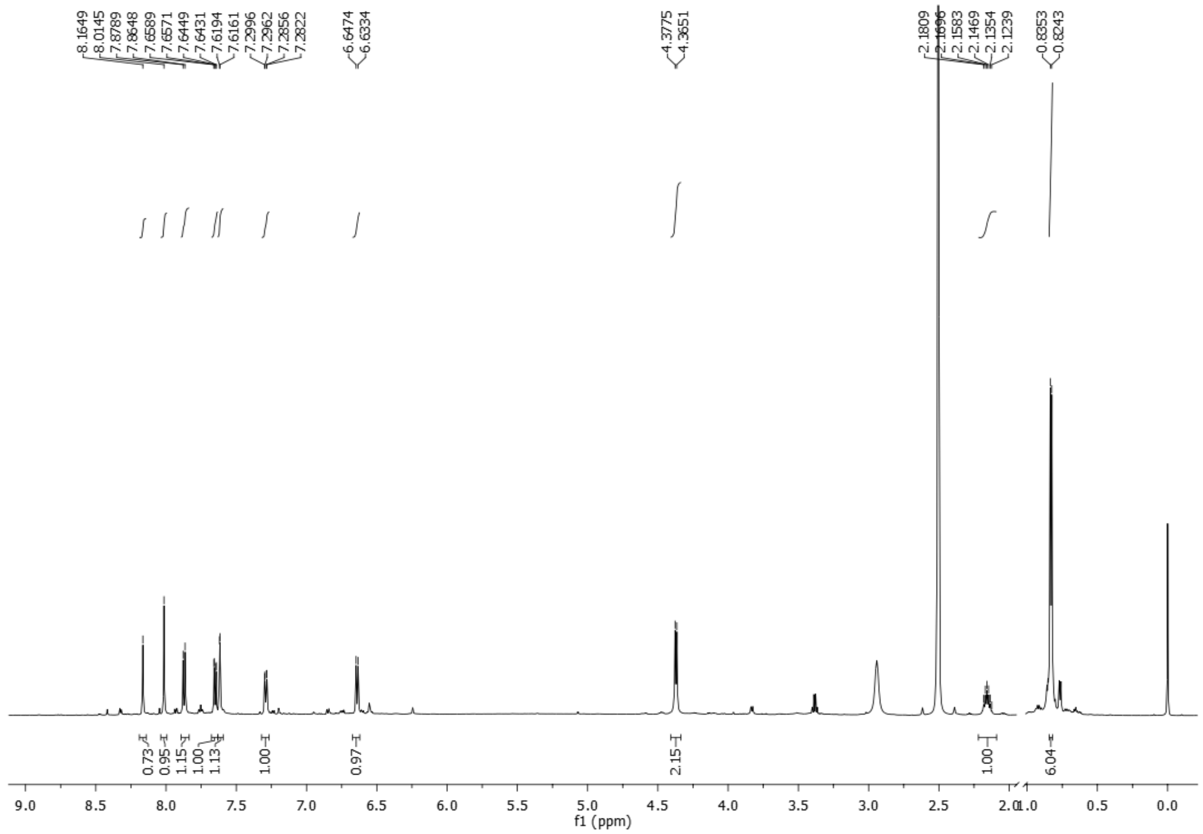

Figure S36. ^{13}C NMR spectrum (DMSO-d_6, 151 MHz) of ***(E)-2-(1-cyano-2-(3,4-dihydroxyphenyl)vinyl)-1-isobutyl-1H-benzo[d]imidazole-6-carbonitrile 34***

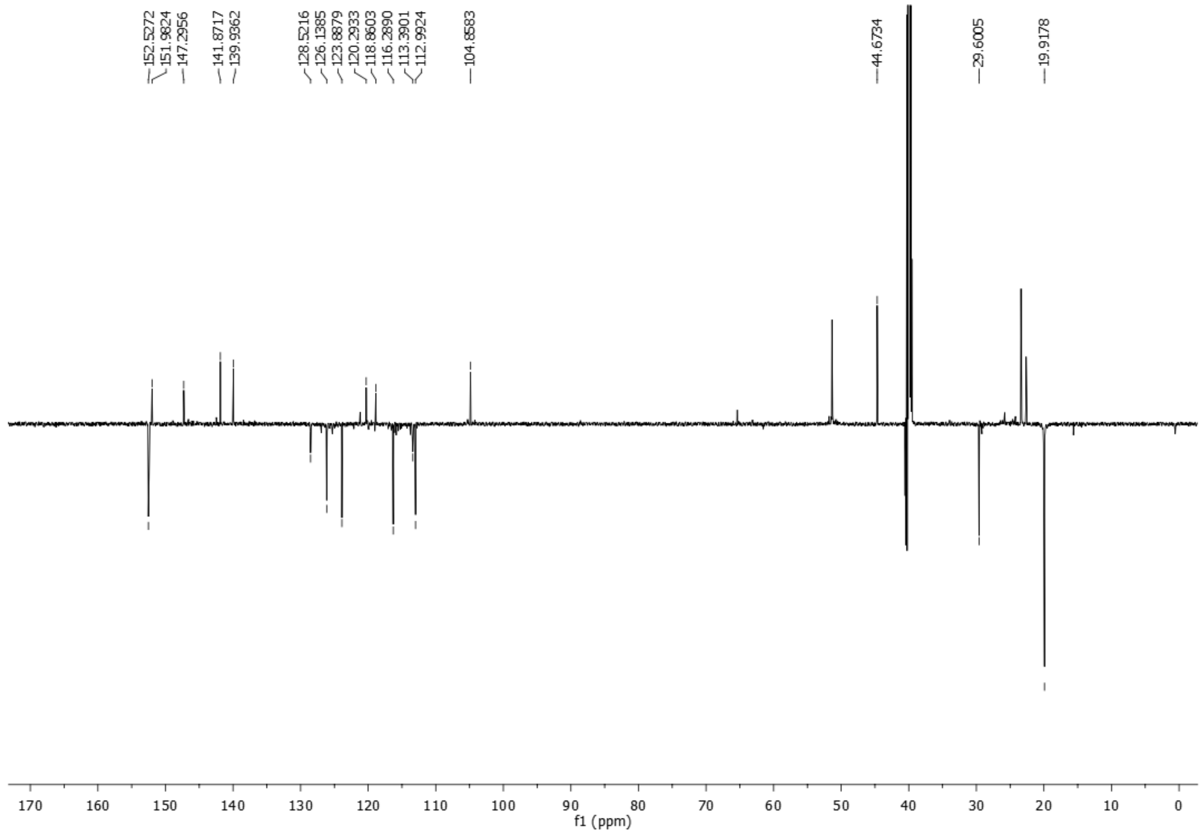

Figure S37. ^{1}H NMR spectrum (DMSO-d_6, 600 MHz) of ***(E)-2-(1-cyano-2-(3,4,5-trihydroxyphenyl)vinyl)-1-isobutyl-1H-benzo[d]imidazole-6-carbonitrile*** ***35***

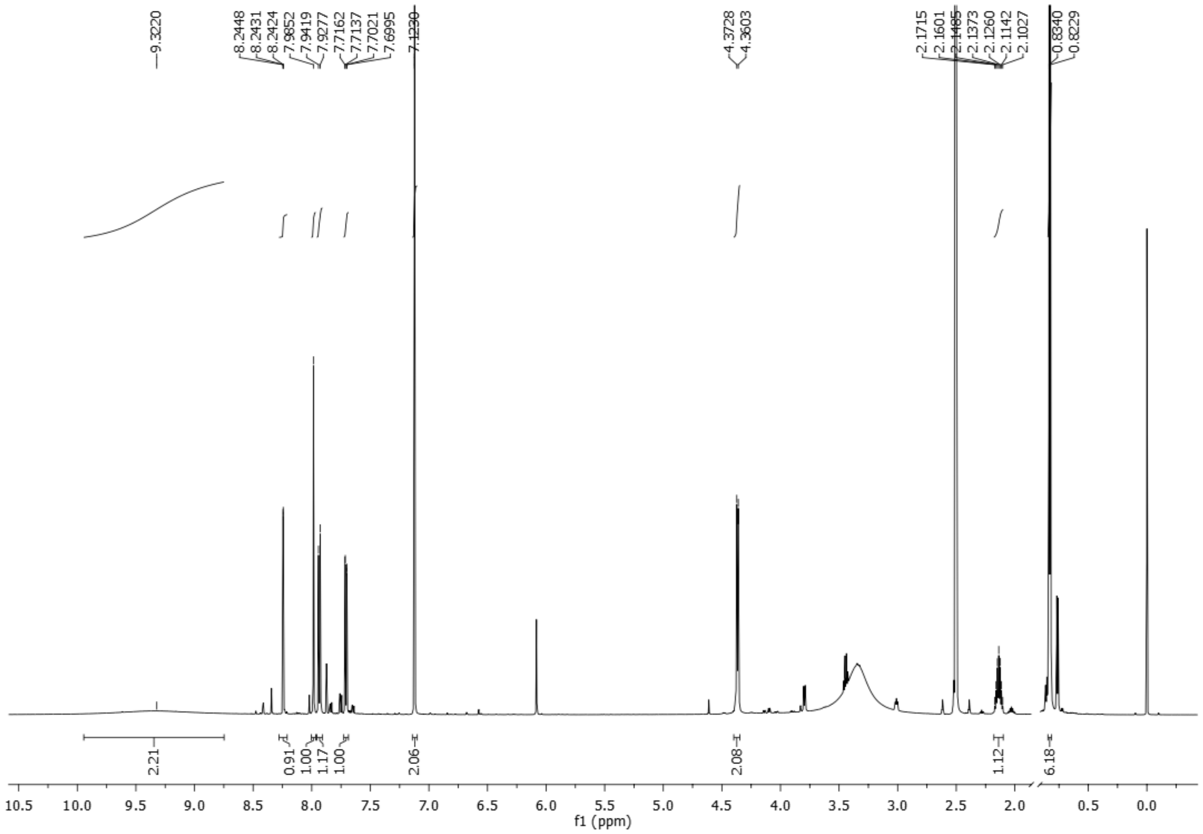

Figure S38. ^{13}C NMR spectrum (DMSO-d_6, 151 MHz) of ***(E)-2-(1-cyano-2-(3,4,5-trihydroxyphenyl)vinyl)-1-isobutyl-1H-benzo[d]imidazole-6-carbonitrile*** ***35***

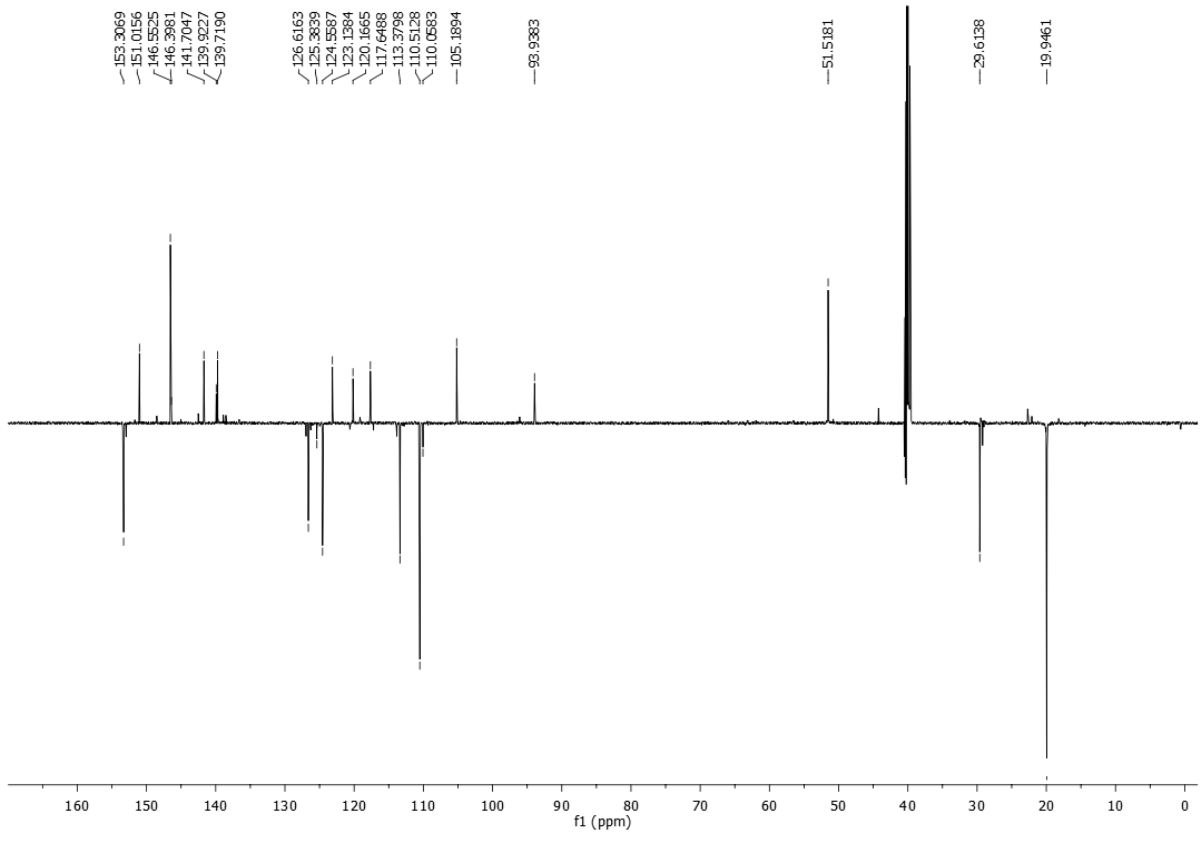

Figure S39. ^{1}H NMR spectrum (DMSO-d_6, 600 MHz) of ***(E)-3-(4-hydroxyphenyl)-2-(1-methyl-1H-benzo[d]imidazol-2-yl)acrylonitrile 36***

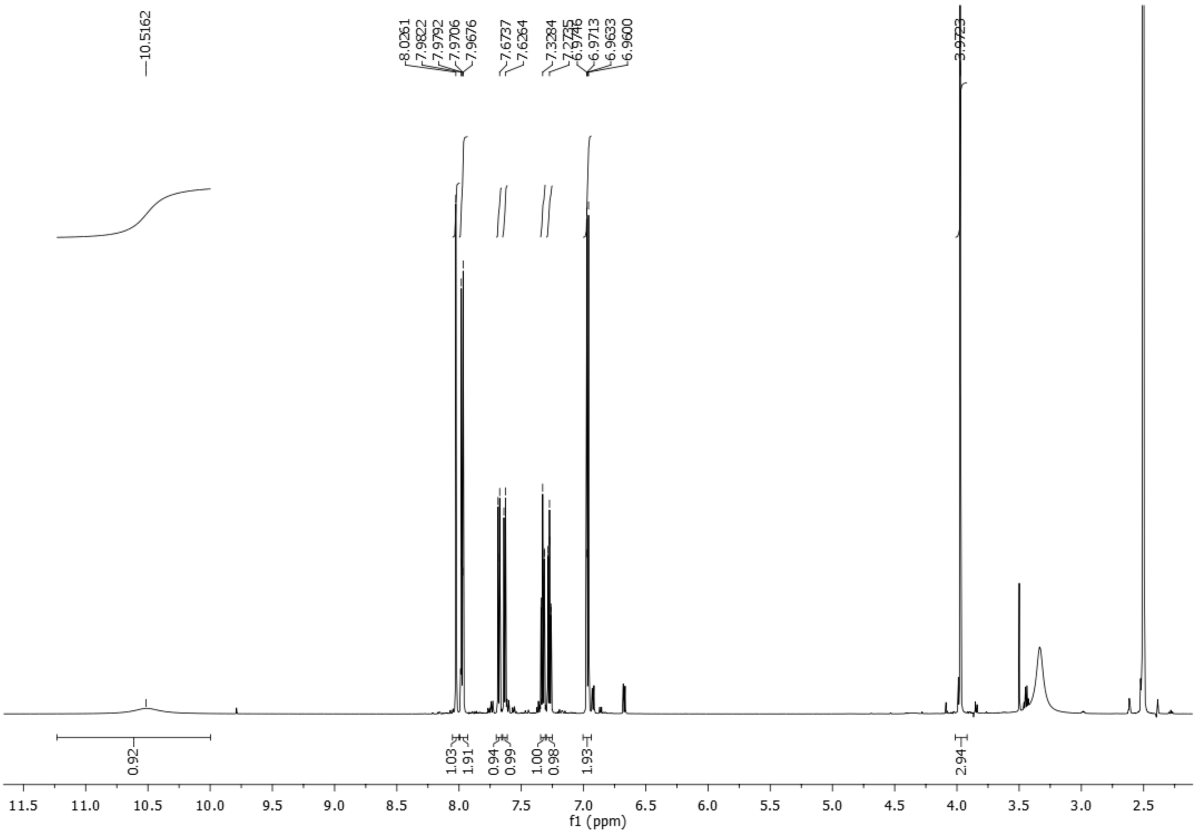

Figure S40. ^{13}C NMR spectrum (DMSO-d_6, 151 MHz) of ***(E)-3-(4-hydroxyphenyl)-2-(1-methyl-1H-benzo[d]imidazol-2-yl)acrylonitrile 36***

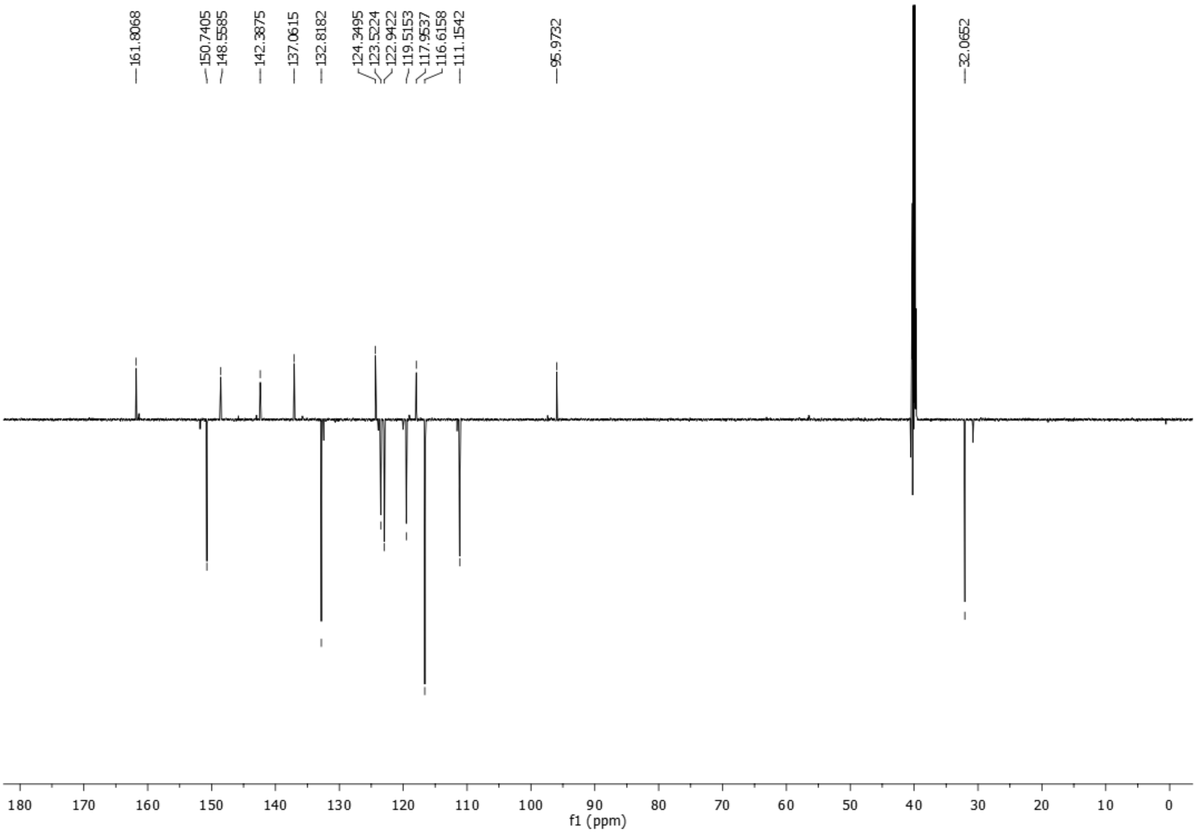

Figure S41. ^{1}H NMR spectrum (DMSO-d_6, 600 MHz) of ***(E)-3-(3,4-dihydroxyphenyl)-2-(1-methyl-1H-benzo[d]imidazol-2-yl)acrylonitrile*** ***37***

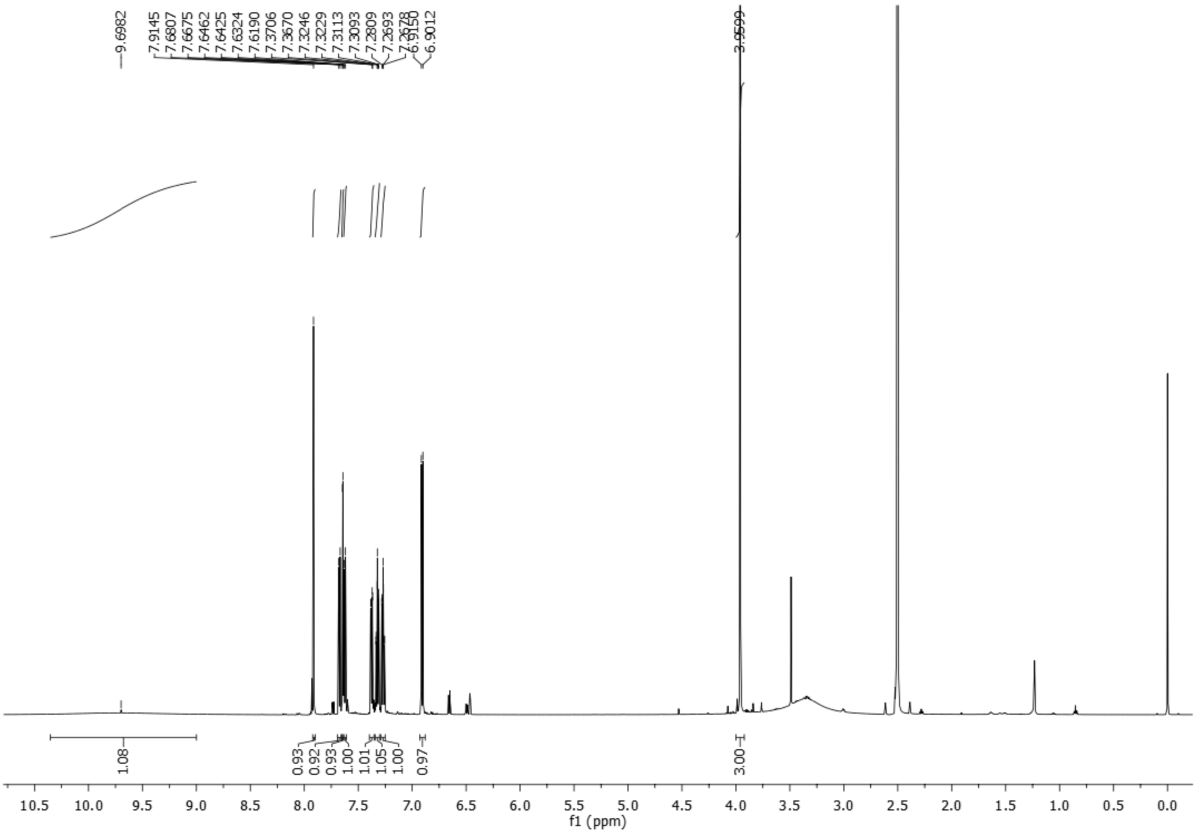

Figure S42. ^{13}C NMR spectrum (DMSO-d_6, 151 MHz) of ***(E)-3-(3,4-dihydroxyphenyl)-2-(1-methyl-1H-benzo[d]imidazol-2-yl)acrylonitrile*** ***37***

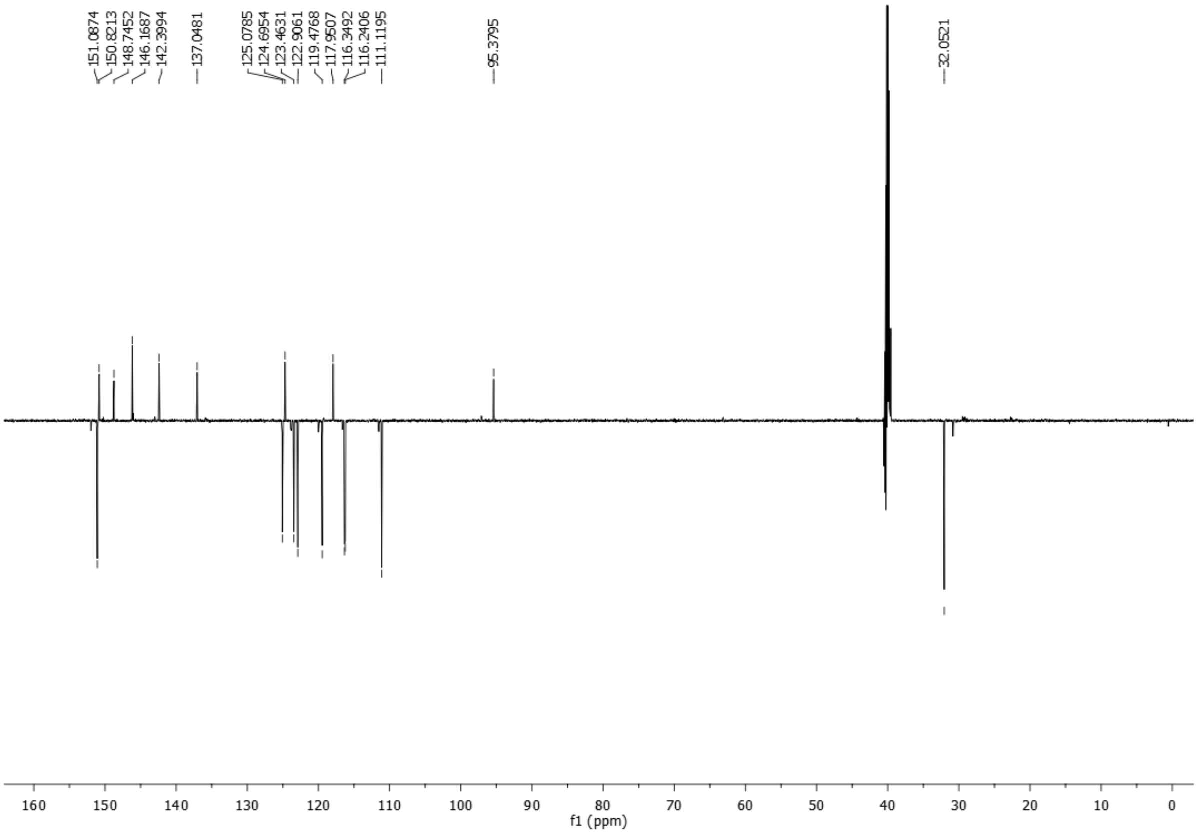

Figure S43. ^{1}H NMR spectrum (DMSO-d_6, 600 MHz) of ***(E)-2-(1-methyl-1H-benzo[d]imidazol-2-yl)-3-(3,4,5-trihydroxyphenyl)acrylonitrile 38***

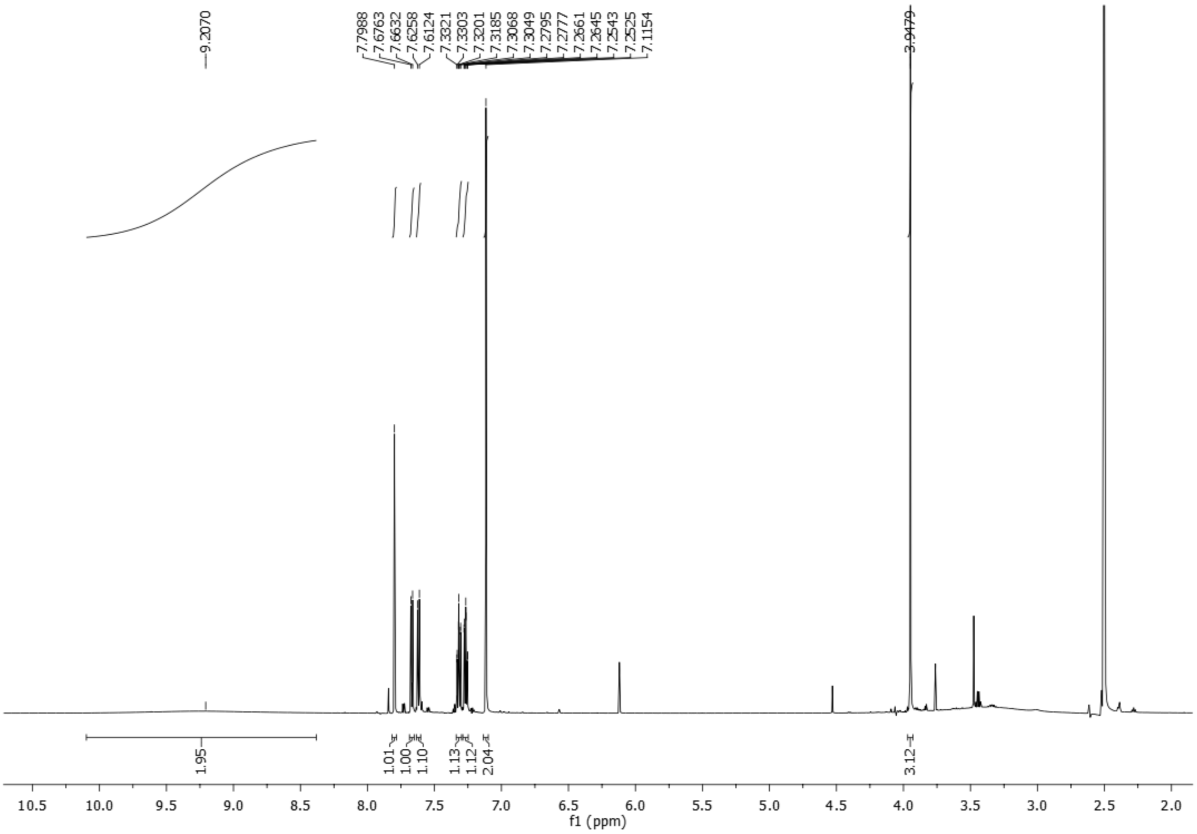

Figure S44. ^{13}C NMR spectrum (DMSO-d_6, 151 MHz) of ***(E)-2-(1-methyl-1H-benzo[d]imidazol-2-yl)-3-(3,4,5-trihydroxyphenyl)acrylonitrile 38***

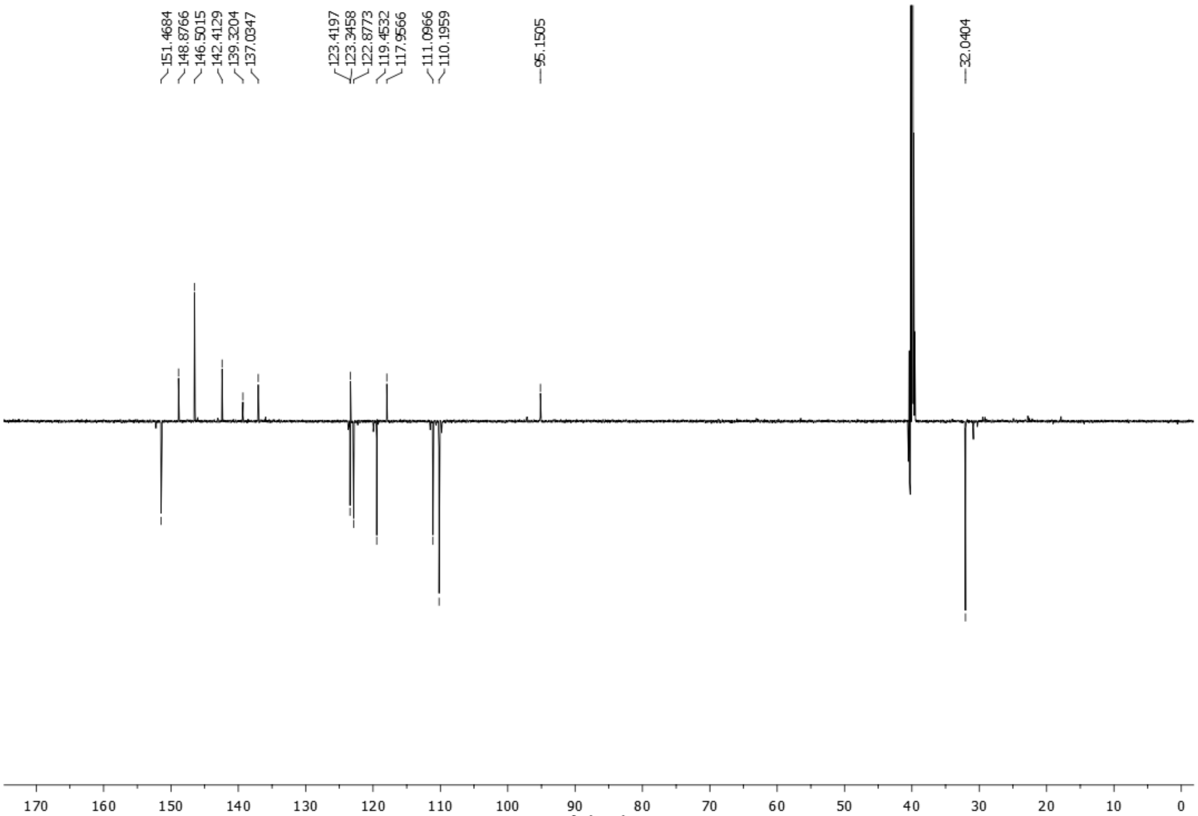

Figure S45. ^{1}H NMR spectrum (DMSO-d_6, 600 MHz) of ***(E)-2-(1-cyano-2-(4-hydroxyphenyl)vinyl)-1-methyl-1H-benzo[d]imidazole-6-carbonitrile 39***

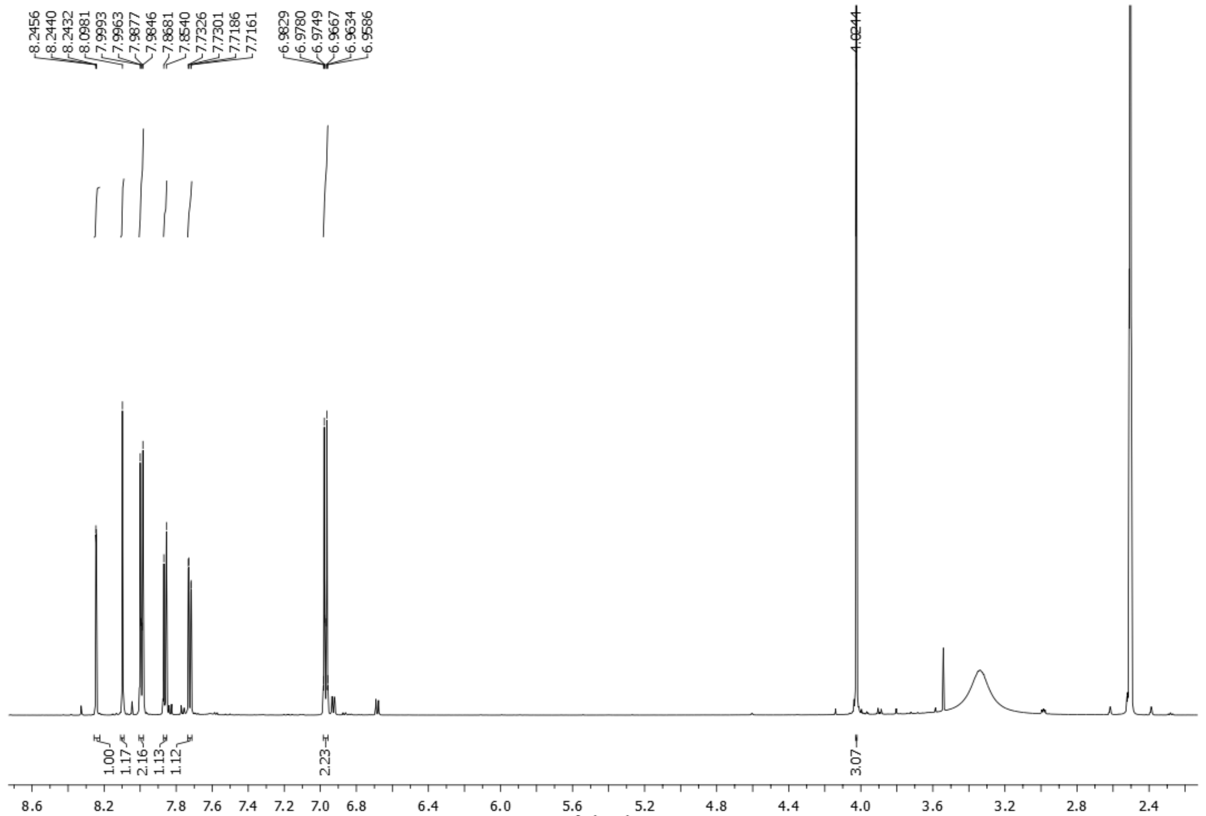

Figure S46. ^{13}C NMR spectrum (DMSO-d_6, 151 MHz) of ***(E)-2-(1-cyano-2-(4-hydroxyphenyl)vinyl)-1-methyl-1H-benzo[d]imidazole-6-carbonitrile 39***

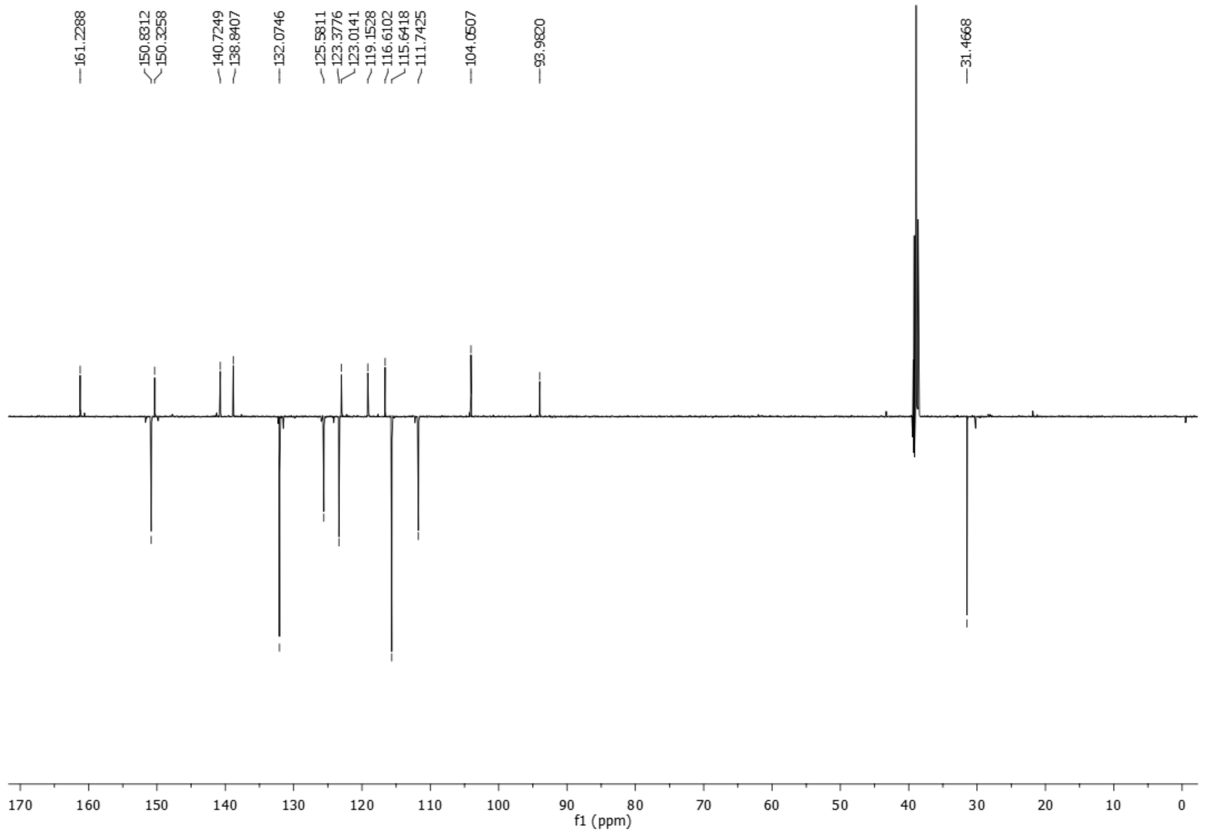

Figure S47. ^{1}H NMR spectrum (DMSO-d_6, 600 MHz) of ***(E)-2-(1-cyano-2-(3,4-dihydroxyphenyl)vinyl)-1-methyl-1H-benzo[d]imidazole-6-carbonitrile 40***

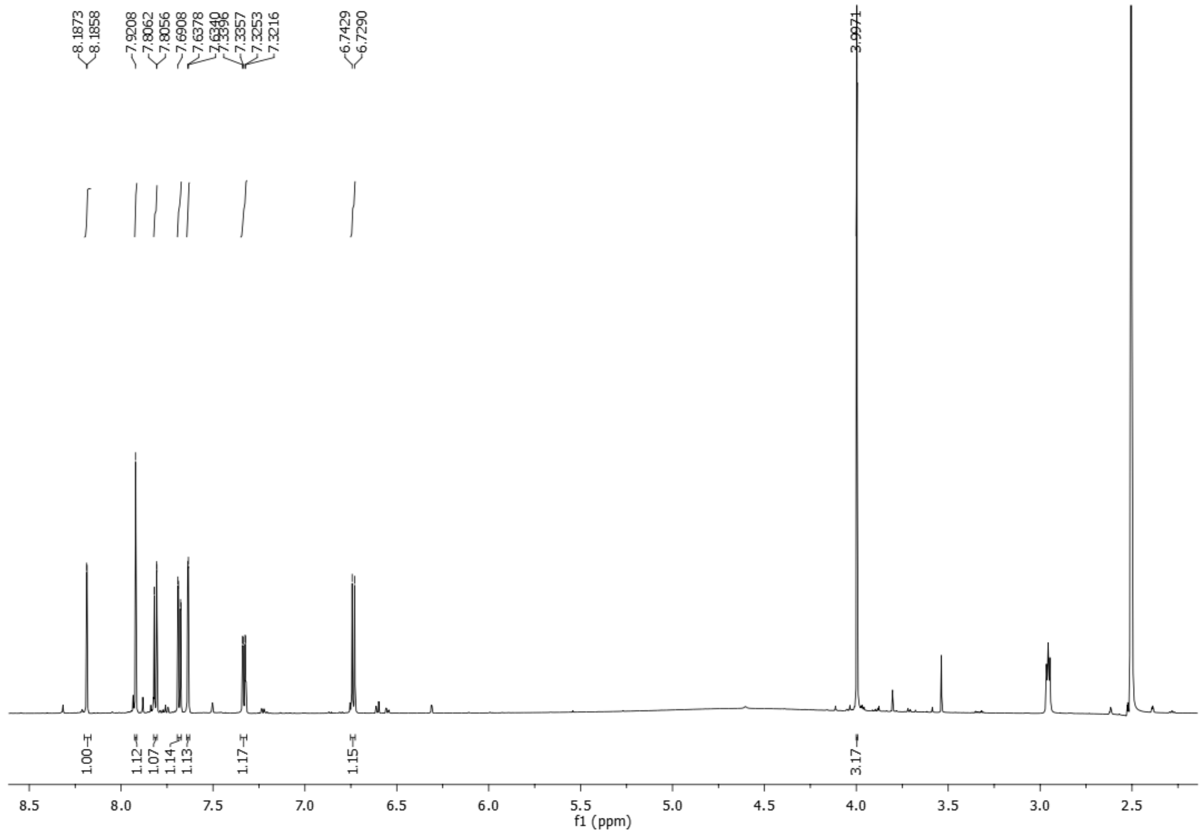

Figure S48. ^{13}C NMR spectrum (DMSO-d_6, 151 MHz) of ***(E)-2-(1-cyano-2-(3,4-dihydroxyphenyl)vinyl)-1-methyl-1H-benzo[d]imidazole-6-carbonitrile 40***

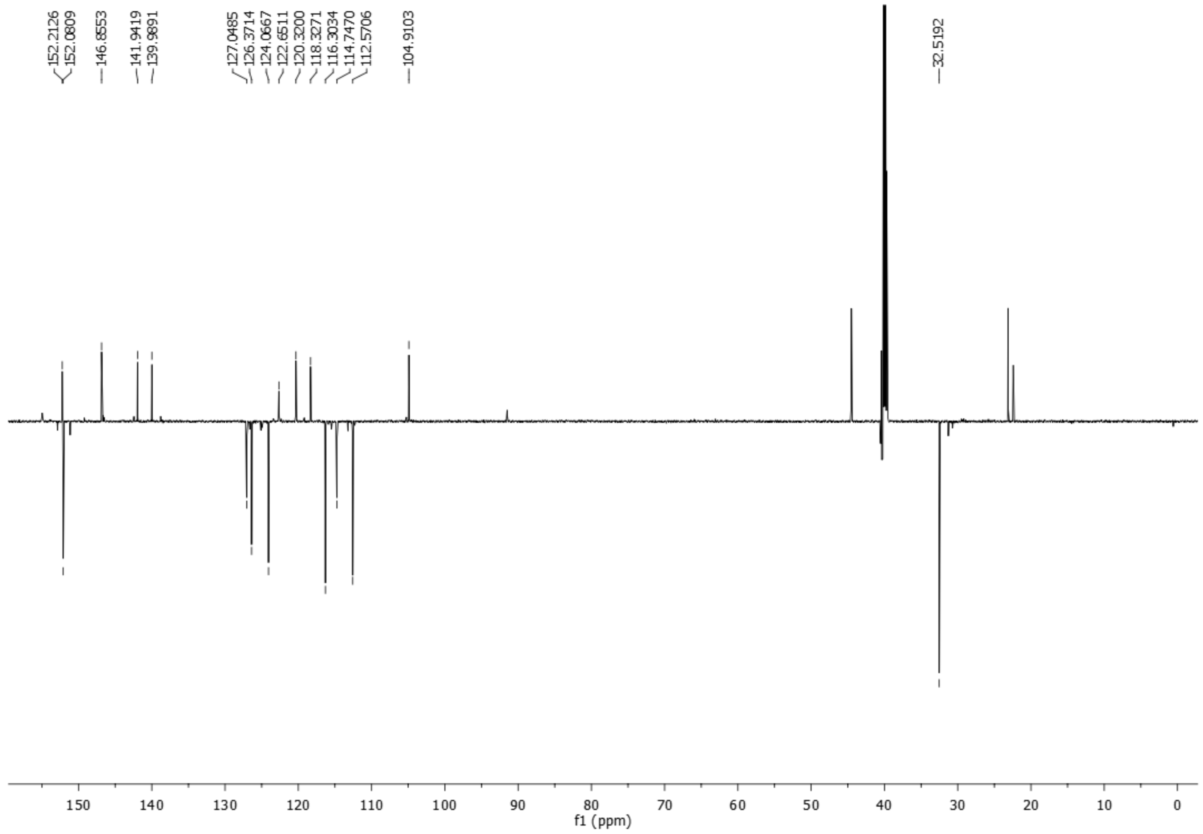

Figure S49. ^{1}H NMR spectrum (DMSO-d_6, 600 MHz) of ***(E)-2-(1-cyano-2-(3,4,5-trihydroxyphenyl)vinyl)-1-methyl-1H-benzo[d]imidazole-6-carbonitrile*** ***41***

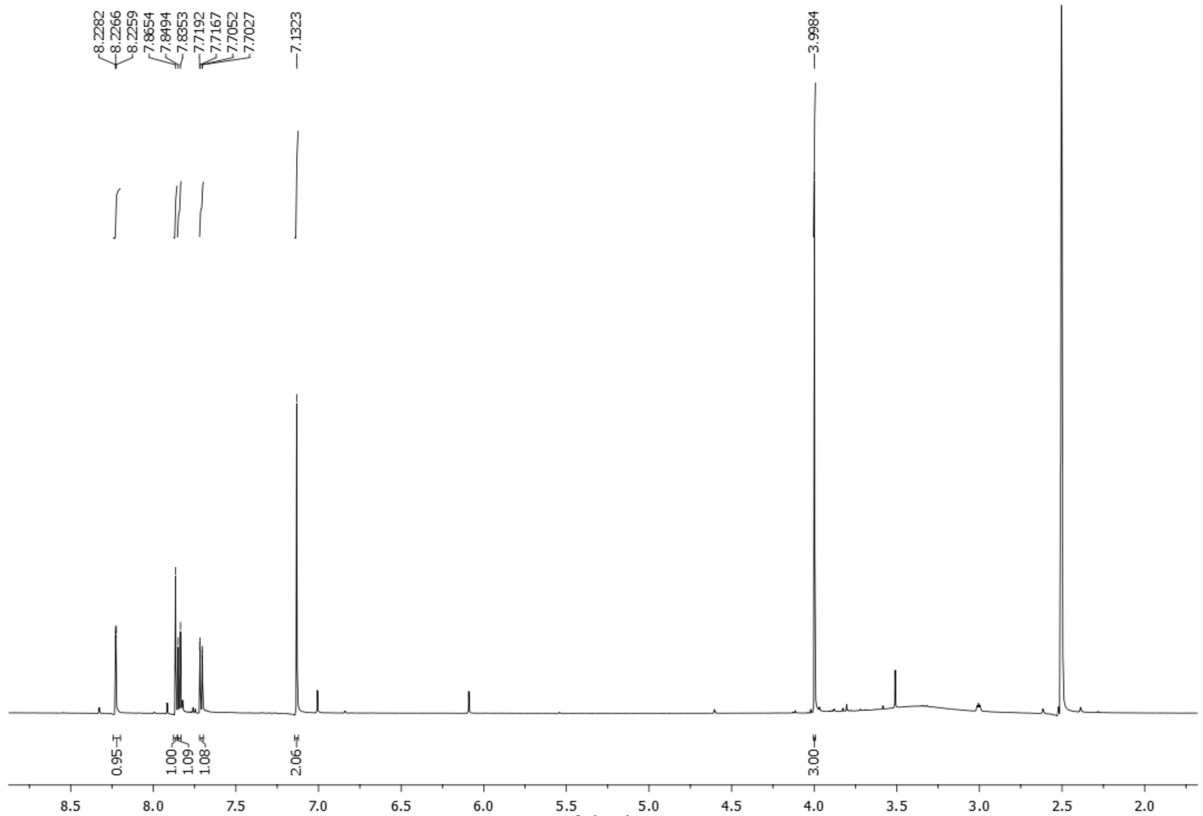

Figure S50. ^{13}C NMR spectrum (DMSO-d_6, 151 MHz) of ***(E)-2-(1-cyano-2-(3,4,5-trihydroxyphenyl)vinyl)-1-methyl-1H-benzo[d]imidazole-6-carbonitrile*** ***41***

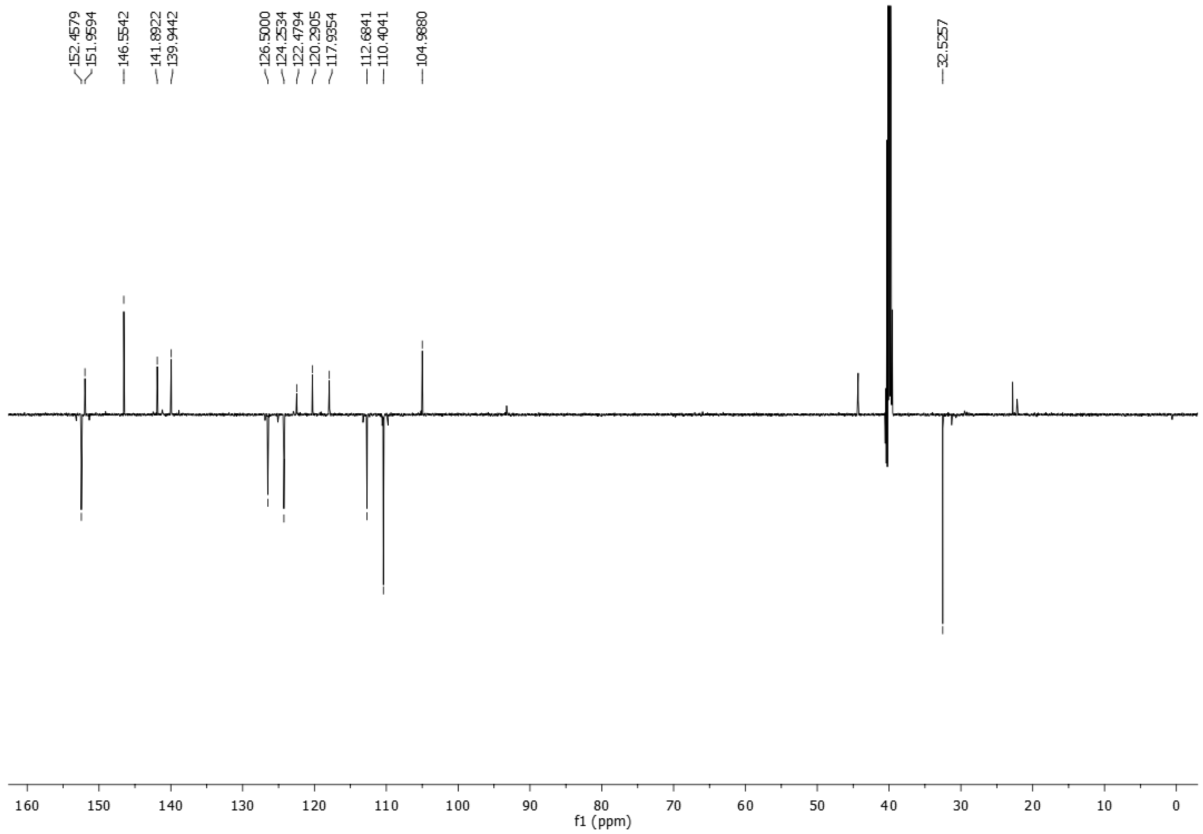

Figure S51. ^{1}H NMR spectrum (DMSO-d_6, 600 MHz) of ***(E)-3-(4-hydroxyphenyl)-2-(1-phenyl-1H-benzo[d]imidazol-2-yl)acrylonitrile*** ***42***

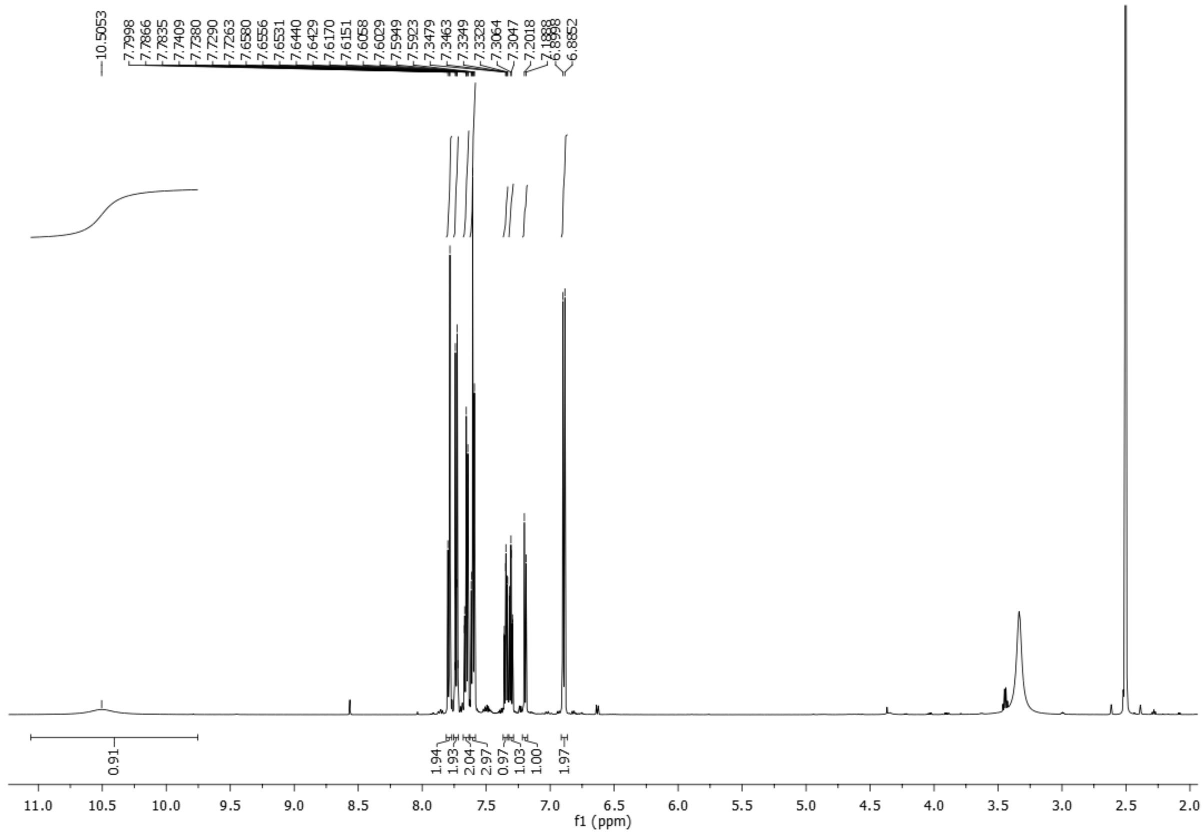

Figure S52. ^{13}C NMR spectrum (DMSO-d_6, 151 MHz) of ***(E)-3-(4-hydroxyphenyl)-2-(1-phenyl-1H-benzo[d]imidazol-2-yl)acrylonitrile*** ***42***

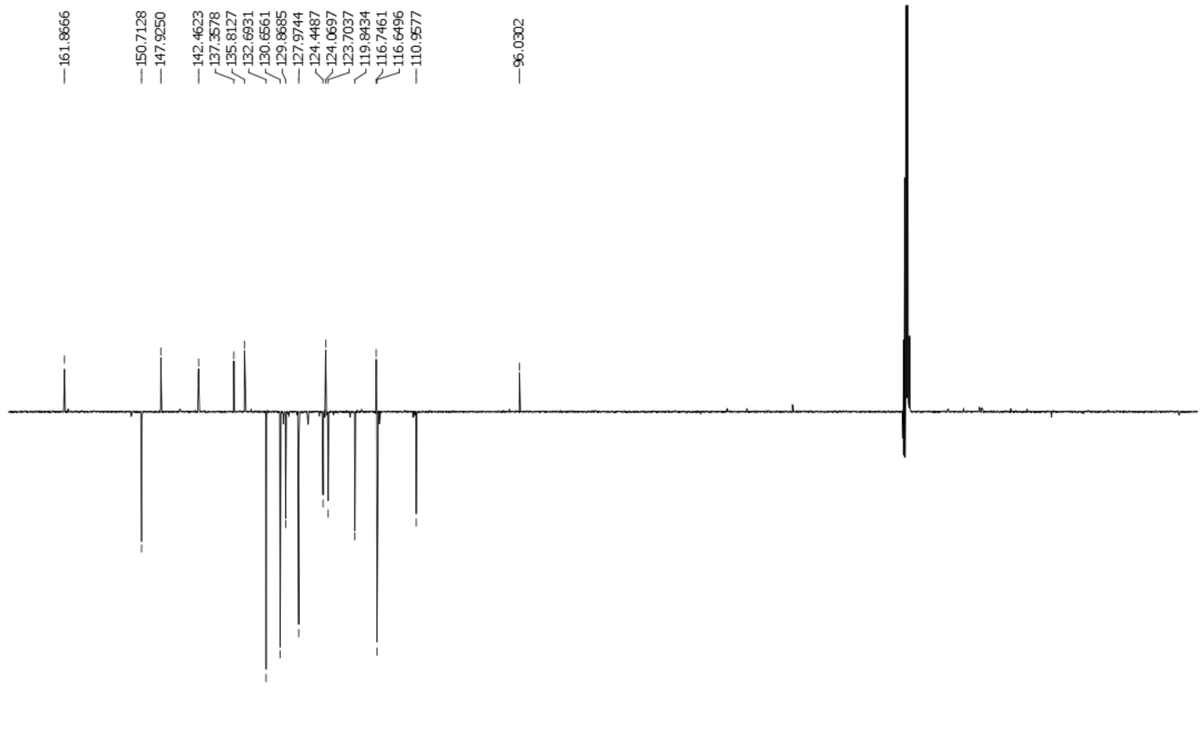

Figure S53. ^{1}H NMR spectrum (DMSO-d_6, 600 MHz) of ***(E)-3-(3,4-dihydroxyphenyl)-2-(1-phenyl-1H-benzo[d]imidazol-2-yl)acrylonitrile*** ***43***

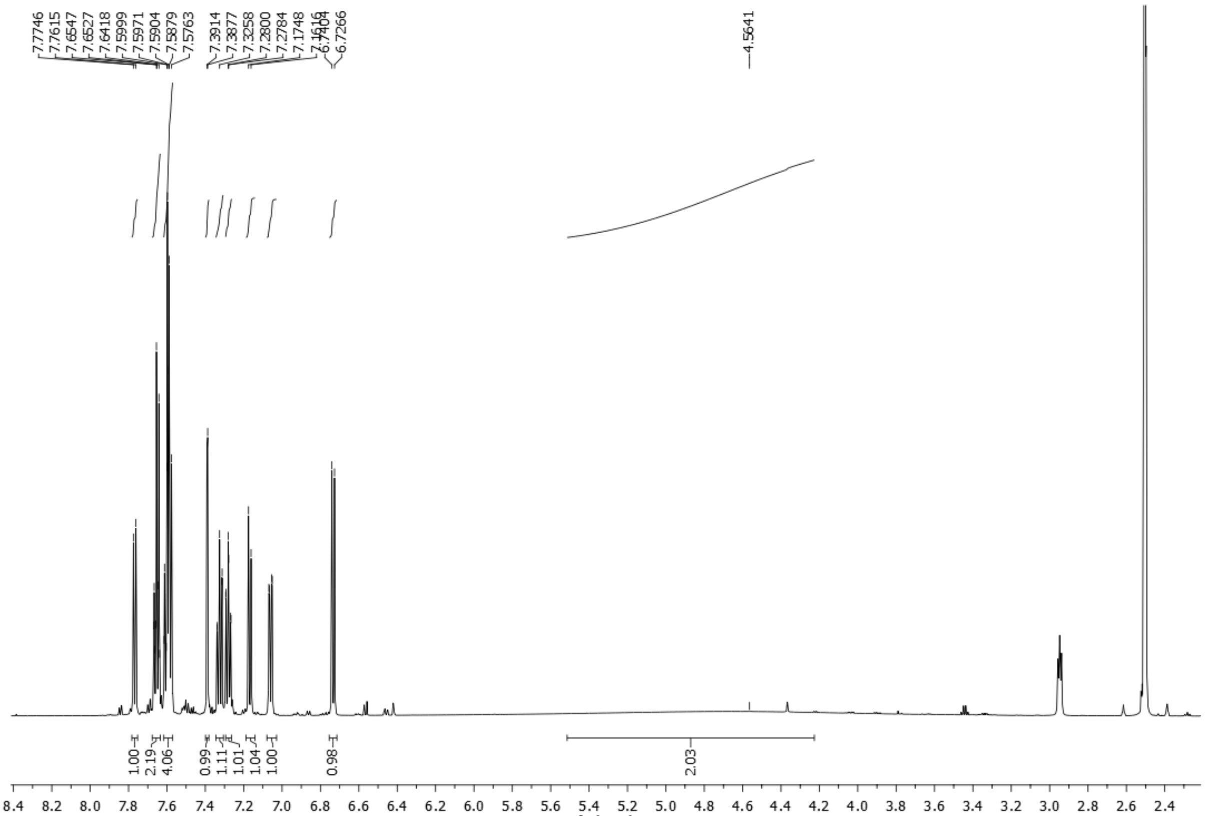

Figure S54. ^{13}C NMR spectrum (DMSO-d_6, 151 MHz) of ***(E)-3-(3,4-dihydroxyphenyl)-2-(1-phenyl-1H-benzo[d]imidazol-2-yl)acrylonitrile*** ***43***

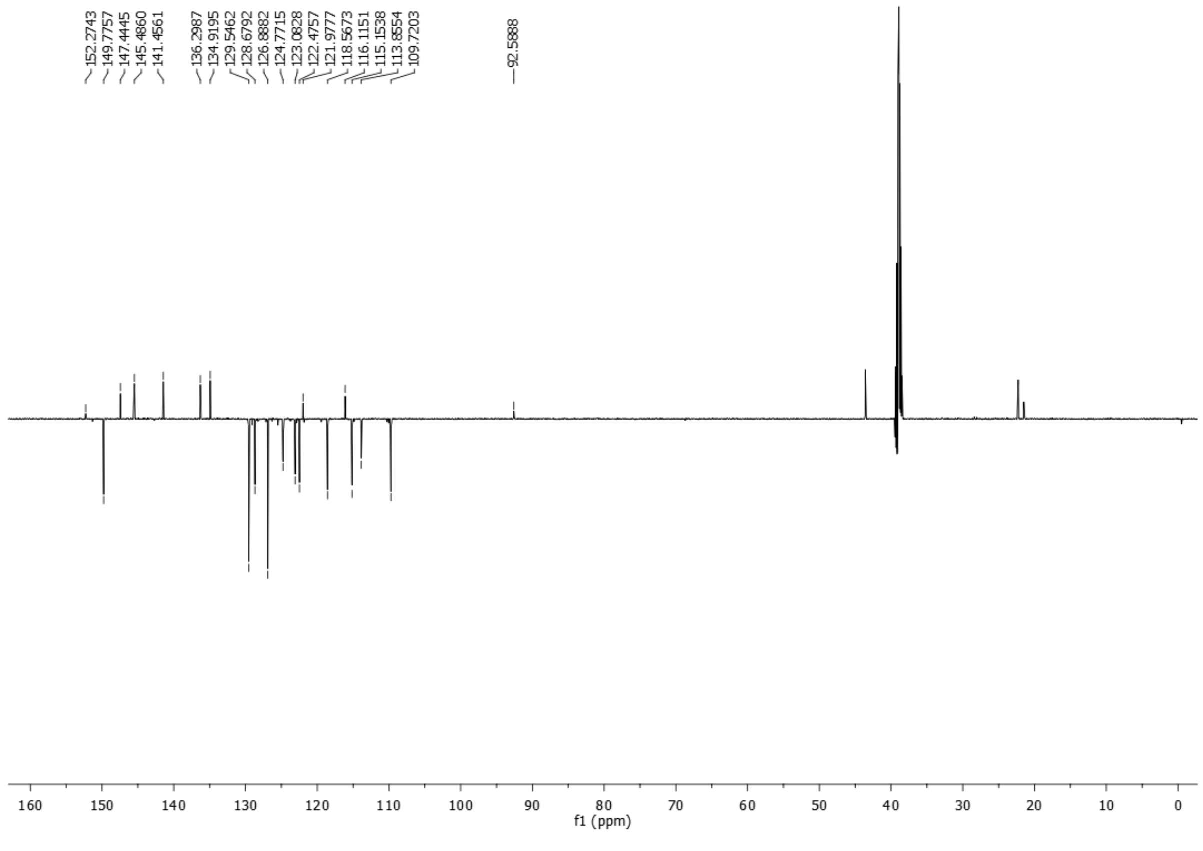

Figure S55. ^{1}H NMR spectrum (DMSO-d_6, 600 MHz) of ***(E)-2-(1-phenyl-1H-benzo[d]imidazol-2-yl)-3-(3,4,5-trihydroxyphenyl)acrylonitrile 44***

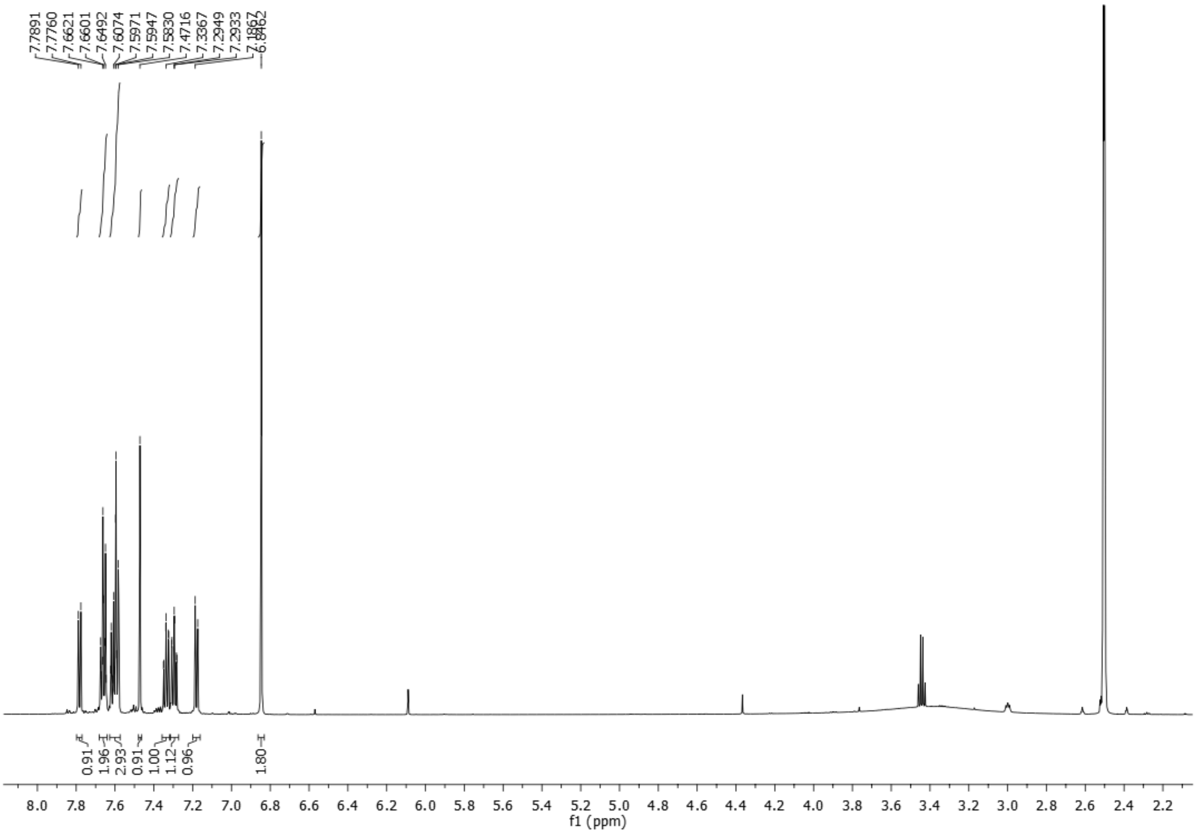

Figure S56. ^{13}C NMR spectrum (DMSO-d_6, 151 MHz) of ***(E)-2-(1-phenyl-1H-benzo[d]imidazol-2-yl)-3-(3,4,5-trihydroxyphenyl)acrylonitrile 44***

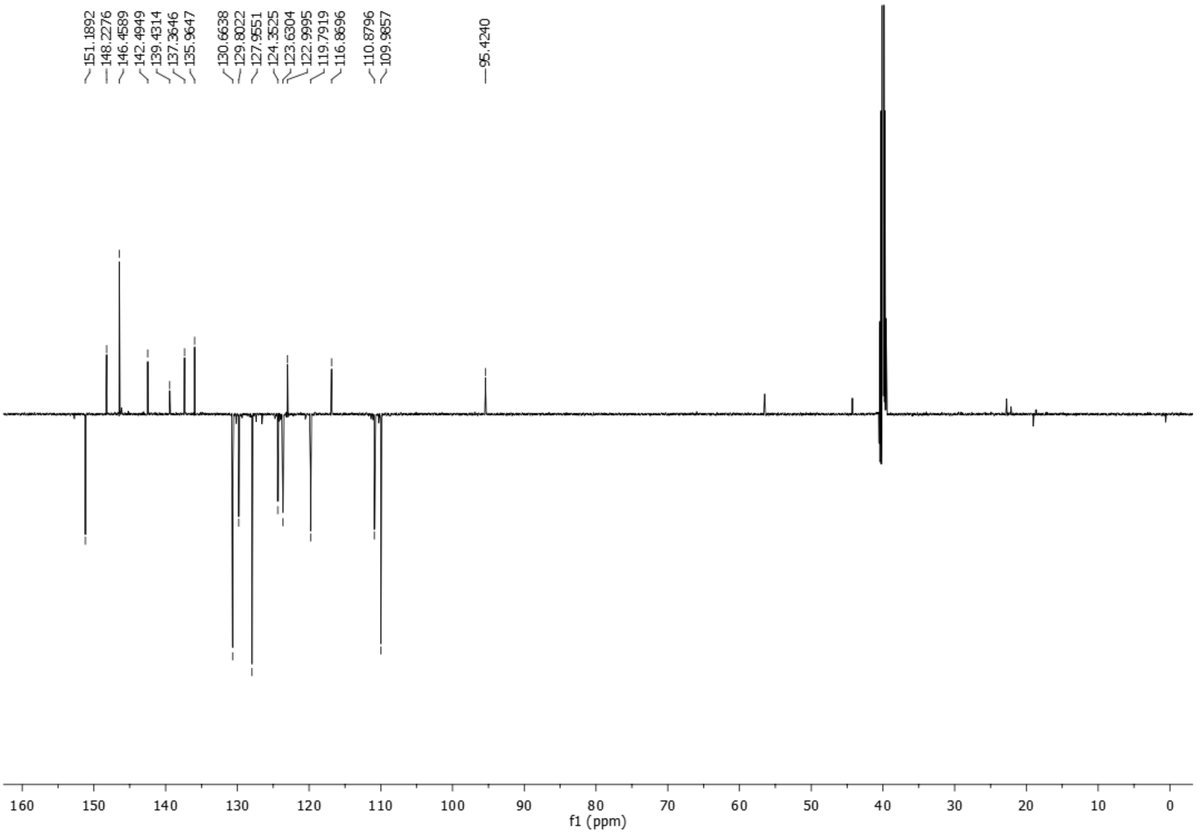

Figure S57. ^{1}H NMR spectrum (DMSO-d_6, 600 MHz) of ***(E)-2-(1-cyano-2-(4-hydroxyphenyl)vinyl)-1-phenyl-1H-benzo[d]imidazole-6-carbonitrile 45***

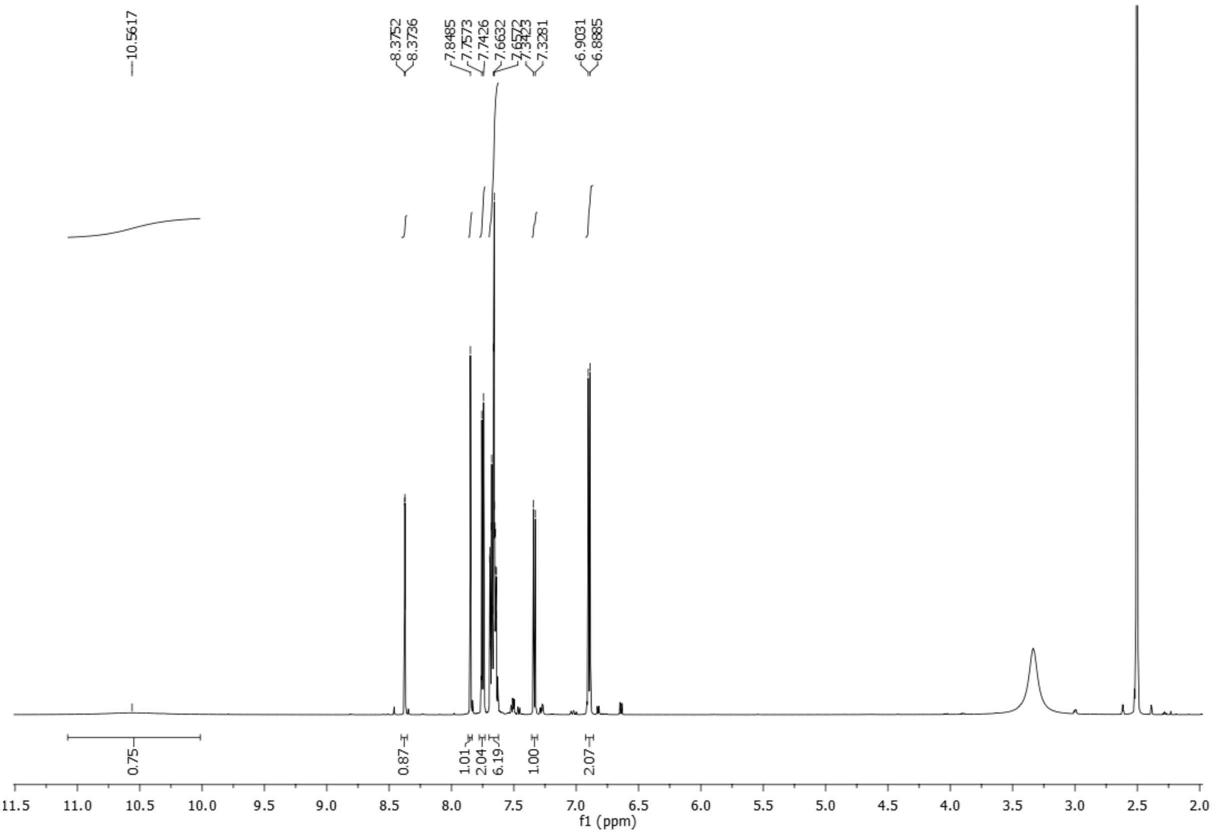

Figure S58. ^{13}C NMR spectrum (DMSO-d_6, 151 MHz) of ***(E)-2-(1-cyano-2-(4-hydroxyphenyl)vinyl)-1-phenyl-1H-benzo[d]imidazole-6-carbonitrile 45***

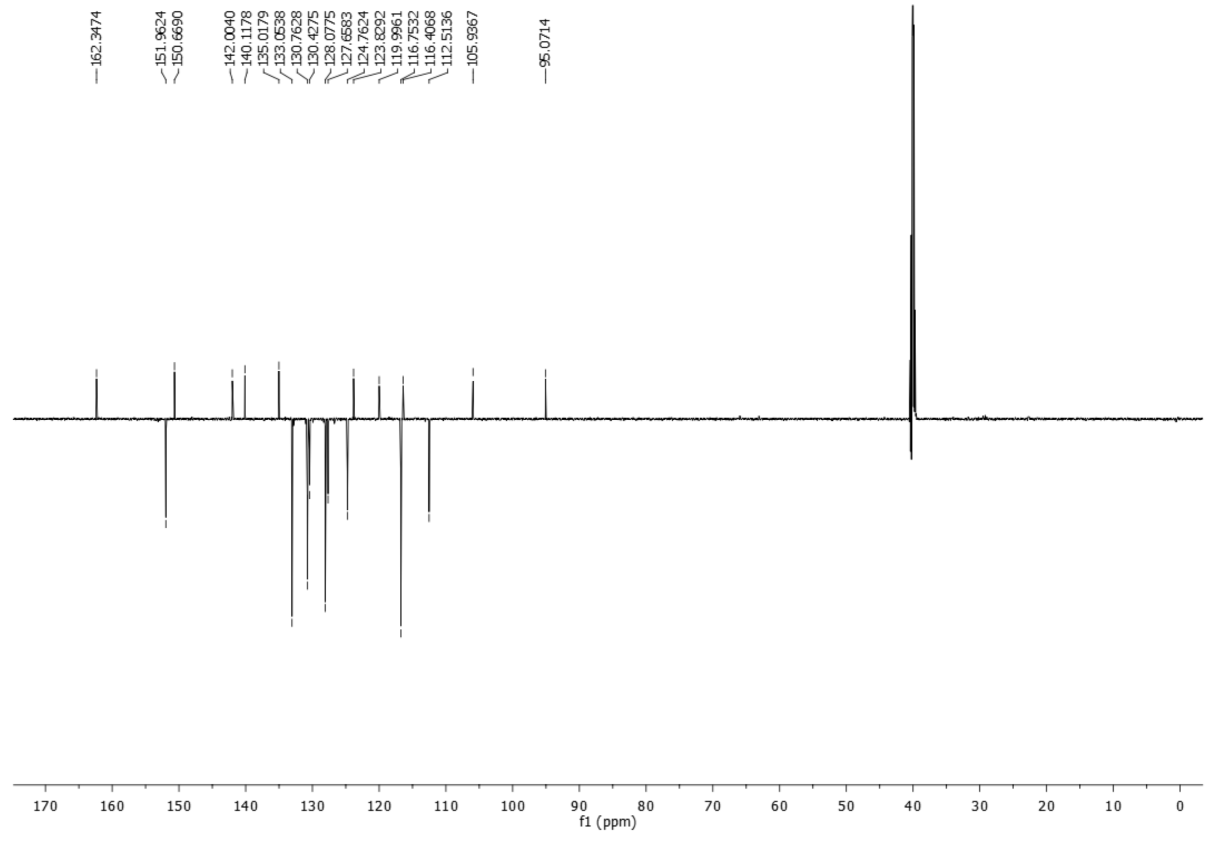

Figure S59. ^{1}H NMR spectrum (DMSO-d_6, 600 MHz) of ***(E)-2-(1-cyano-2-(3,4-dihydroxyphenyl)vinyl)-1-phenyl-1H-benzo[d]imidazole-6-carbonitrile 46***

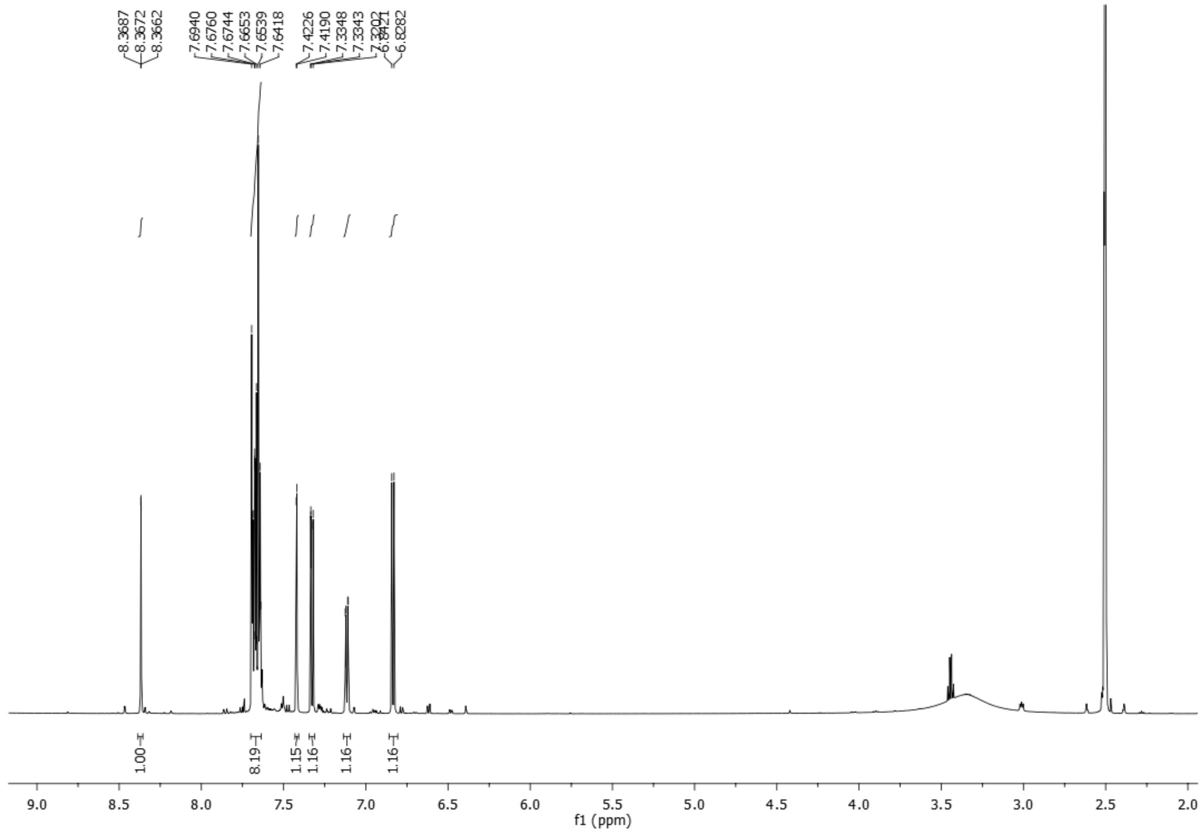

Figure S60. ^{13}C NMR spectrum (DMSO-d_6, 151 MHz) of ***(E)-2-(1-cyano-2-(3,4-dihydroxyphenyl)vinyl)-1-phenyl-1H-benzo[d]imidazole-6-carbonitrile 46***

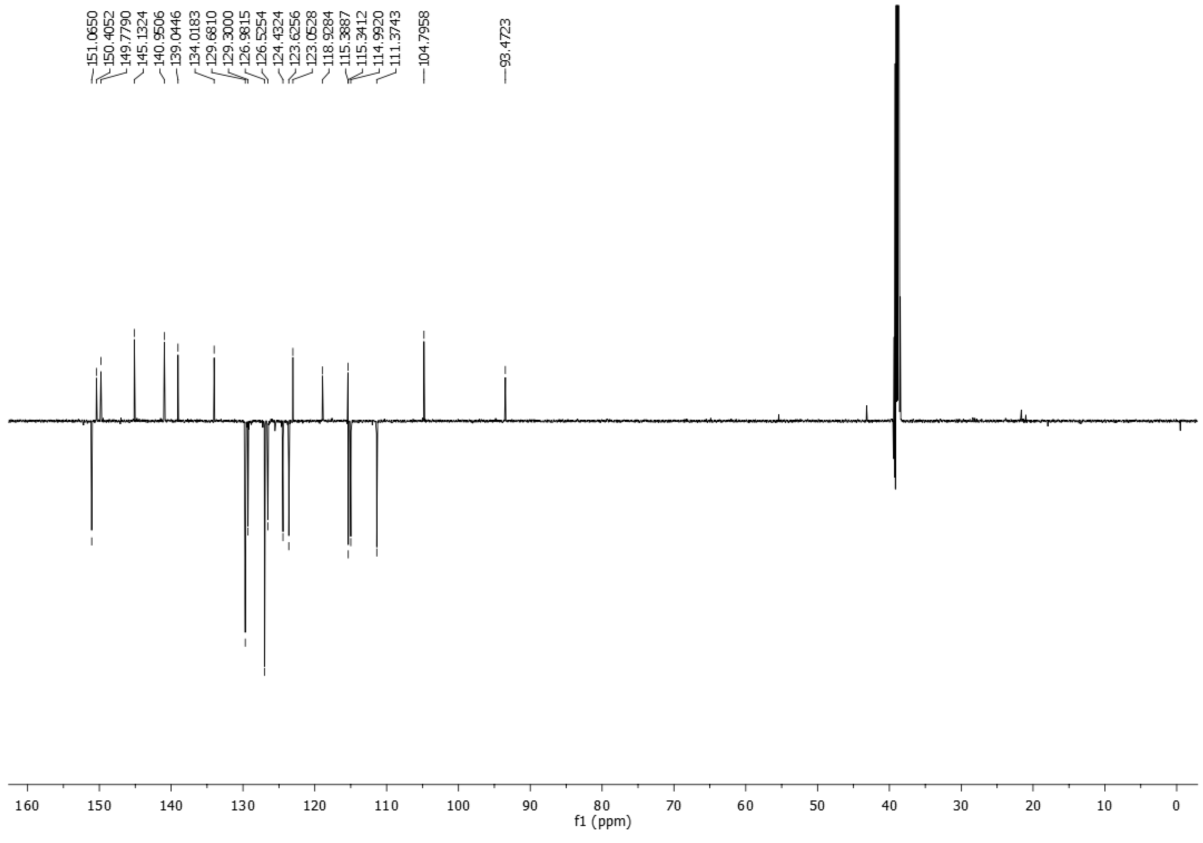

Figure S61. ^{1}H NMR spectrum (DMSO-d_6, 600 MHz) of ***(E)-2-(1-cyano-2-(3,4,5-trihydroxyphenyl)vinyl)-1-phenyl-1H-benzo[d]imidazole-6-carbonitrile*** ***47***

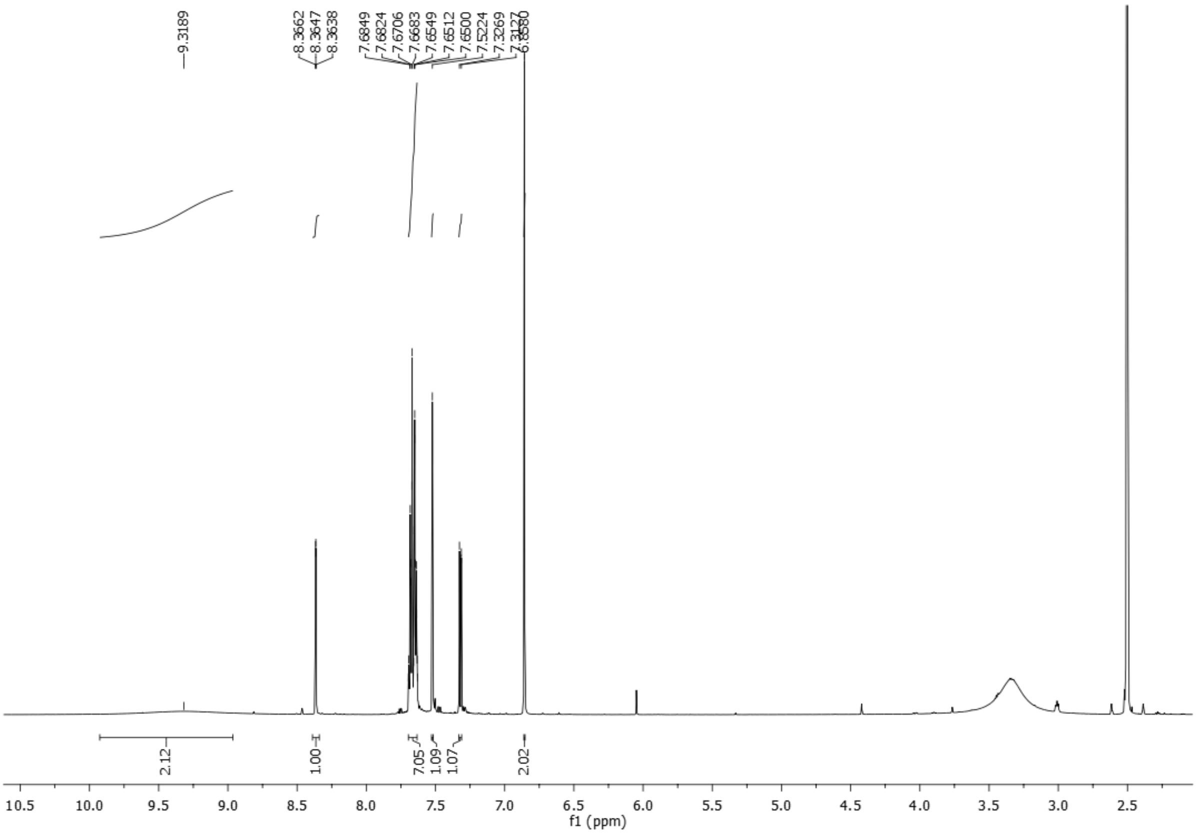

Figure S62. ^{13}C NMR spectrum (DMSO-d_6, 151 MHz) of ***(E)-2-(1-cyano-2-(3,4,5-trihydroxyphenyl)vinyl)-1-phenyl-1H-benzo[d]imidazole-6-carbonitrile*** ***47***

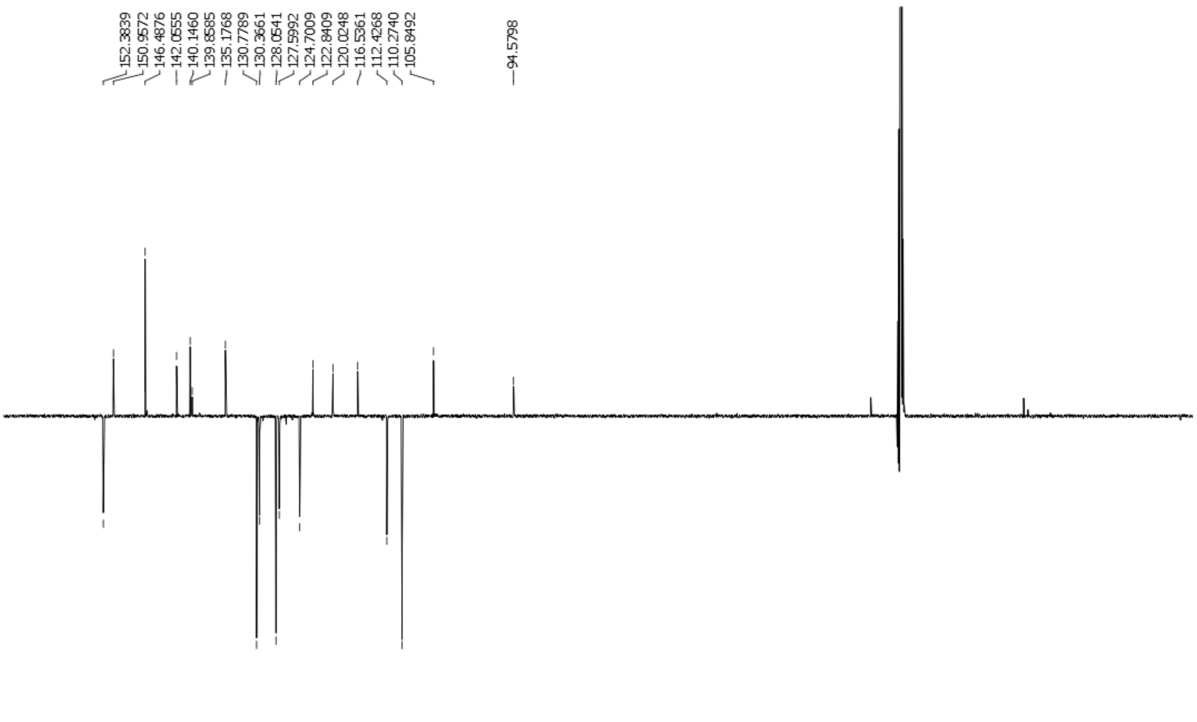

Figure S63. Reaction scheme for preparing the compounds **1-6** which synthesis has been already published.

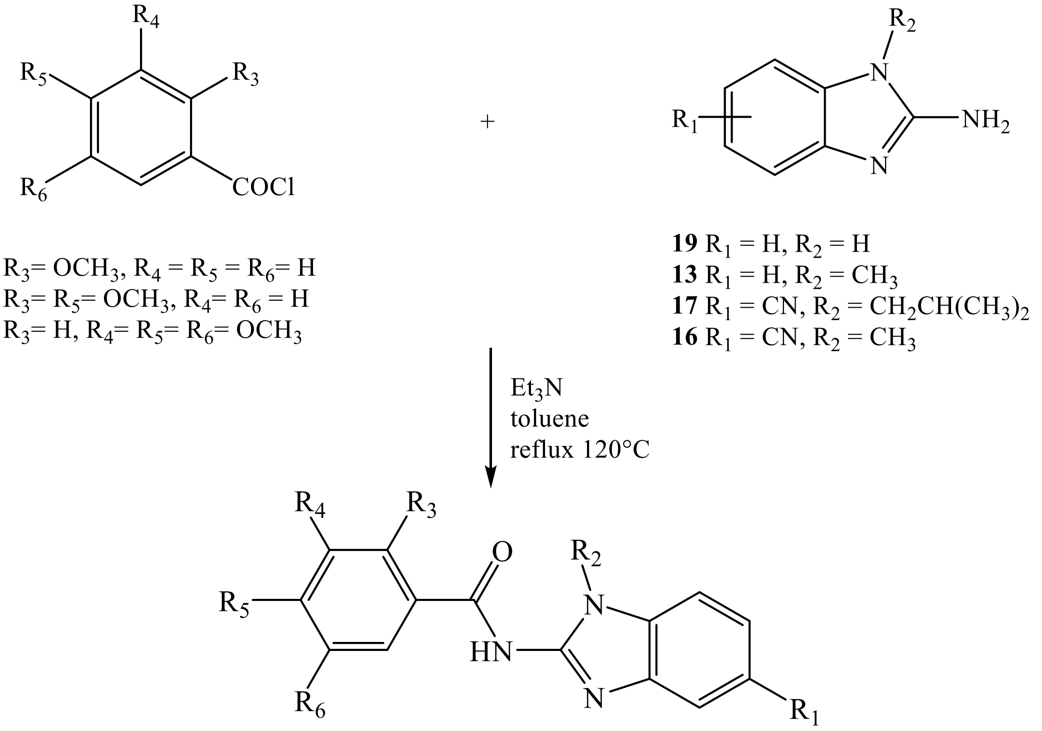

20 R_3= OCH_3, R_4 = R_5 = R_6= H
21 R_3= R_5= OCH_3, R_4= R_6 = H
22 R_3= H, R_4= R_5= R_6= OCH_3

19 R_1 = H, R_2 = H
13 R_1 = H, R_2 = CH_3
17 R_1 = CN, R_2 = $CH_2CH(CH_3)_2$
16 R_1 = CN, R_2 = CH_3

1 R_1 = H, R_2 = H, R_3 = R_5 = OCH_3, R_4 = R_6 = H
2 R_1 = H, R_2 = H, R_3 = H, R_4 = R_5 = R_6 = OCH_3
3 R_1 = H, R_2 = CH_3, R_3 = H, R_4 = R_5 = R_6 = OCH_3
4 R_1 = CN, R_1 = $CH_2CH(CH_3)_2$, R_3 = R_5 = OCH_3, R_4 = R_6 = H
5 R_1 = CN, R_2 = CH_3, R_3 = OCH_3, R_4 = R_5 = R_6 = H
6 R_1 = CN, R_2 = CH_3, R_3 = R_5 = OCH_3, R_4 = R_6 = H
